# Fast volumetric multifocus structured illumination microscopy of subcellular dynamics in living cells

**DOI:** 10.1364/BOE.516261

**Published:** 2024-03-11

**Authors:** Maximilian Lukas Senftleben, Antone Bajor, Eduardo Hirata, Sara Abrahamsson, Hjalmar Brismar

**Affiliations:** 1 Department of Applied Physics, KTH Royal Institute of Technology, Science for Life Laboratory, Stockholm, Sweden; 2 Baskin School of Engineering, University of California Santa Cruz, 1156 High Street, Santa Cruz, 95064, CA, USA

## Abstract

Studying the nanoscale dynamics of subcellular structures is possible with 2D structured illumination microscopy (SIM). The method allows for acquisition with improved resolution over typical widefield. For 3D samples, the acquisition speed is inherently limited by the need to acquire sequential two-dimensional planes to create a volume. Here, we present a development of multifocus SIM designed to provide high volumetric frame rate by using fast synchronized electro-optical components. We demonstrate the high volumetric imaging capacity of the microscope by recording the dynamics of microtubule and endoplasmatic reticulum in living cells at up to 2.3 super resolution volumes per second for a total volume of 30 × 30 × 1.8 µm^3^.

## 1. Introduction

Fluorescence microscopy is a central tool in cell biology providing images of subcellular structures with high resolution, contrast and biomolecule specificity. In a conventional microscope the resolution is limited by diffraction, defined by the Abbe diffraction limit [1]. In recent decades, super resolution microscopy (SRM) has enabled scientists to resolve structures well below this limit.

SRM can be classified into three techniques: Stimulated emission depletion microscopy (STED) [2], Single Molecule Localization Microscopy (SMLM) [3], and Structured illumination microscopy (SIM). STED and SMLM bypass the diffraction limit and can increase the resolution over widefield and confocal microscopy more than tenfold. However, their use for imaging living organisms and fast dynamic biological processes is limited due to the point scanning (STED), or sequential acquisition of individual fluorophores (SMLM) requiring long acquisition times. These techniques also often require introduction of specific genetically modified fluorescent proteins or specialized dyes for switching and depletion. By contrast, SIM offers a twofold resolution increase laterally [4] and axially [5] and is compatible with many fluorescent proteins or fluorescent dyes.

In SIM, a sinusoidal grating pattern with a period close to the diffraction limit is projected onto the sample. The resulting fluorescence is also sinusoidal patterned and down-modulates otherwise unobservable high spatial frequency information into the support of the optical transfer function (OTF). The higher frequency components can subsequently be extracted in Fourier space by computational reconstruction from raw images recorded for different rotations and phases of the sinusoidal pattern. 9 raw images are needed for 2D-SIM [4] and 15 raw images for 3D-SIM [5]. The first generation of SIM microscopes used fixed optical gratings that were physically rotated and translated to generate the sinusoidal pattern on the sample. Today, pixelated liquid crystal Spatial Light Modulators (SLM) are used since they outperform physical gratings in terms of speed, accuracy and adjustment capabilities [6,7]. Typical SLMs can switch the pattern with a frequency of up to 1 kHz and can be easily synchronized with other electrical components of a SIM. Therefore, for single plane 2D-SIM, the acquisition speed is now only technically limited by the acquisition rate of the camera [8], providing the sample is bright enough. This allows the observation of millisecond time-scale cellular dynamics with high spatial-temporal resolution [9]. However, for volumetric 3D imaging the microscope still needs to be refocused to record images at different depths of the sample. The sequential recording of 2D planes slows the volumetric acquisition rate proportionally to the number of planes required. Recently, several techniques have been developed that are capable of recording multiple 2D planes within a single camera exposure and thereby removing the need to refocus the microscope to record an image volume.

The method introduced by Geissbuehler et.al. [10] uses an image splitting prism in the emission path, so that the emitted fluorescence from eight focal-planes arrives simultaneously at distinct positions on two camera sensors. This detection technique was combined with structured illumination using a digital mirror device (DMD) to generate the grating pattern and was used to perform 3D live-cell imaging of COS-7 mitochondria network dynamics [11], achieving a volumetric speed of 1.3 SIM volumes per second. In another approach [12], four separate cameras have been used to record different focal planes at four focal positions of the sample without moving the stage based on their defocus distance.

Multifocus Microscopy (MFM) [13], is also achieved by separating fluorescence from different focal planes on the camera sensor, but is accomplished using diffractive Fourier optics to obtain aberration-free refocusing. For this, a custom-made refocusing and aberration-correcting diffraction grating is placed in the Fourier plane of the microscope and is combined with chromatic dispersion correcting optics. The emission beam is multiplexed into multiple separate focal beams, each registered on a separate segment on the camera sensor. Only a single exposure of the camera is therefore needed to capture a full 3D volume. In a proof-of-concept study MFM was combined with structured illumination microscopy (MF-SIM) [14]. However, in this implementation, the acquisition speed was limited to a volumetric acquisition rate of less than 1 Hz as it was adapted onto a first-generation SIM microscope using a relatively slow optomechanical grating.

Here, we report on an adaption of MF-SIM where the illumination and detection beam paths have been designed to maximize the volume acquisition speed while retaining optimal spatial sampling for a high SIM resolution. With this setup, we demonstrate 3D subdiffraction imaging of subcellular dynamics at high volumetric imaging rates of up to 2.3 full MF-SIM volumes per second.

## 2. Results

### 2.1 Construction of MF-SIM

**Fig. 1.**
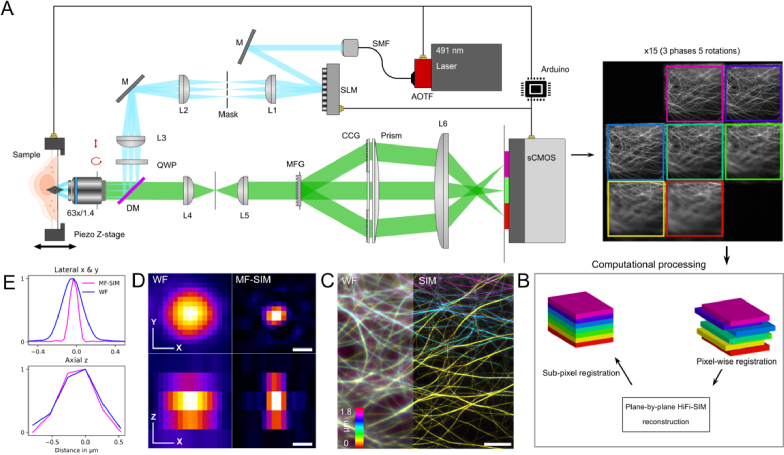
Schematic overview of the MF-SIM. (A) On the excitation side, a 491 nm laser was modulated with an AOTF and mode-cleaned with a single mode fiber. The illumination light is collimated onto a nematic SLM, creating the SR-SIM pattern on the sample through a 63x/1.4 NA objective. A z-axis piezo stage is used for fast axial scanning if higher axial sampling or range is desired. The emission is collected through the same objective and is focused onto the multifocus diffraction grating (MFG) that multiplexes the emission beam into 7 distinct focal planes that are projected onto the same sCMOS camera chip. Other abbreviations: SMF single mode fiber, M mirror, L lens, QWP quarter wave plate, DM dichroic mirror, CCG chromatic correction grating. (B) The raw SIM focal planes are cropped and roughly aligned into a stack which is then reconstructed plane-by-plane. After that, the SIM images are registered to constitute a super-resolution MF-SIM volume. (C) Representative depth color-coded Widefield (WF) vs. MF-SIM image to demonstrate the axial contrast improvement and lateral resolution improvement of microtubule cytoskeleton, xy-plane is shown. (D) Representative point spread function (PSF) of a 100 nm fluorescent bead in Widefield (WF) mode (left) and in MF-SIM mode (right) in the lateral xy-plane (upper) and in the axial xz-plane (lower). In the axial plane the PSF image was adjusted so that the dimensions are isotropical, as the axial spacing inherently set by the MFG was 264 nm. The full width half maximum for the widefield mode was measured to be x: 272 +/- 9 nm, y: 267 +/- 8 nm, z: 576 +/- 29 nm and for the MF-SIM mode: x: 110 +/- 4 nm, y: 100 +/- 4 nm, z: 542 +/- 16 nm with n = 95 beads. (E) Normalized intensity profile along the PSF maximum for the lateral plane (x & y plane averaged) and axial plane. Scale bars 4 µm in (C) and 260 nm in (D).

To acquire a 3D volume with a microscope, the focus needs to be shifted along the z-axis. This is typically done by moving the sample relative to the focus of the objective (or vice versa). However, this is not needed in MF-SIM where multiple z-planes with different focus in the sample are registered on the camera sensor simultaneously. MF-SIM can therefore provide faster volumetric imaging than conventional techniques. We have here constructed a MF-SIM that is fast enough to acquire data for the reconstruction of 7.0 full SIM volumes per second at its technical limit (with an exposure time of 1 ms and a readout time of 8.5 ms), covering a volume of 30 × 30 × 1.8 µm^3^ (Fig. 1(A)). To achieve a high recording speed, we used a fast nematic SLM to generate the structured illumination pattern. Further, an acousto-optical tunable filter (AOTF) was used for fast on/off light switching and a fast scientific Complementary Metal–Oxide–Semiconductor (sCMOS) camera was used for detecting fluorescence. All components were synchronized by a microcontroller to minimize delays. A detailed timing diagram can be found in Supplement 1. With this configuration the theoretical MF-SIM recording speed is only limited by the readout speed of the camera which roughly amounts to 8.5 ms.

We compared the performance of 2-beam MF-SIM (with 9 raw frames per timepoint) and 3-beam MF-SIM (with 15 raw frames per timepoint) and found that 3-beam MF-SIM was superior due to increased axial contrast after reconstruction (Supplement 1). Therefore, all subsequent datasets in this paper were acquired with 3-beam MF-SIM and 15 raw images per timepoint (except in previously mentioned Supplement 1).

The SLM is controlled to sequentially generate the 15 SIM stripe patterns (5 phases and 3 rotations) and synchronized with the camera exposure. The MFM optics project the fluorescence from 7 focal planes on separate parts of the sCMOS camera. The 7 plane MFG was chosen as it yields more emission light per plane as compared to the 9 plane MFG [14,15] and the total focal volume consisting of 7 planes spaced 264 nm apart is sufficient to image the cultured mammalian cells used in this study.

To make a MF-SIM reconstruction of the data the raw images are first re-registered as the separate focal planes and then processed for SIM reconstruction. The first step in image processing is thus to crop the raw images and separate the different focal planes into a volume stack. Each focal plane is then reconstructed with HiFi-SIM [16] to gain lateral resolution improvement (Fig. 1(B)) and improve axial contrast. Next, the stack is laterally aligned to yield a super-resolution 3D MF-SIM volume (Fig. 1(C)). Raw and processed data is available in Dataset 1, Ref. [38].

To verify the resolution improvement, we compared the point spread function (PSF) between MF-SIM and widefield mode. We imaged 100 nm fluorescent beads mounted on a glass coverslip (Fig. 1(D)) in widefield mode where the SLM was not displaying a SIM grating. We imaged the same region of interest again with MF-SIM and compared the full width half maximum (FWHM) along the center of the bead for the axis of x, y and z. The FWHM for the widefield mode was measured to be x: 272 +/- 9 nm, y: 267 +/- 8 nm, z: 576 +/- 29 nm and for the MF-SIM mode: x: 110 +/- 4 nm, y: 100 +/- 4 nm, z: 542 +/- 16 nm after averaging the intensity profiles of 95 beads. The lower FWHM values along the x and y axis for the MF-SIM mode indicate improved lateral resolution. As expected, the axial resolution is not improved through MF-SIM, as the FWHM values along the z axis are similar.

### 2.2 MF-SIM performance in biological samples

**Fig. 2.**
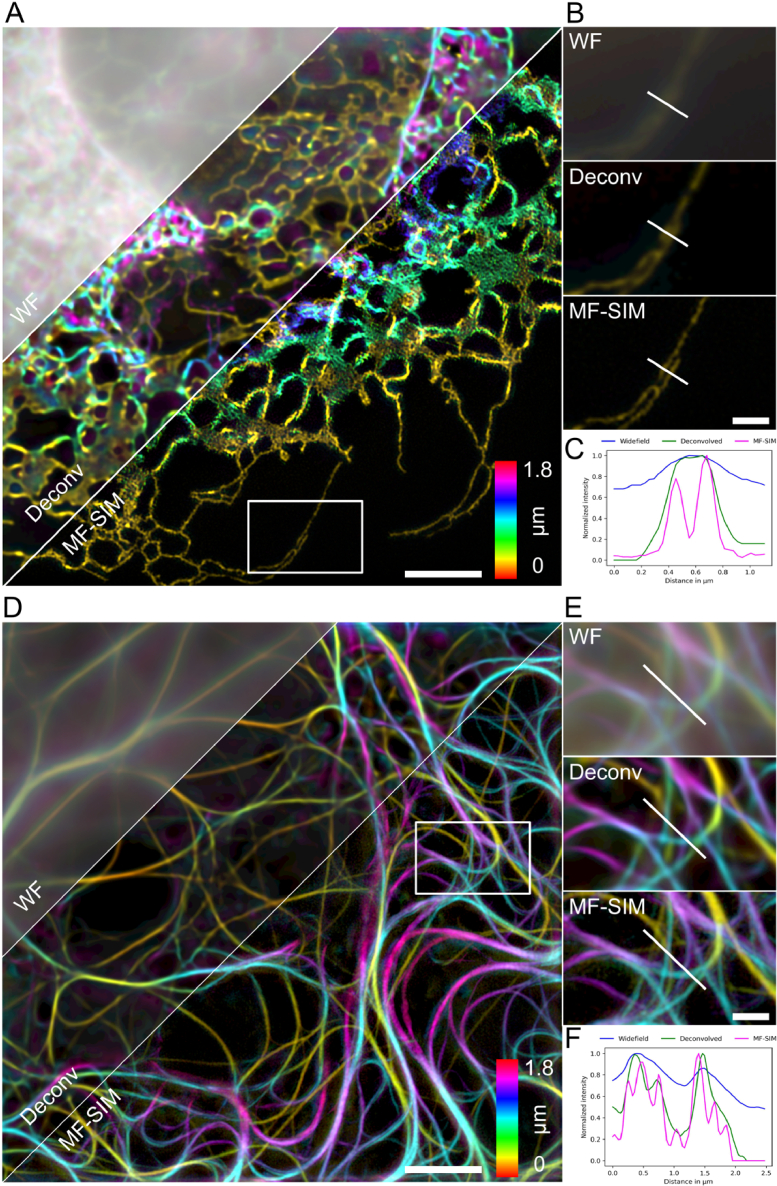
MF-SIM of biological samples to demonstrate improved axial contrast and lateral resolution over widefield microscopy. (A) Color-coded maximum intensity projection of Widefield (WF), Deconvolved (WF Deconv) and MF-SIM image of the ER-network in COS-7 cells. (B) Magnified regions of white box in (A) show individual ER tubule. (C) Normalized intensity profile along the white line in (B). (D) Equivalent to (A) WF, WF Deconv and MF-SIM image of microtubule cytoskeleton in fixed NIH/3T3 cells. (E) Magnified regions of white box in (D) show complex microtubule arrangement with normalized intensity profile (F) along white line in (E). Scale bars in (A) & (D) are 4 µm and in (B) and (E) 1 µm. Total FOV for (A) and (D) 29 × 29 × 1.8 µm^3^, in all images the xy-plane is shown.

We first demonstrate the performance of MF-SIM in a biological sample by imaging the endoplasmic reticulum (ER) in cultured COS-7 cells. The ER is an intracellular organelle that consists of interconnected tubules and flat sheets with holes of varying size [17]. ER was labelled by transfecting the cells with a fluorescently tagged protein KDEL-StayGold (Fig. 2(A)-(C)) [18]. For imaging, we used a 20 ms exposure per raw MF-SIM frame which gave a total recording time of 428 ms for one full MF-SIM volume with a laser power density of 24 W/cm^2^. The raw images were processed to yield both a plane-by-plane reconstructed MF-SIM volume and the corresponding volume with 7 widefield images.

In the widefield images, the out of focus light is dominant and gives a strong background haze and low contrast. The haze is mostly removed through widefield deconvolution, however, the images still lack resolution to resolve the finer features of ER. In the SIM reconstructed images, it is however possible to separate the densely packed ER-strands (Fig. 2(B)-(C)).

Next, we demonstrate the resolution enhancement by imaging microtubules. The dynamic instability of microtubules, which is controlled by factors within the microtubule-associated proteins (MAPs) group, is an important regulatory process and has previously been studied with widefield and 2D-SIM techniques [19,20]. We imaged the microtubule cytoskeleton by transfecting NIH/3T3 cells with EMTB-3XGFP [21] (Fig. 2(D)-(F)) which was sufficiently bright without exhibiting cytosolic background fluorescence. The cells were fixed with paraformaldehyde and imaged using an exposure time of 100 ms per raw frame with a laser power density of ∼19 W/cm^2^. To provide additional axial sampling, we used a piezo stage to record for each timepoint two image volumes separated in the focus position by half the MFM focus distance (132 nm). The microtubule network spans, just like ER, the entire cell volume (Fig. 2(D)). Intersecting microtubules that cannot be resolved by widefield or deconvolution are clearly separated in the MF-SIM images (Fig. 2(E)-(F)). Raw and processed data is available in Dataset 2, Ref. [39].

### 2.3 Live-cell imaging

**Fig. 3.**
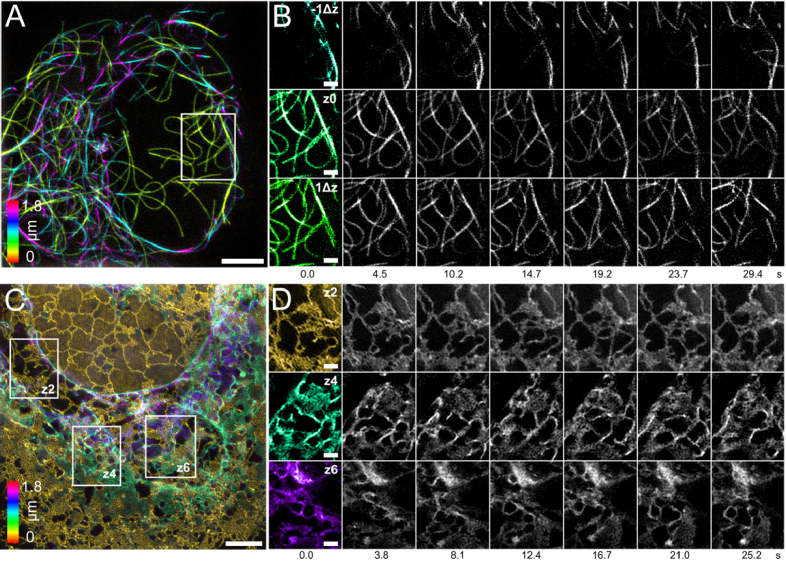
Dynamic MF-SIM imaging of cell organelles. (A) Color-coded maximum intensity projection of microtubule cytoskeleton observed over almost 30 seconds. Additional z-stepping provided extended axial sampling of Δz of 132 nm and resulted in volumetric imaging rate of ∼0.9 Hz. (B) Multiple timepoints of region of interest show the dynamics of individual microtubules at specific z-plane and its upper/lower neighboring z-plane. (C) Color-coded maximum intensity projection of the ER network observed over 25 seconds with a volumetric imaging rate of ∼2.3 volumes/second with an axial sampling of Δz of 264 nm without z-stepping. (D) Three different regions of interest at three different focal planes show the remodeling of ER tubules and sheets at different depths. Scale bars in (A) and (C) 4 µm and in (B) and (D) 1 µm. FOV for (A) 25 × 25 × 1.8 µm^3^ and for (C) 30 × 30 × 1.8 µm^3^, in all images the xy-plane is shown.

To demonstrate the high volumetric imaging speed of the MF-SIM setup, we made recordings of the microtubule cytoskeleton and the ER network over extended periods of time in living cells (Fig. 3). Both the microtubule cytoskeleton and the ER network are highly dynamic intracellular structures.

Microtubule reorganization events happen regularly throughout the cell volume. Here, we demonstrate that it is possible to resolve these events with high spatial resolution and temporal resolution using MF-SIM (Fig. 3(A)-(B)). The added function of a piezo driven z-stage made it possible to combine fast scan in the z direction with MFM to optimize the axial sampling for spatial resolution, while still obtaining a high volumetric imaging speed compared to other microscopy techniques (Visualization 1). The raw images were acquired with an exposure time of 20 ms and the MF-SIM images with z-stepping were acquired sequentially without timer interval at a volumetric imaging rate of 0.9 Hz for up to 30 seconds with a laser power density of ∼38 W/cm^2^. Raw and processed data is available in Dataset 3, Ref. [40].

Like the microtubule cytoskeleton, the ER network exhibits constant remodeling. Figure 3(D) shows the dynamics of the ER network at multiple focal planes registered simultaneously. Here, the MF-SIM was operated at the highest possible speed while still acquiring sufficient signal to noise ratio for a SIM reconstruction with HiFi-SIM (Visualization 2) with this sample with a raw image exposure time of 20 ms and a volumetric imaging rate of 2.3 MF-SIM volumes/second for 25 seconds with a laser power density of ∼24 W/cm^2^. Due to the improved lateral resolution in the reconstructed images, remodeling of ER at the base of the cell can be clearly observed (Fig. 3(D) z-plane 2) [20]. Thanks to the MFM we can simultaneously see remodeling also at other focal planes (Fig. 3(D) z-plane 4 and z-plane 6). In z-plane 4 for example, it appears that round structures, which might be vesicles or lysosomes/endosomes, are embedded in and move along the ER. Raw data is available in Dataset 4, Ref. [41], processed data is available in Dataset 5, Ref. [42].

## 3. Discussion

We have here demonstrated Multi-focus Structured Illumination microscopy (MF-SIM) on living specimens. The high volumetric acquisition speed and high spatial resolution was achieved by the combination of a fast SIM setup based on optimized and synchronized components with a 7-plane MFM emission setup. This allows for the analysis of dynamic cellular organelles at multiple z-positions simultaneously with lateral subdiffractive resolution. Furthermore, the MF-SIM setup is multi-functional as it allows for regular multifocus widefield microscopy at even higher speeds but at a lower resolution and MF-SIM with extended focus stepping for higher axial sampling or to cover a larger image volume. We have applied MF-SIM imaging to the microtubule cytoskeleton that covers the entire cell volume and could resolve single microtubules at high speed. Furthermore, we visualized the ER network at different depths simultaneously and showed the reorganization of the network over time.

The present configuration of the MF-SIM setup gives an image volume of 30 × 30 × 1.8 µm with 264 nm axial sampling. Although a 7 plane MFG yields less focal planes as compared to a 9 plane or 25 plane MFG, each plane receives more emission light for a 7 plane MFG which is crucial for fast acquisition rates with short exposure times. Furthermore, we found that the total distance covered by the 7 plane MFG of 1.8 µm is suitable for common eukaryotic cells such as COS-7 cells used in this study. By using a 7-plane MFG, we therefore compromise between axial sampling, total axial distance, and light efficiency.

Technically, the volume imaging speed is only limited by the readout speed of the camera. With 1 ms exposure time and approximately 8.5 ms readout, the MF-SIM acquires the super-resolution image volume at 7.0 Hz. In comparison, a state-of-the-art commercial SIM (Zeiss Elyra 7) can acquire the same volume at just under 2 Hz at its technical limit. Even when using longer exposure times (Fig. 3, the exposure time for a raw image was set to 20 ms), MF-SIM is faster than such commercial setups. Our setup proves to be useful where the dynamics of subcellular organelles is more important than axial resolution extension, which was not implemented in this current setup as the SIM grating cannot be translated axially.

Compared to conventional 3D-SIM [5], where the sample is translated through a fixed 3D-SIM grating, the sample is fixed during the acquisition of a volume with MF-SIM. Therefore, the classical 3D-SIM reconstruction that extends the resolution axially, is not applicable for MF-SIM. However, axial contrast is improved by rejecting out-of-focus contributions using the concept of optical sectioning SIM (OS-SIM) [22,23] by compromising lateral resolution extension. Furthermore, single-plane 3D-SIM reconstruction was shown to yield similar results to classical 3D-SIM reconstruction using HiFi-SIM [16]. Therefore, we imaged the microtubule network with improved axial sampling to mimic a typical 3D-SIM acquisition while compromising speed to demonstrate the optical sectioning capabilities of MF-SIM together with HiFi-SIM. The possibility to image with different axial sampling shows the flexibility of the MF-SIM for different biological applications.

In OS-SIM, 3-beam SIM is superior to 2-beam SIM in terms of axial contrast enhancement [24,25] while being slower as 15 raw images are acquired instead of 9 raw images. We validated this by imaging a biological sample and showed improved axial contrast in 3-beam MF-SIM vs 2-beam MF-SIM (Supplement 1) and reasoned that for MF-SIM it is more appropriate to use three interfering SIM beams for all experiments due to the 3D imaging capabilities of the microscope.

In theory, axial resolution extension is possible with MF-SIM by translating the SLM along the optical axis during the acquisition. This may be impractical or unfeasible to do at the necessary speed and precision for MF-SIM imaging. Another approach to improve axial resolution in MF-SIM is with deep learning, as has been applied to 2D-SIM [26] and 3D-SIM [27]. Furthermore, supervised [28] and unsupervised deep learning denoising techniques [29] may further improve MF-SIM image quality, both before and after reconstruction. With higher time-resolution, biological samples need to be exposed to higher light intensities to generate sufficient SNR which in turn leads to phototoxicity and bleaching. Denoising may allow high speed imaging with lower light exposure while still retaining structural details in the sample.

In the current implementation, MF-SIM supports a single emission wavelength, but could be extended with more color channels each requiring its own emission path including MFM optics and camera for precise focal plane matching for each wavelength similar to the “precise color MFM” in [15]. This would allow more extensive biological studies of fast interacting or colocalizing structures.

In its current state, MF-SIM shows, compared to similar SIM modalities, an improved volumetric imaging speed at a preserved high resolution. It was here used to observe the remodeling of the microtubule and ER network.

## 4. Methods

### 4.1 Multi-focus structured illumination microscope

Conceptually, our Multi-Focus Structured Illumination Microscope (MF-SIM) presented here is similar to a previous proof-of-principle setup [14] that was built onto a commercial SIM (Zeiss Elyra PS1). However, we have improved the acquisition speed by using a spatial light modulator (SLM) with fast pattern switching speeds and synchronization of the acquisition with a microcontroller to facilitate live-sample imaging (Fig. 1).

On the excitation side of the MF-SIM, we used a 200 mW 491 nm continuous wave laser (Cobolt Calypso) digitally modulated with an AOTF (AOTFnC-VIS, AA Optoelectronic) to turn off the illumination between exposures and while the camera was reading out. The beam was de-speckled with a polarization maintaining single-mode fiber (P3-488PM-FC-5, Thorlabs) and displayed onto a phase-only nematic SLM (Meadowlark Optics, 1920 × 1152 XY Phase Series). The correct linear polarization of the incoming light to the SLM is crucial and was therefore aligned with a half-wave plate prior to the fiber.

The individual pixels on the SLM have a variable electro-optic response to voltage which results in a phase shift of the incident light. The pixels can be set to 256 discrete voltage states based on an input 8-bit pattern file. In comparison to previously used binary phase gratings [5,6], our use of a sinusoidal phase grating avoids producing spurious high frequency orders in the Fourier plane. The grating patterns are oriented at three distinct angles separated by 60° for an appropriate OTF overlap [4].

We chose the pattern orientations 41°, 101° and 161°, which allowed us to use a non-integer pattern period value of 10.5 pixels. The pattern period determines the spacing between the ± 1 order and the 0 order at the pupil of the objective lens and the theoretical SIM resolution improvement. In the present setup, the resolution is only extended laterally. However, optical sectioning is still retained if the shifted copies of the optical transfer function overlap each other while sacrificing lateral resolution improvement [23]. Therefore, we chose a lower frequency pattern period of 10.5 pixels on the SLM corresponding to 237 nm in the sample and a subsequent lateral resolution improvement of 1.74x.

The intensity of the ±1-order beam relative to the 0-order beam translates to how strong the 2nd and 1st order frequency components are in the Fourier transform of the raw 3-beam-SIM data. By decreasing the amplitude of the sinusoidal grating pattern displayed on the SLM we could suppress the 0th-order beam and increase the strength of the ±1-order beam and the subsequent non-zero order components in the Fourier transform to achieve the same intensity of all three interfering beams. The polarization of the SIM beams is crucial to ensure proper modulation contrast. Typically, for 2D-SIM, the beams are azimuthally linearly polarized using a segmented polarizer or half-wave plate [6]. Another way of rotating the linear polarization is to use a liquid crystal polarization rotator [30], however, those are not commercially available anymore.

At lower frequency patterns, when the aim is not to maximize the lateral resolution, the influence of polarization on the pattern contrast in the sample is less important [31]. Therefore, we chose to use a quarter wave plate to circularly polarize the SIM beams for a homogenous modulation depth at all three orientations [32].

After the SLM, the excitation beams were focused by a 400 mm lens (ACT508-400-A, Thorlabs) onto a mask where the 0th order beam could be blocked (for 2-beam mode). Then a combination of a 300 mm lens (ACT508-300-A, Thorlabs) and a 400 mm lens (ACT508-400-A, Thorlabs) projected the resulting beams at the back focal plane of a 63x/1.4 oil objective lens (420782-990-799 Zeiss).

We used a dichroic filter (ZT488/561rpc, Chroma) to separate the excitation light and the emission light. The dichroic mirror and the objective lens were mounted in a commercial microscope stand (Axio Observer, Zeiss). The sample was mounted in a 3-axis piezo stage for fast and accurate axial positioning (P-545.xR8S PInano XY(Z)-Piezosystem, Physik Instrumente) to allow for improved z-sampling if needed.

The fluorescent response from the sample was detected by the same objective lens as in the excitation and was transmitted through the dichroic mirror and a magnifying tube lens (Zeiss optovar 1.6x, Zeiss), which increased the effective magnification on the emission side by 1.6x to a total emission magnification of 63 * 1.6 = 100.8x.

The following multi-focus system is similar to previously published work [13,14]. In this setup, we have manufactured the multi-focus grating (MFG) to yield 7 distinct focal planes with a 264 nm z-spacing. Previously, MFGs with 7, 9 and 25 distinct focal planes have been used [33], however, the transmission efficiency inversely correlates with the number of focal planes a MFG produces. Furthermore, the 25 planes design is not compatible with a single camera, as the individual field of views of the distinct focal planes are too small for proper lateral sampling. Therefore, the 7 planes design was chosen due to light efficiency and sampling. With the 7-plane MFG, each plane obtains approximately 11% of the total emission intensity recorded by the objective. More in detail explanation of the light throughput of the 7-plane MFG in comparison to other MFGs can be found in Refs. [15,33]. The MFG induces chromatic dispersion aberrations as the emission light of a typical fluorophore is not monochromatic but rather a wavelength range (GFP ∼ 500–550 nm). This is corrected by the chromatic correction grating (CCG) [13]. An optical prism was mounted together with the CCG into a custom-made mount to direct the light towards a large achromatic doublet lens (f = 250 mm, PAC095AR.14, Newport) which was used to form the multi-focus image on a sCMOS camera (Orca Flash 4.0 v3, Hamamatsu).

The camera is controlled by a Windows workstation using Micro Manager 2.0 [34] set up to acquire images in external trigger mode. The camera chip was cropped to 1600 × 1600 pixels to cover the MF-SIM field of view (FOV). The AOTF, the SLM, the piezo stage and the camera were synchronized by a microcontroller (Adafruit Metro 348) which served as the master trigger. The stripe grating patterns were pre-loaded into the memory of the SLM according to the manufacturer’s guidelines. When acquiring an MF-SIM timelapse, the microcontroller sent a trigger pulse to the SLM for loading the first pattern of the sequence. After a short microsecond delay, another trigger pulse to the camera started the acquisition of one raw MF-SIM image. At the end of the exposure, the camera readout took approximately 8.5 ms during which a trigger pulse was sent to the SLM for loading the next pattern. During any SIM acquisition, it is not possible to choose a faster, continuous readout of the camera due to the rolling shutter effect which leads to different SIM pattern orientations being captured by different parts of the camera chip. Therefore, a new capture by the camera can only be started after the previous capture has been read out. With a sufficiently bright sample the acquisition speed of the MF-SIM is hence limited by the readout of all the pixels that cover the 7 different planes. For example, with an exposure time of 1 ms, the approximate (volumetric) frame rate of MF-SIM with 7 planes would result in 7.0 volumes per second. For samples where improved z-sampling is desired, axial z-stepping with the piezo stage is complemented after one sequence of 15 raw SIM images and adds approximately 170 ms of acquisition time per translation in z to the frame rate. The precise timing diagram of all components can be found in Supplement 1.

### 4.2 Computational registration and reconstruction

The raw MF-SIM data was registered and reconstructed to yield a super-resolved image volume. First, the seven different focal planes in each raw 2D image were extracted and pixelwise registered according to a registration matrix into a MF-SIM stack with the 7 Z planes, 3 pattern rotations, 5 pattern translations and t timepoints. We constructed the registration matrix by acquiring a point spread function of a 100 nm bead in multi-focus widefield mode stepping through the focus with every single multi-focus beam, computing a maximum intensity projection along the axial direction and then overlaying the cropped multi-focus images while keeping the overlaying coordinates.

The raw MF-SIM stack was then reconstructed plane by plane with HiFi-SIM [16], a reconstruction scheme that has been shown to reduce common SIM artifacts and provide improved optical sectioning over comparable single-plane 3D-SIM algorithms. By taking the average over the 15 raw images we created corresponding widefield images. To compare the results, we also deconvolved the widefield volume using Huygens v23.10, Scientific Volume Imaging. We did not use interleaved reconstruction [35], as it only increases the temporal sampling of lower ‘widefield’ spatial frequencies [36] but not higher sub-diffraction limited frequencies. After the reconstruction process, we applied sub-pixel registration on the MF-SIM stack by using pystackreg (https://github.com/glichtner/pystackreg), which is a python implementation of the Fiji plugin StackReg (http://bigwww.epfl.ch/thevenaz/stackreg/) and is based on the algorithm by Philippe Thévenaz [37]. We applied the sub-pixel registration after the reconstruction process, as it may interfere with high frequency sample-grating pattern interactions. The python code for the pixelwise and sub-pixelwise registration can be found together with the raw and reconstructed data for each figure at Optica Figshare repository in Code 1, Ref. [43], the Matlab code for the reconstruction can be found at [16].

### 4.3 Sample preparation

For acquiring a point spread function, glass coverslips (Carl Zeiss Cover Glasses, High Performance, thickness 0.17 mm ± 5 µm) were cleaned with 70% Ethanol and pretreated with poly-L-lysine (P8920-100 ML) for 15 minutes. After that, different dilutions of 100 nm fluorescent beads (FluoSpheres Carboxylate-Modified Microspheres, F8803, Invitrogen) were applied to the coverslip, unbound beads were washed away, and the coverslips were mounted onto glass slides with ProLong Gold Antifade Mountant (Invitrogen, P36930).

NIH/3T3 cells (CRL-1658, ATCC) and COS-7 cells (CRL-1651, ATCC) were cultured in Dulbecco's Modified Eagle Medium (DMEM, high glucose, with GlutaMAX Supplement,10566016, ThermoFisher) which was supplemented by 10% fetal bovine serum (FBS, 10500064, ThermoFisher) at 37°C and 5% CO2. For the experiments, cells were detached using 0.05% Trypsin-EDTA (T3924-500 ML, ThermoFisher) and ∼50.000 cells were seeded per glass coverslip and grown until ∼40% confluency before transfecting with 1 µg/coverslip of the plasmids EMTB-3XGFP [21] (#26741, Addgene) or pcDNA3/er-(n2)oxStayGold(c4) [18] (#185822, Addgene) and 3 µl of TransIT-LT1 transfection agent (Mirus) for 24–48 h. For fixation, the media of the cells was replaced with 4% paraformaldehyde prewarmed to 37°C for 10 minutes at room temperature. After that, the cells were washed with phosphate buffered saline (PBS) three times and mounted as described for the bead samples. For live-cell imaging, the coverslips with the cells were washed with PBS and transferred to a 35 mm magnetic chamber (Chamlide CMB, Gataca Systems) and imaged with DMEM without phenol red.

## Data Availability

Data underlying the results presented in this paper are available in Dataset 1, Ref. [38] Dataset 2, Ref. [39] Dataset 3, Ref. [40] Dataset 4, Ref. [41] and Dataset 5, Ref. [42] and in Code 1, Ref. [43].

## Supplemental document

See Supplement 1 for supporting content.
