# Supplementary material for: Fast volumetric multifocus structured illumination microscopy of subcellular dynamics in living cells
Source: Biomed Opt Express. 2024 Mar 11;15(4):2281–92. doi: 10.1364/BOE.516261 (PMC11019691; doi:10.1364/BOE.516261)
Supplement: Supplementary file 1 [file boe-15-4-2281-s001.pdf]

Supplemental Document

Biomedical Optics EXPRESS

Fast volumetric multifocus structured illumination microscopy of subcellular dynamics in living cells: supplement

MAXIMILIAN LUKAS SENFTLEBEN,[1,*] ANTONE BAJOR,[2] EDUARDO HIRATA,[1] SARA ABRAHAMSSON,[2] AND HJALMAR BRISMAR[1]

[1] *Department of Applied Physics, KTH Royal Institute of Technology, Science for Life Laboratory, Stockholm, Sweden*
[2] *Baskin School of Engineering, University of California Santa Cruz, 1156 High Street, Santa Cruz, 95064, CA, USA*
[*] *brismar@kth.se*

This supplement published with Optica Publishing Group on 11 March 2024 by The Authors under the terms of the Creative Commons Attribution 4.0 License in the format provided by the authors and unedited. Further distribution of this work must maintain attribution to the author(s) and the published article's title, journal citation, and DOI.

Supplement DOI: https://doi.org/10.6084/m9.figshare.25343035

Parent Article DOI: https://doi.org/10.1364/BOE.516261

FAST VOLUMETRIC MULTIFOCUS STRUCTURED ILLUMINATION MICROSCOPY OF SUBCELLULAR DYNAMICS IN LIVING CELLS: SUPPLEMENTAL DOCUMENT

Supplementary figure 1

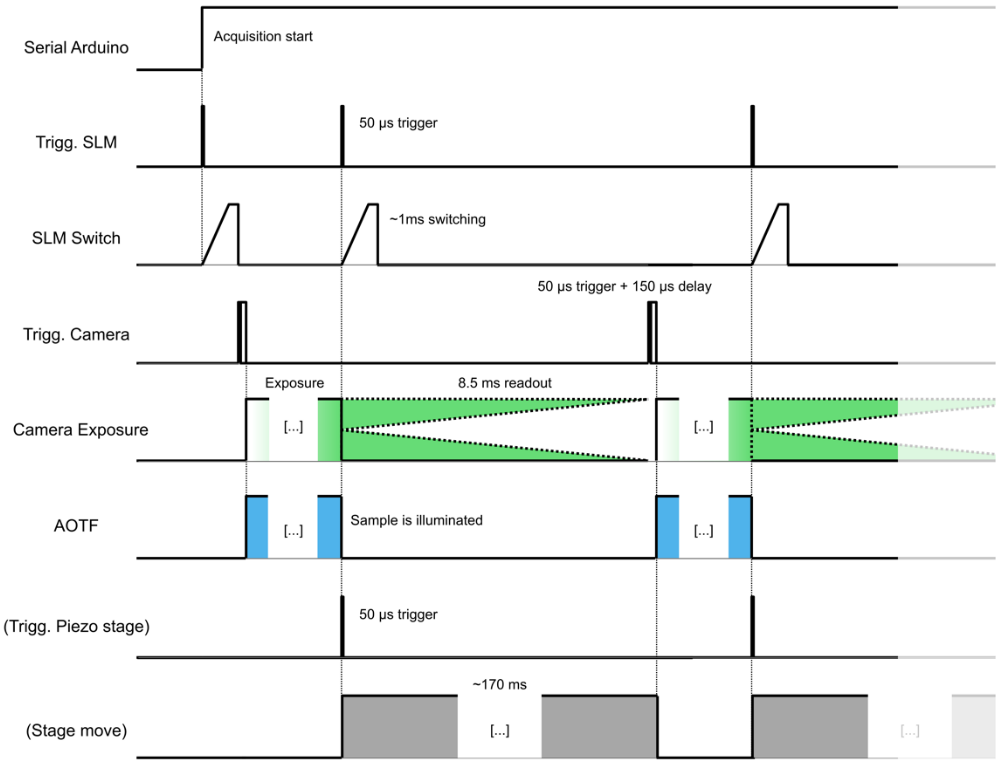

Supplementary Figure 1: Timing diagram of the MF-SIM. The acquisition is started by a Serial input to the Arduino from the computer. Then, a trigger is sent to the SLM via the Arduino and the SLM loads the first grating pattern within approximately 1 ms. Then, the Arduino triggers the camera, which after a short delay of 150 µs starts the acquisition of the first frame. Similarly, the AOTF is triggered to allow the laser to illuminate the sample as long as the camera is exposing. The Arduino reads the camera exposure pin and once the exposure is over the camera switches to reading out the cropped field of view on the camera chip (1600 lines), which takes approximately 8.5 ms. During this time, the Arduino sends a trigger to the SLM for loading the next grating pattern. If z-stepping is required, a trigger is sent to the piezo stage which starts moving to the next axial position. Importantly, the next exposure only begins once the stage movement is complete.

Supplementary figure 2

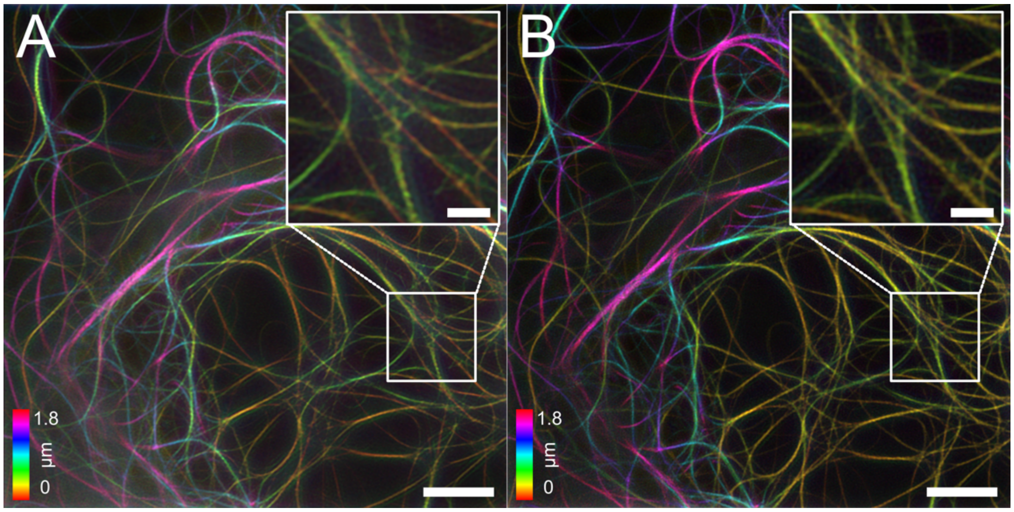

Supplementary Figure 2: Comparison of 2-beam MF-SIM (A) and 3-beam MF-SIM (B). Depth color-coded maximum intensity projection shows out-of-focus blur in 2-beam MF-SIM image, which is reduced in the 3-beam MF-SIM image. Scale bar 4 µm, 1 µm in the inset.

Supplementary video 1

MAP7-GFP in COS7-cells show microtubule reorganization at 1.2 volumes per second. Video at 7 fps.

Supplementary video 2

StayGold-KDEL in COS7-cells show endoplasmatic reticulum dynamics imaged at 2.8 volumes per second. Video at 7 fps.
