# Supplementary figures and images for: Fast volumetric multifocus structured illumination microscopy of subcellular dynamics in living cells
Source: Biomed Opt Express. 2024 Mar 11;15(4):2281–92. doi: 10.1364/BOE.516261 (PMC11019691; doi:10.1364/BOE.516261)

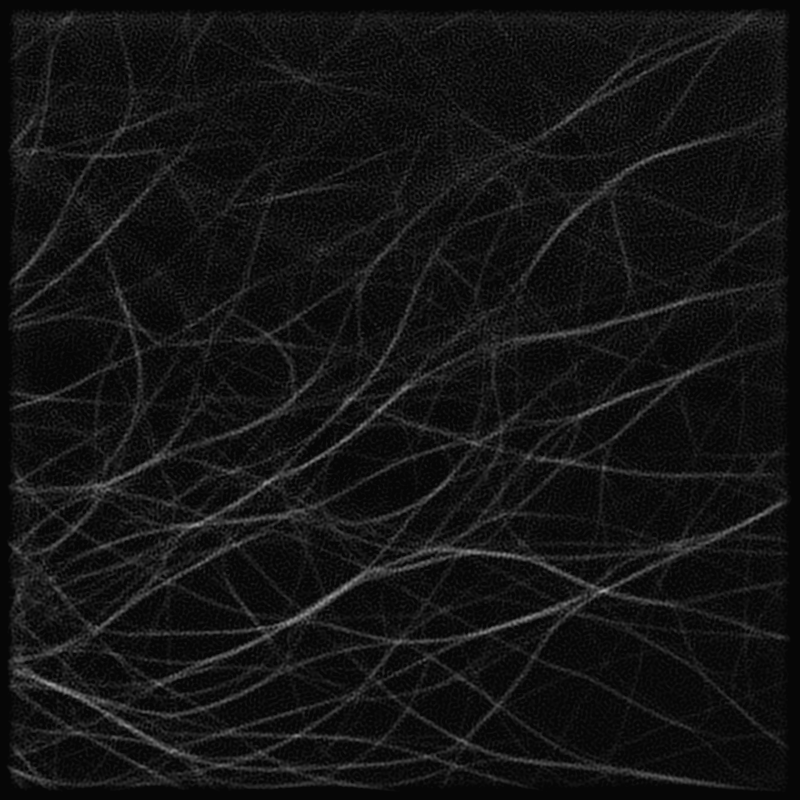

Supplement: Supplementary file 2 [file boe-15-4-2281-d001.zip › fig1/Tubulin_image/COS7_16_2_t0_recon_reg.tif]

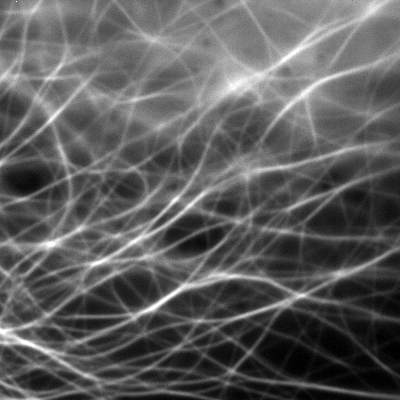

Supplement: Supplementary file 2 [file boe-15-4-2281-d001.zip › fig1/Tubulin_image/COS7_16_2_t0_wf.tif]

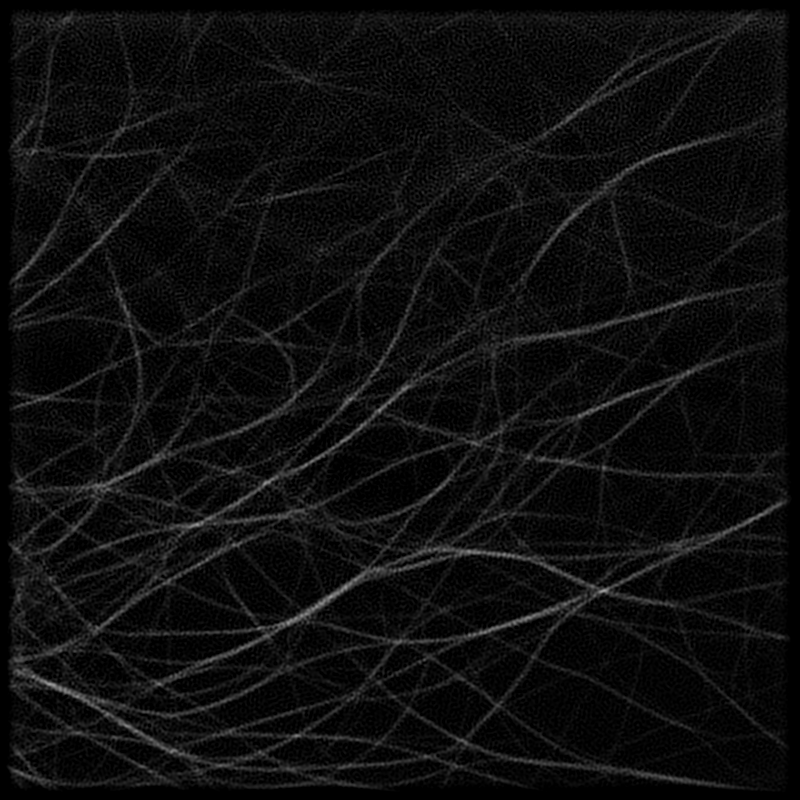

Supplement: Supplementary file 2 [file boe-15-4-2281-d001.zip › fig1/Tubulin_image/COS7_16_2_t0_recon.tif]

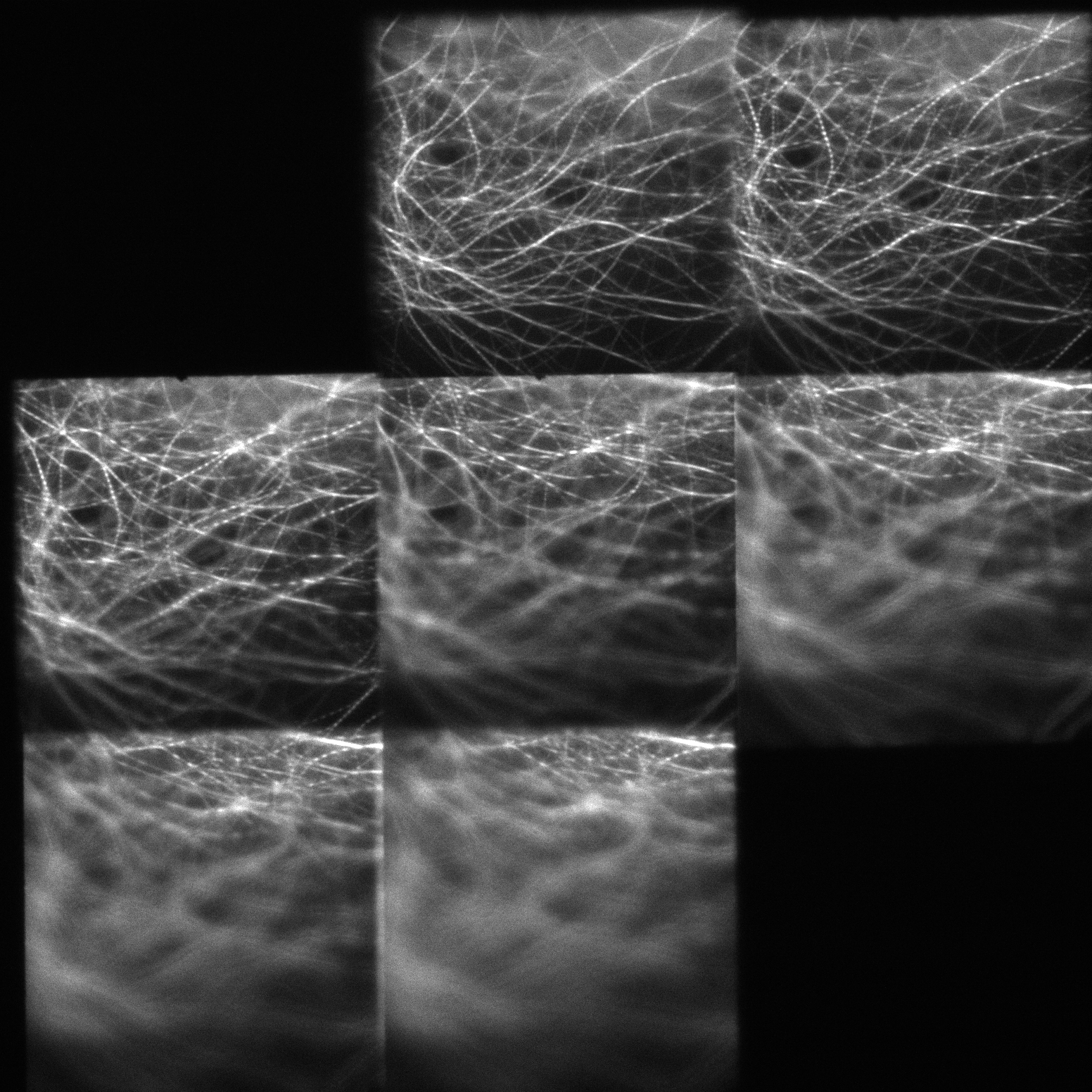

Supplement: Supplementary file 2 [file boe-15-4-2281-d001.zip › fig1/Tubulin_image/raw_data/img_channel000_position000_time000000005_z000.tif]

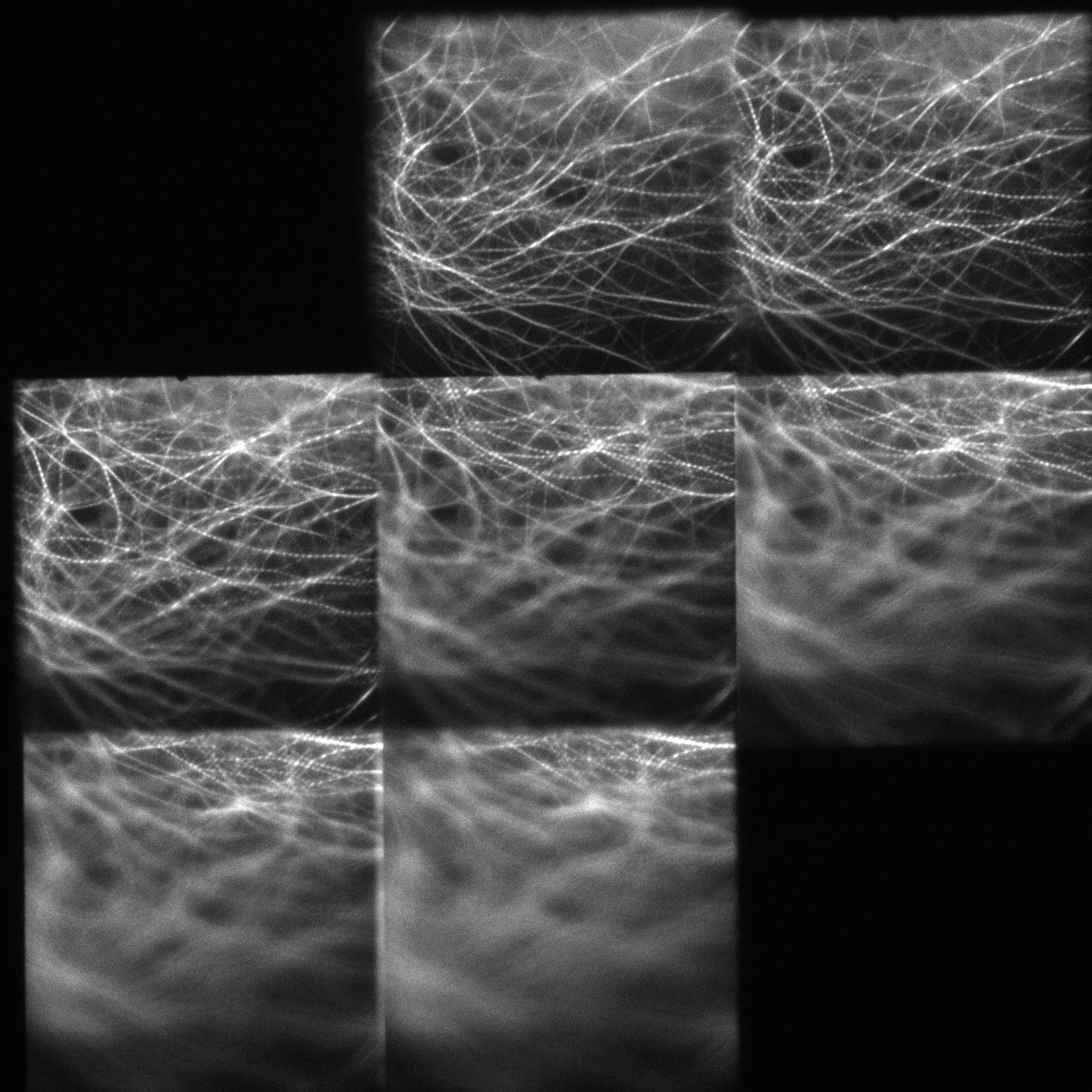

Supplement: Supplementary file 2 [file boe-15-4-2281-d001.zip › fig1/Tubulin_image/raw_data/img_channel000_position000_time000000004_z000.tif]

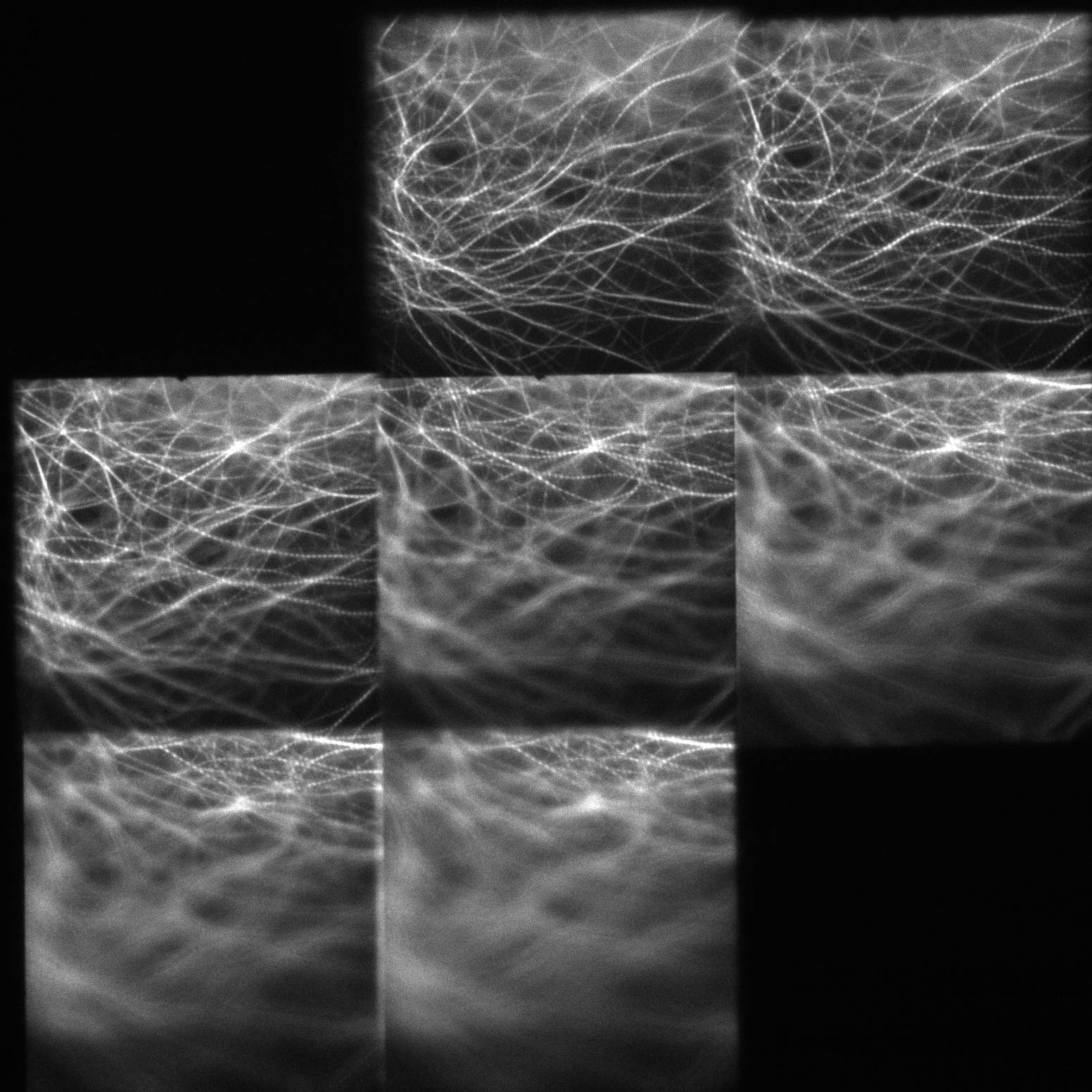

Supplement: Supplementary file 2 [file boe-15-4-2281-d001.zip › fig1/Tubulin_image/raw_data/img_channel000_position000_time000000012_z000.tif]

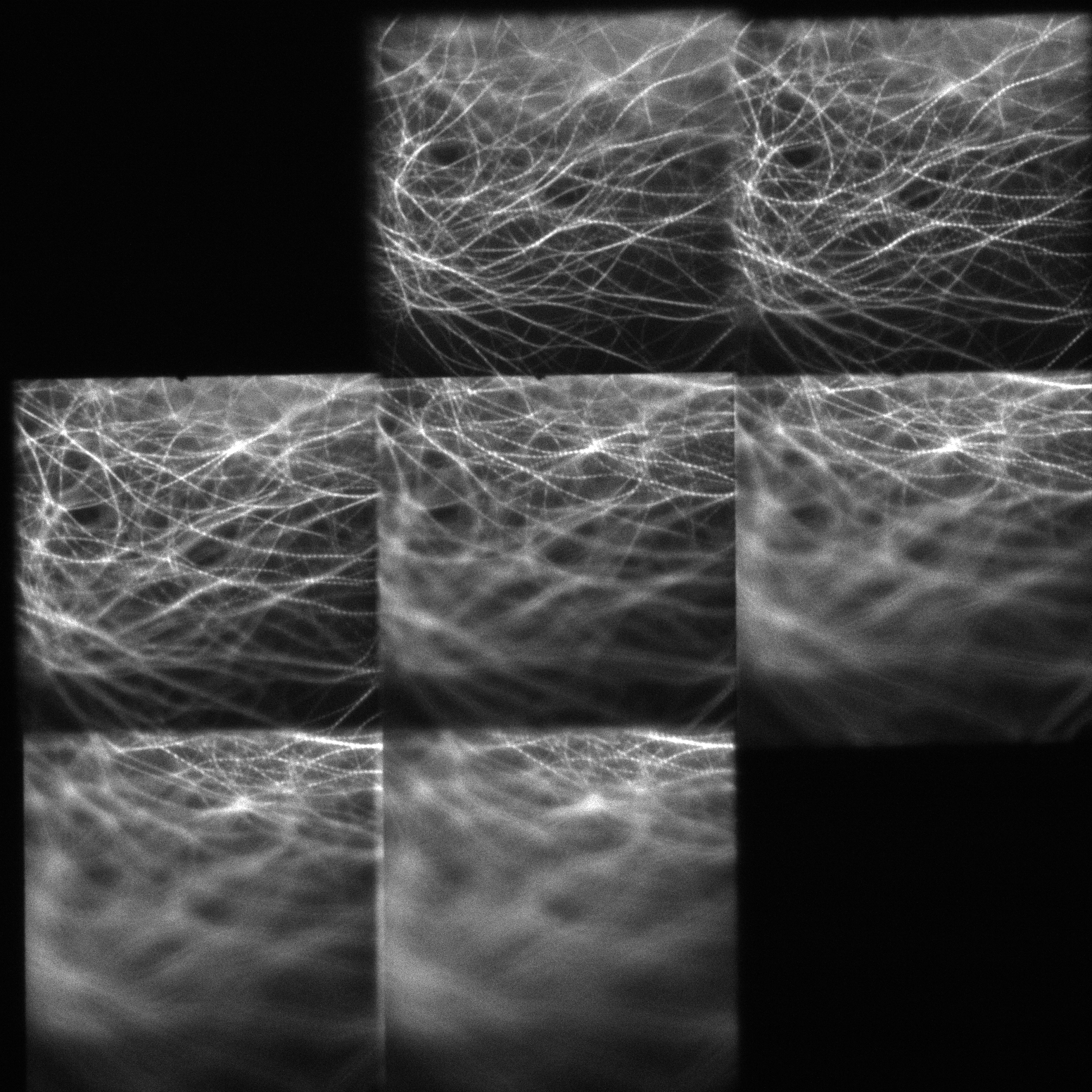

Supplement: Supplementary file 2 [file boe-15-4-2281-d001.zip › fig1/Tubulin_image/raw_data/img_channel000_position000_time000000013_z000.tif]

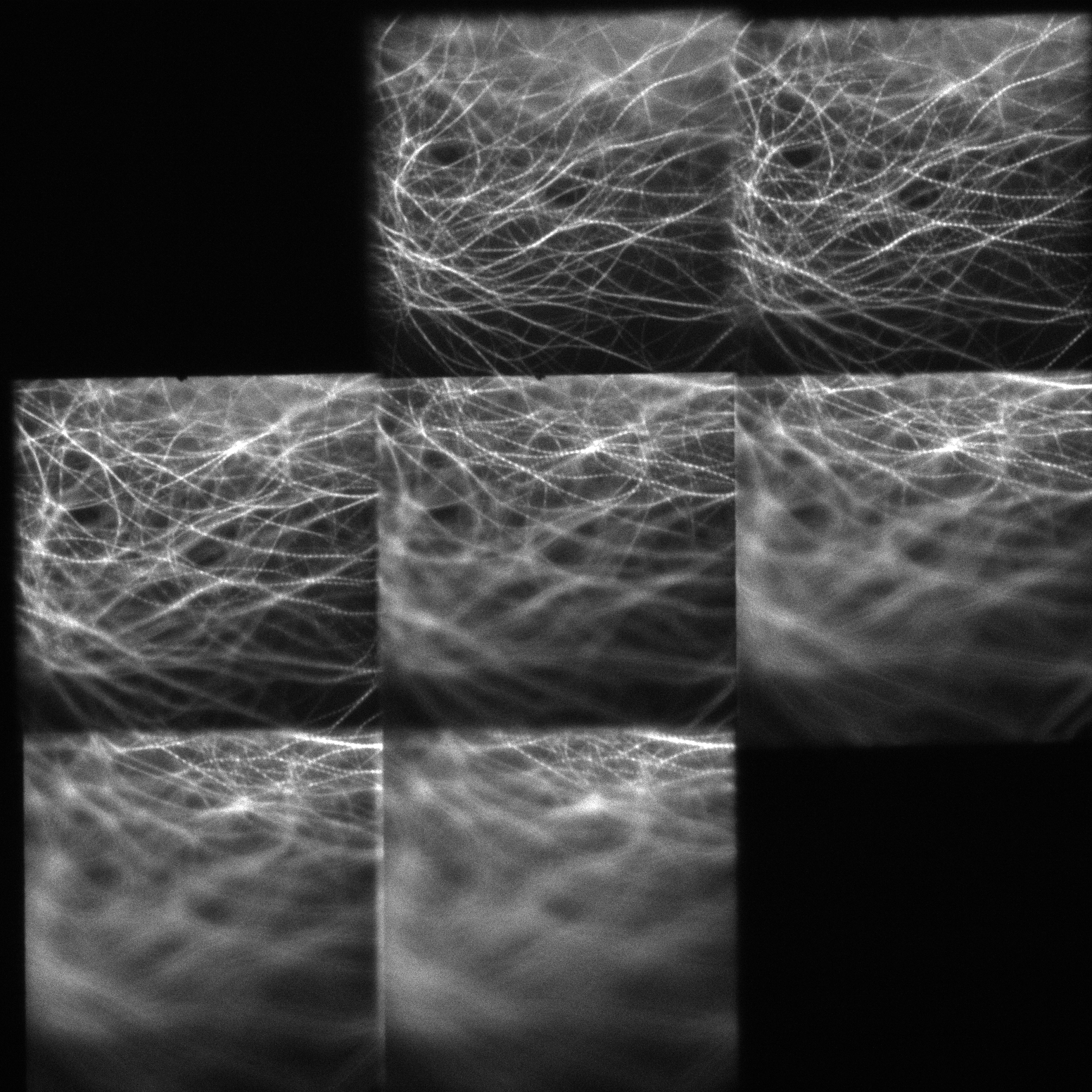

Supplement: Supplementary file 2 [file boe-15-4-2281-d001.zip › fig1/Tubulin_image/raw_data/img_channel000_position000_time000000014_z000.tif]

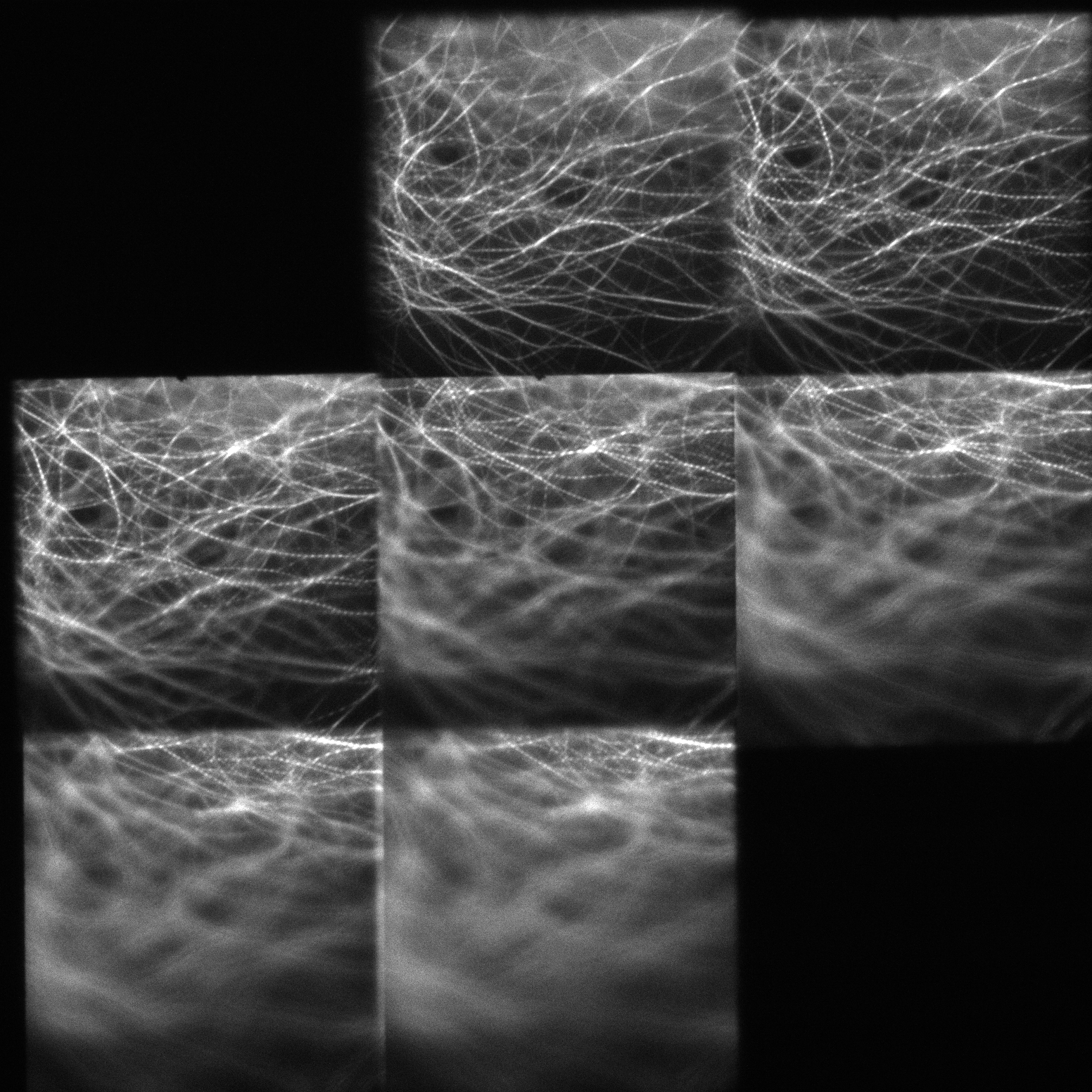

Supplement: Supplementary file 2 [file boe-15-4-2281-d001.zip › fig1/Tubulin_image/raw_data/img_channel000_position000_time000000002_z000.tif]

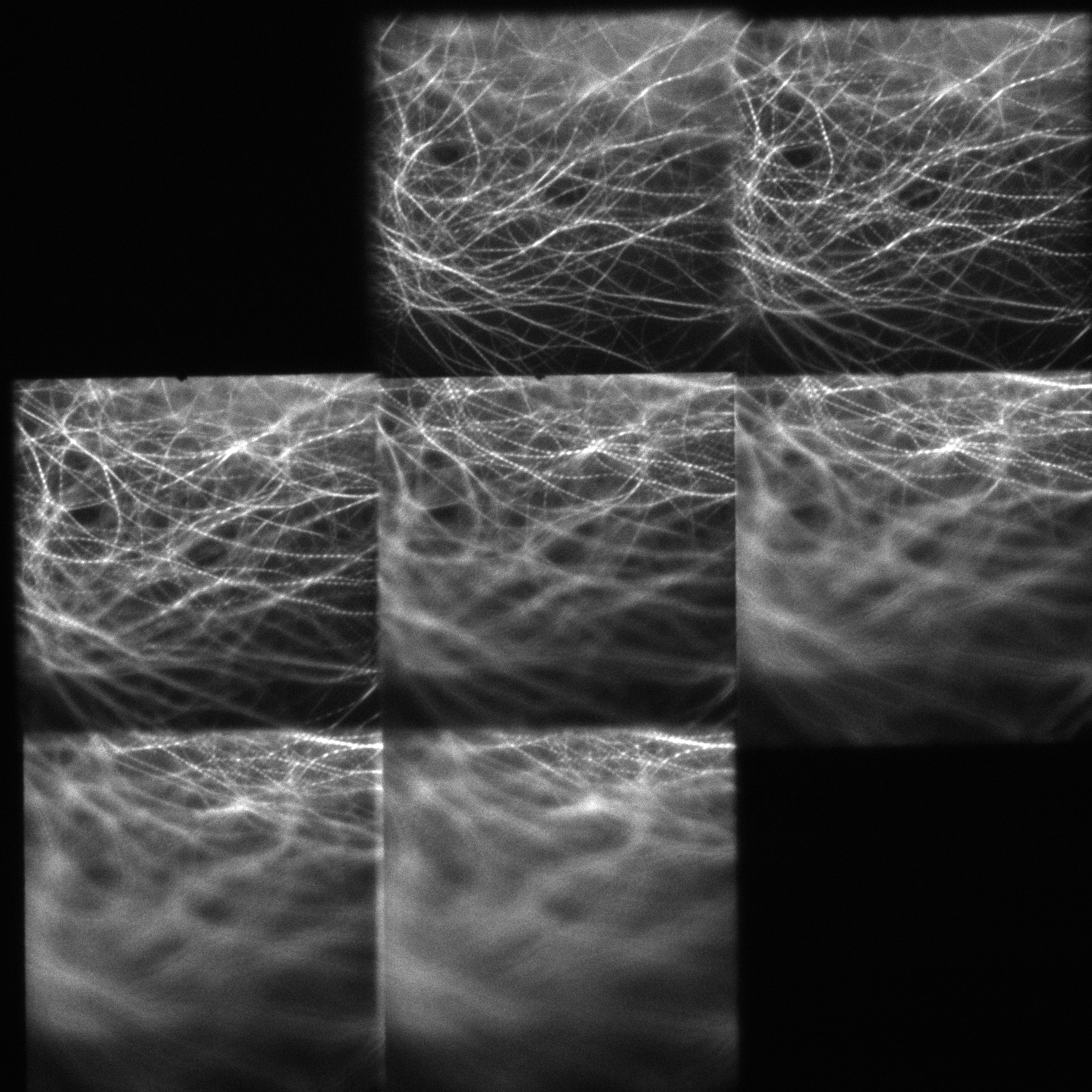

Supplement: Supplementary file 2 [file boe-15-4-2281-d001.zip › fig1/Tubulin_image/raw_data/img_channel000_position000_time000000003_z000.tif]

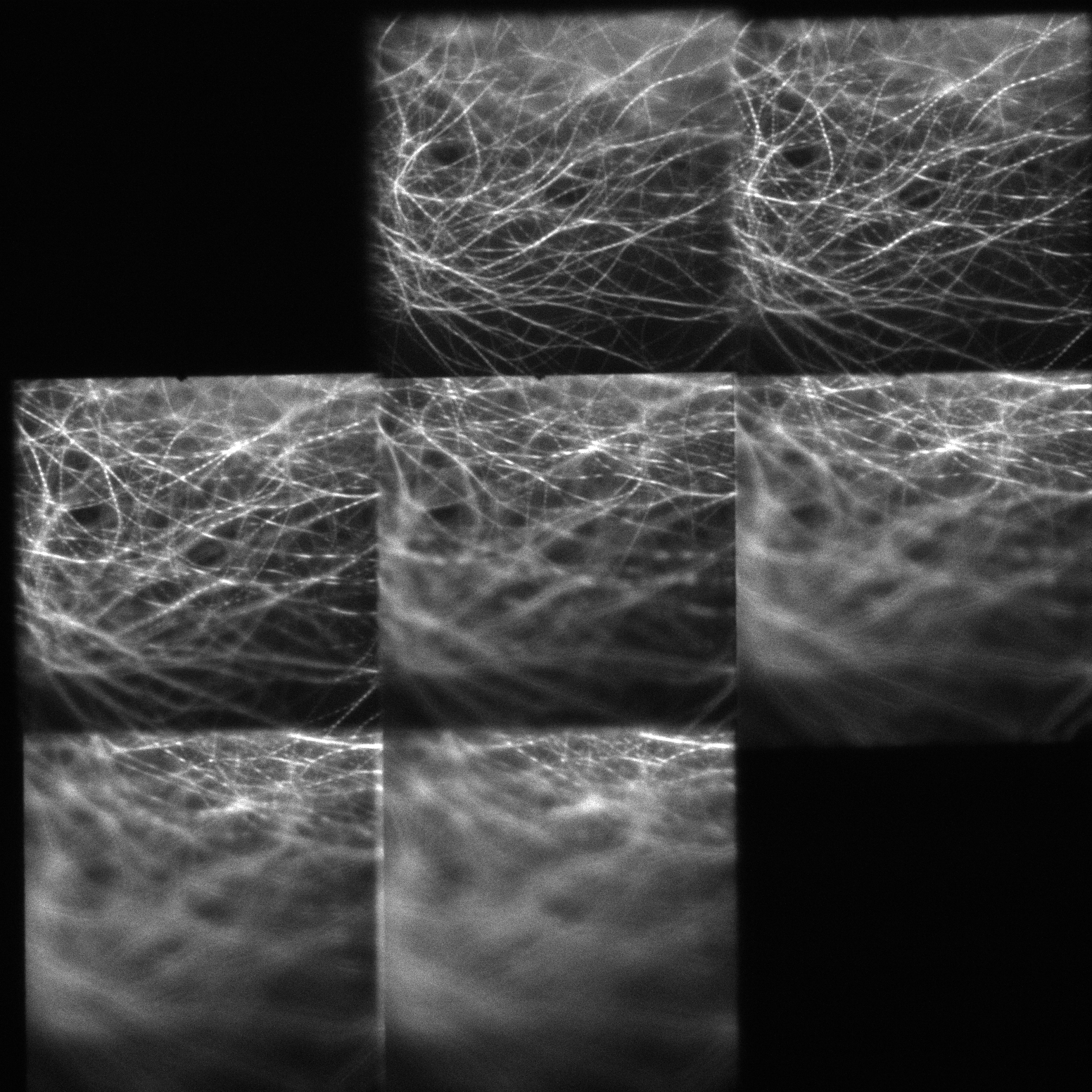

Supplement: Supplementary file 2 [file boe-15-4-2281-d001.zip › fig1/Tubulin_image/raw_data/img_channel000_position000_time000000008_z000.tif]

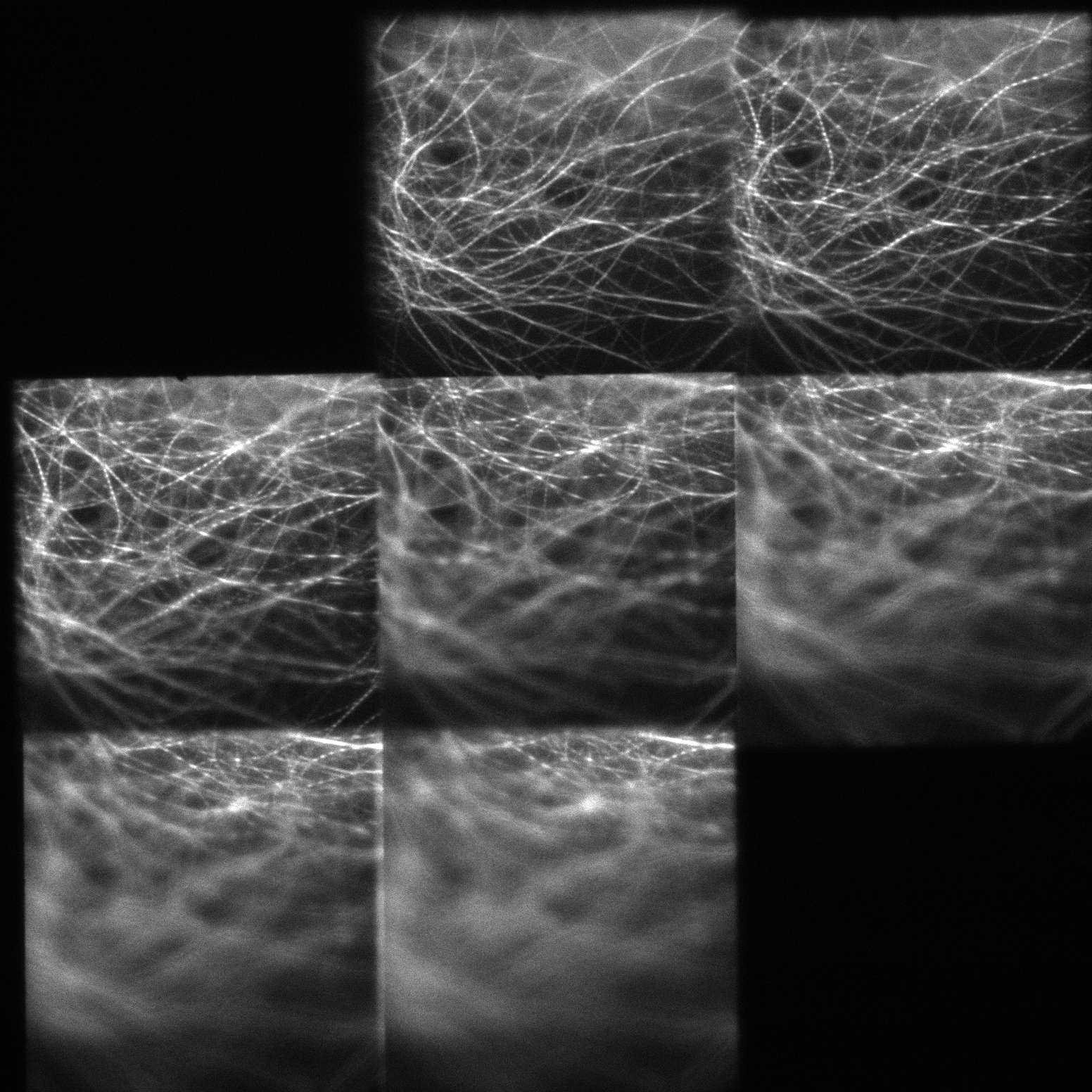

Supplement: Supplementary file 2 [file boe-15-4-2281-d001.zip › fig1/Tubulin_image/raw_data/img_channel000_position000_time000000009_z000.tif]

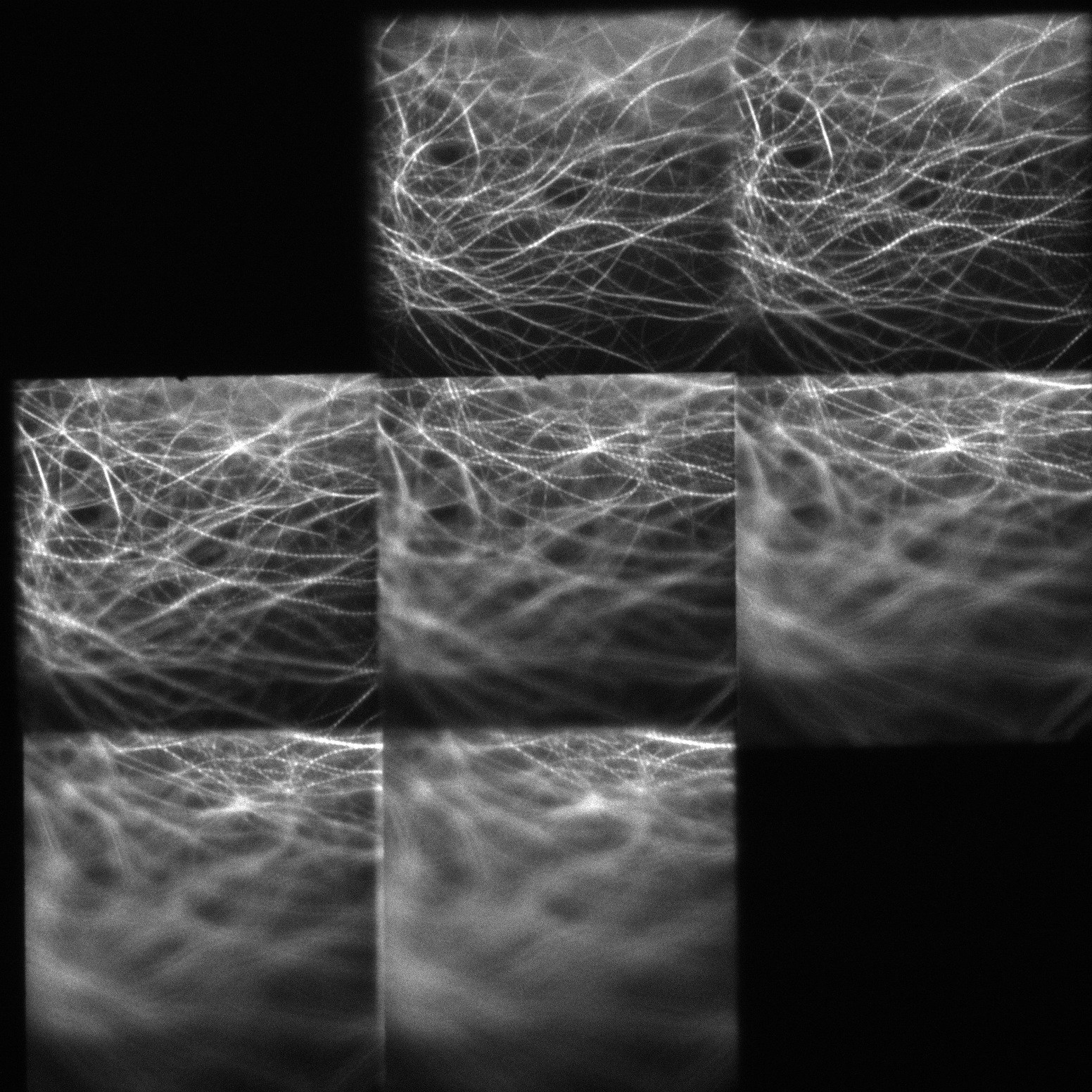

Supplement: Supplementary file 2 [file boe-15-4-2281-d001.zip › fig1/Tubulin_image/raw_data/img_channel000_position000_time000000011_z000.tif]

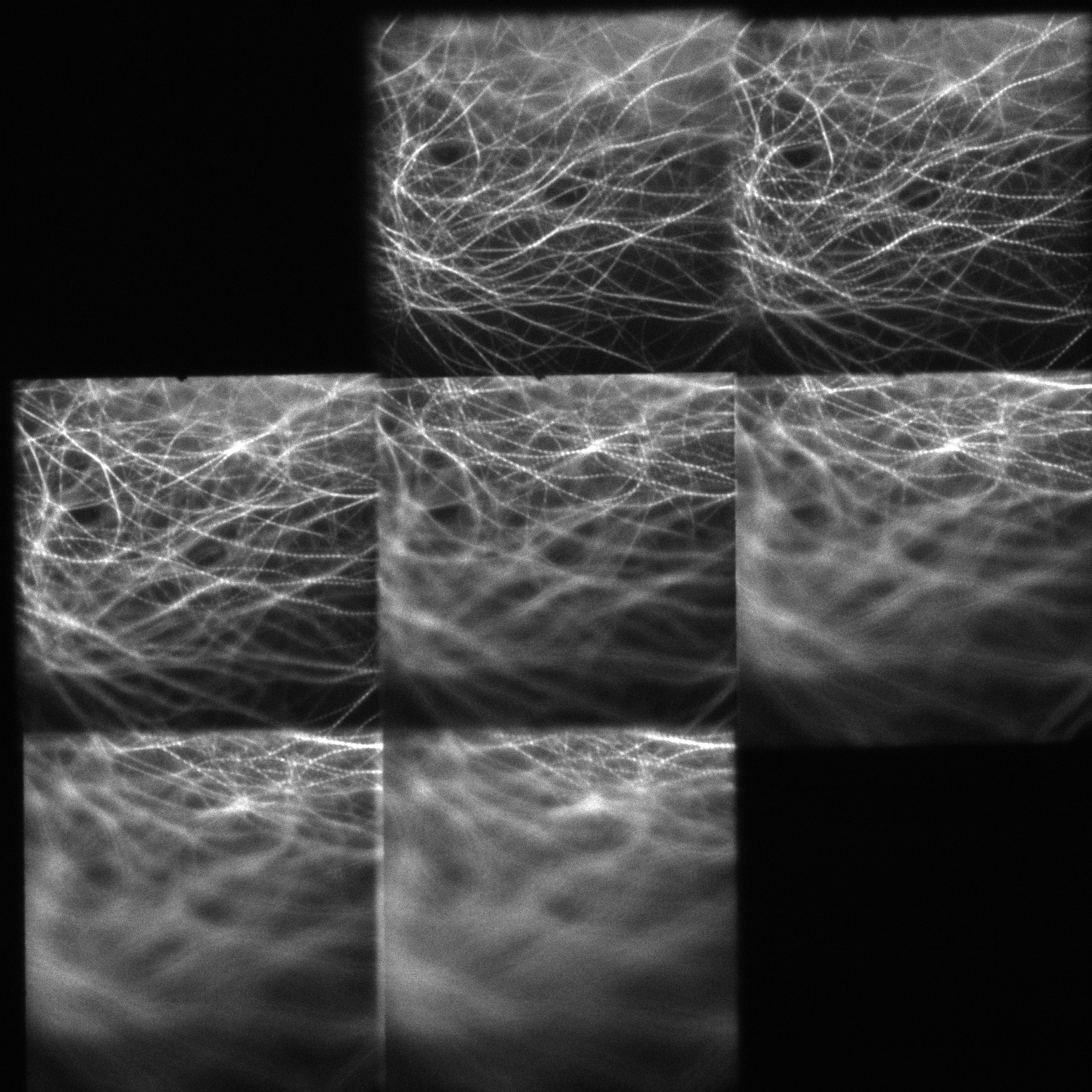

Supplement: Supplementary file 2 [file boe-15-4-2281-d001.zip › fig1/Tubulin_image/raw_data/img_channel000_position000_time000000010_z000.tif]

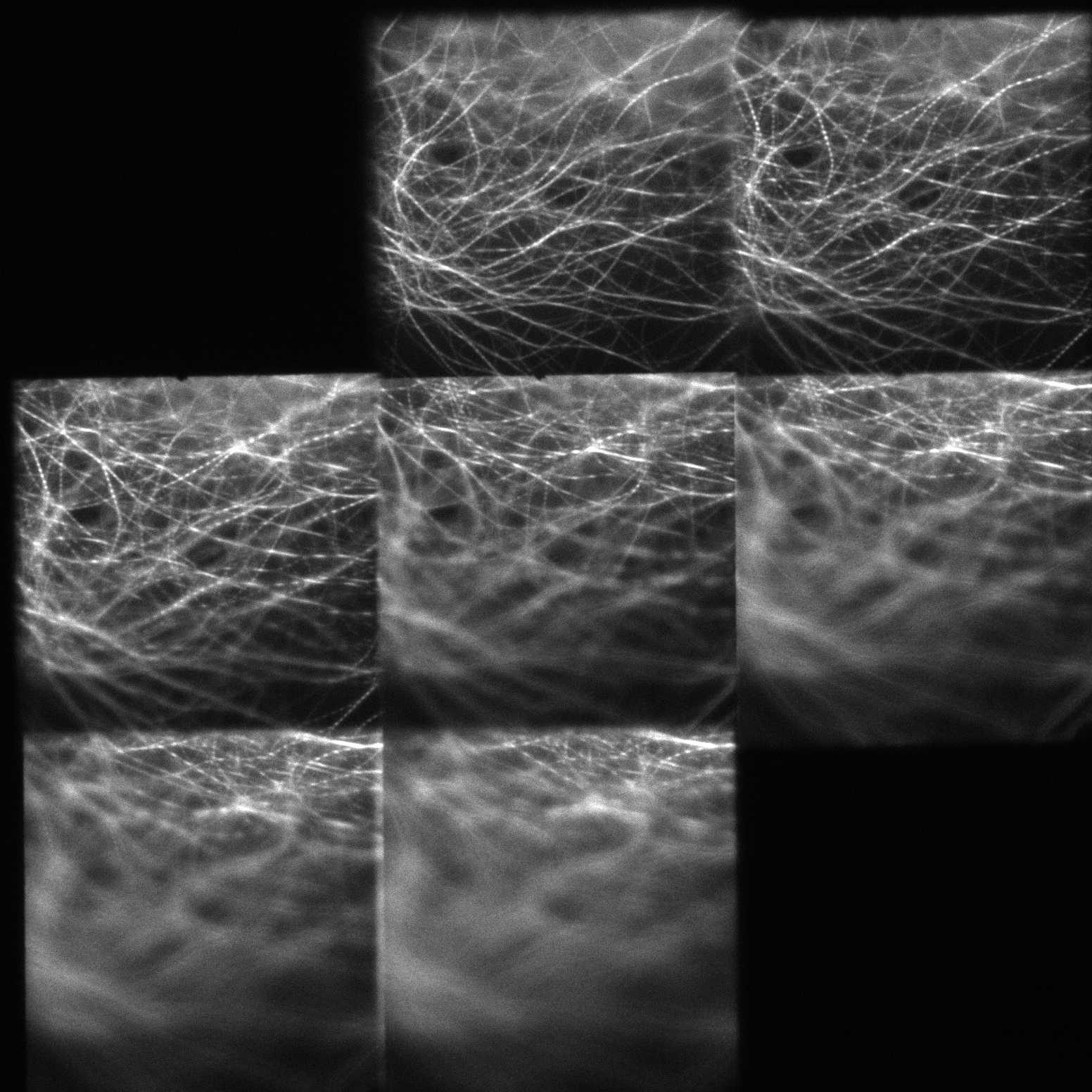

Supplement: Supplementary file 2 [file boe-15-4-2281-d001.zip › fig1/Tubulin_image/raw_data/img_channel000_position000_time000000006_z000.tif]

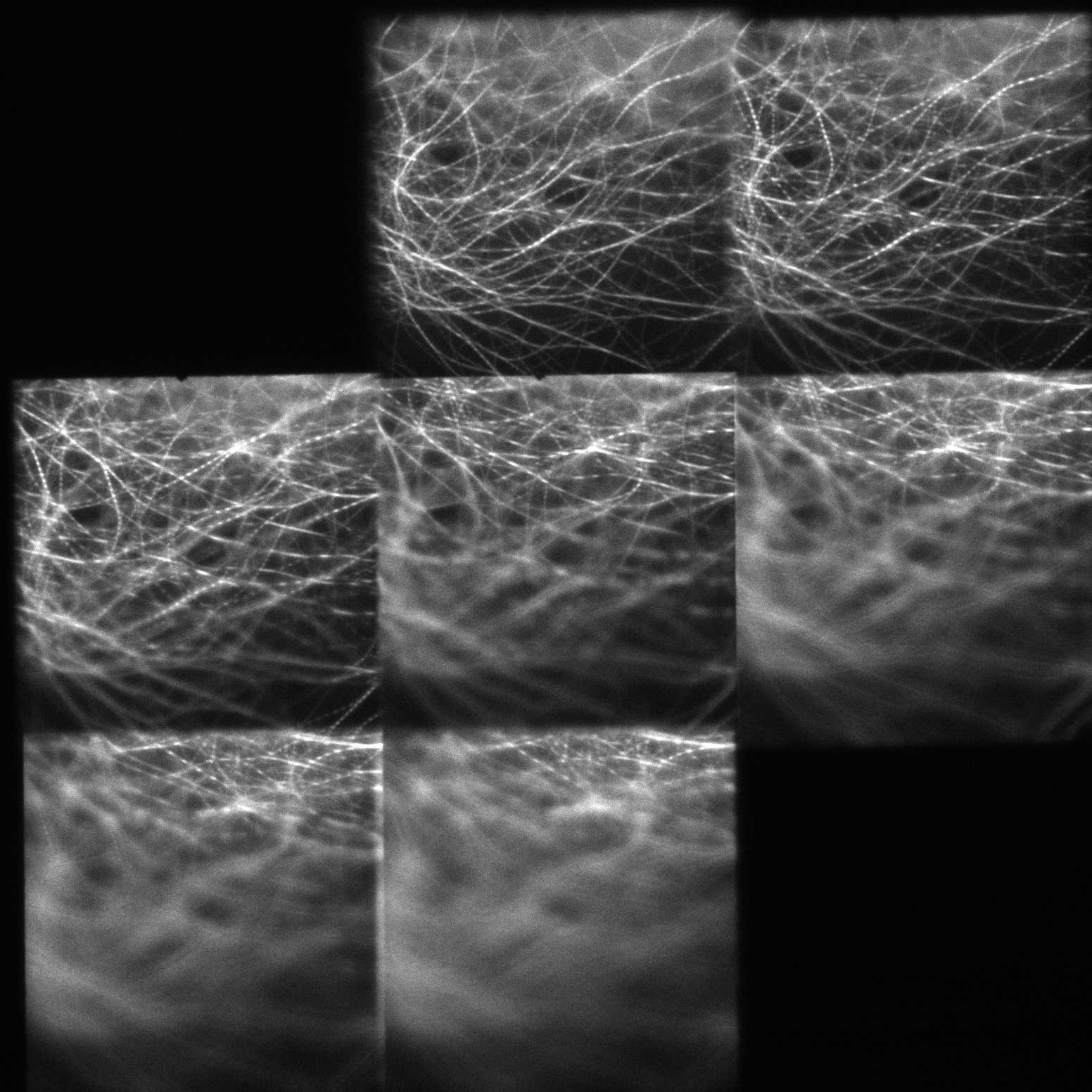

Supplement: Supplementary file 2 [file boe-15-4-2281-d001.zip › fig1/Tubulin_image/raw_data/img_channel000_position000_time000000007_z000.tif]

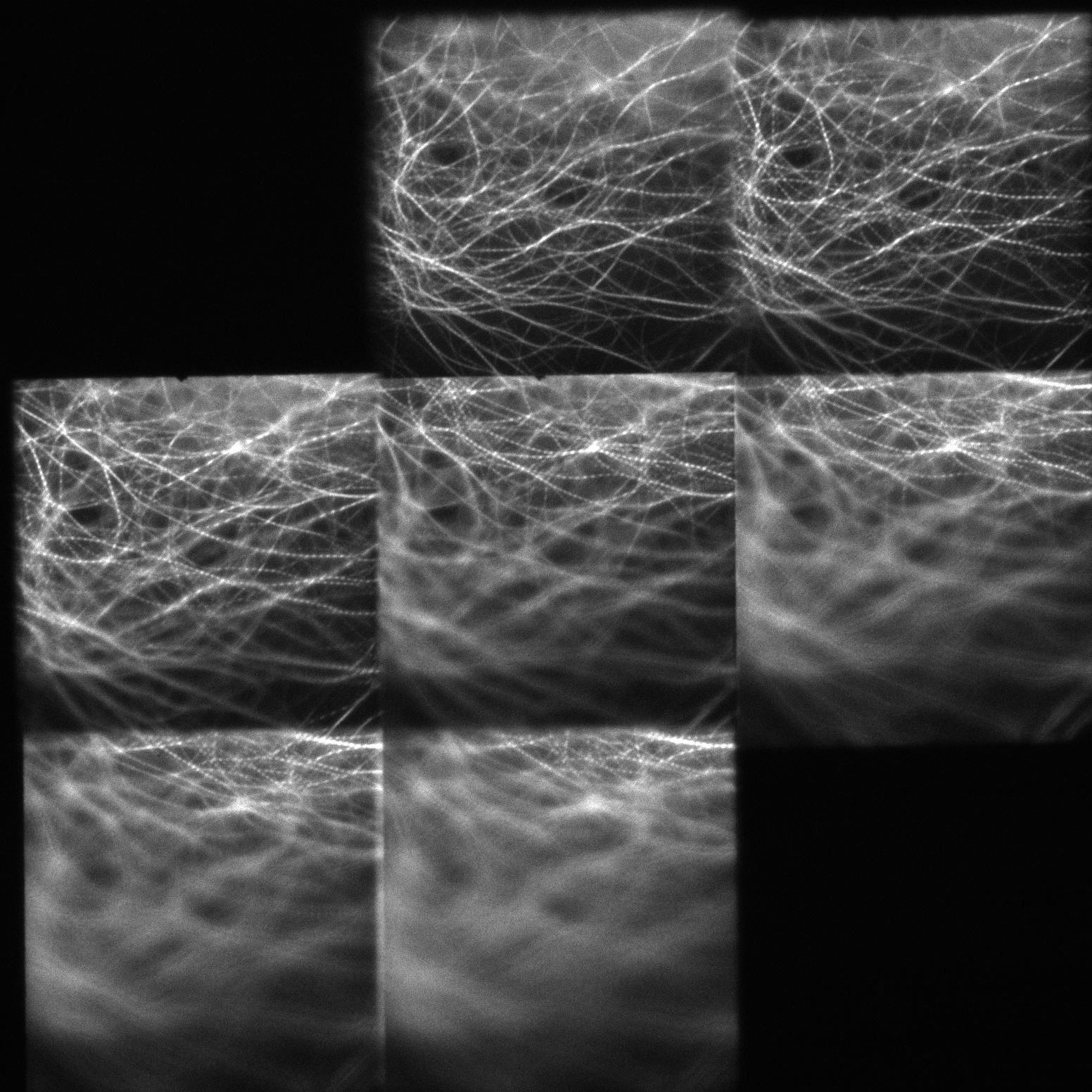

Supplement: Supplementary file 2 [file boe-15-4-2281-d001.zip › fig1/Tubulin_image/raw_data/img_channel000_position000_time000000001_z000.tif]

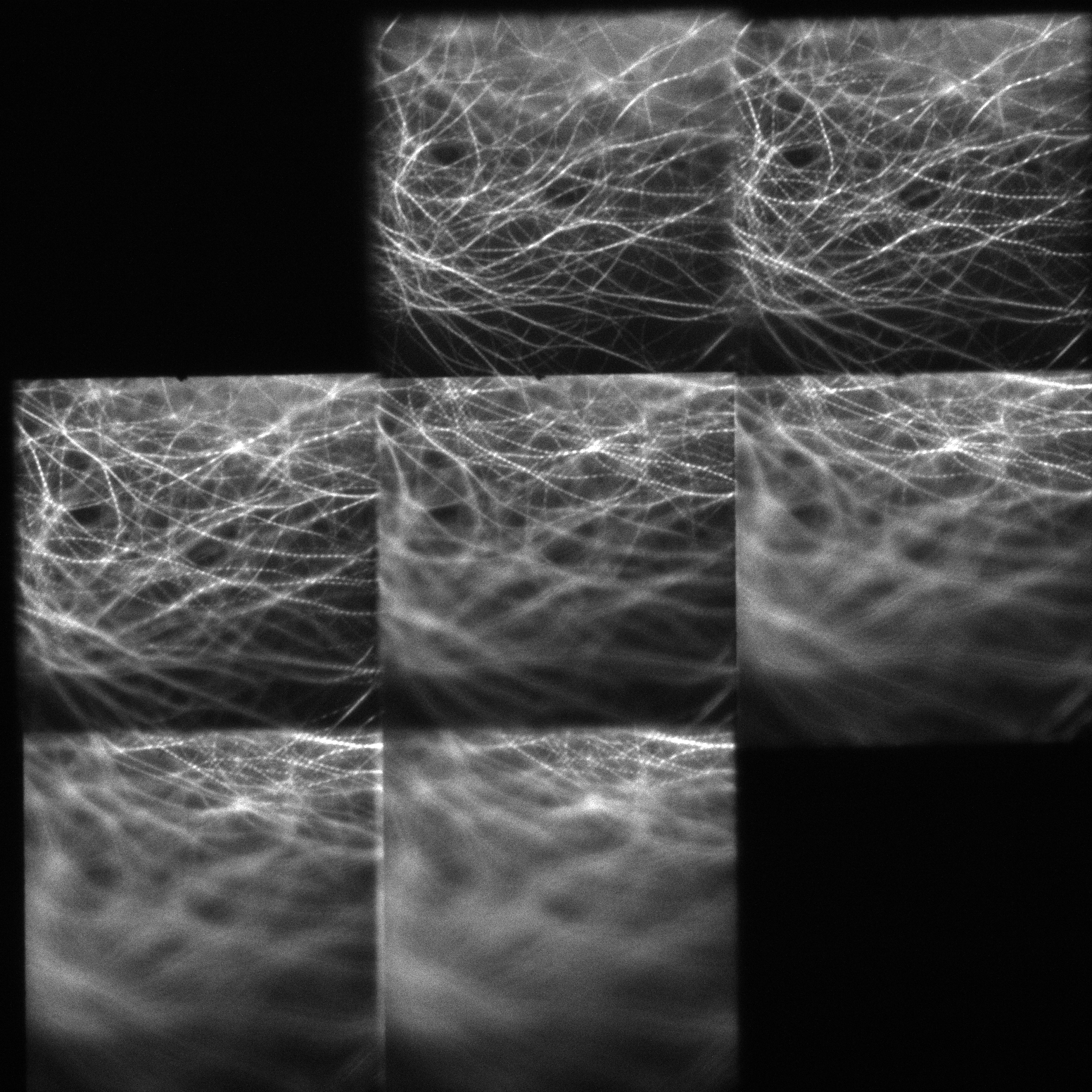

Supplement: Supplementary file 2 [file boe-15-4-2281-d001.zip › fig1/Tubulin_image/raw_data/img_channel000_position000_time000000000_z000.tif]

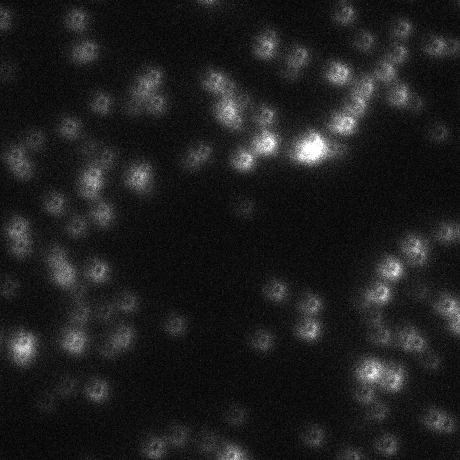

Supplement: Supplementary file 2 [file boe-15-4-2281-d001.zip › fig1/bead/mf_sim/raw_mfsim_pixelwise_reg.tif]

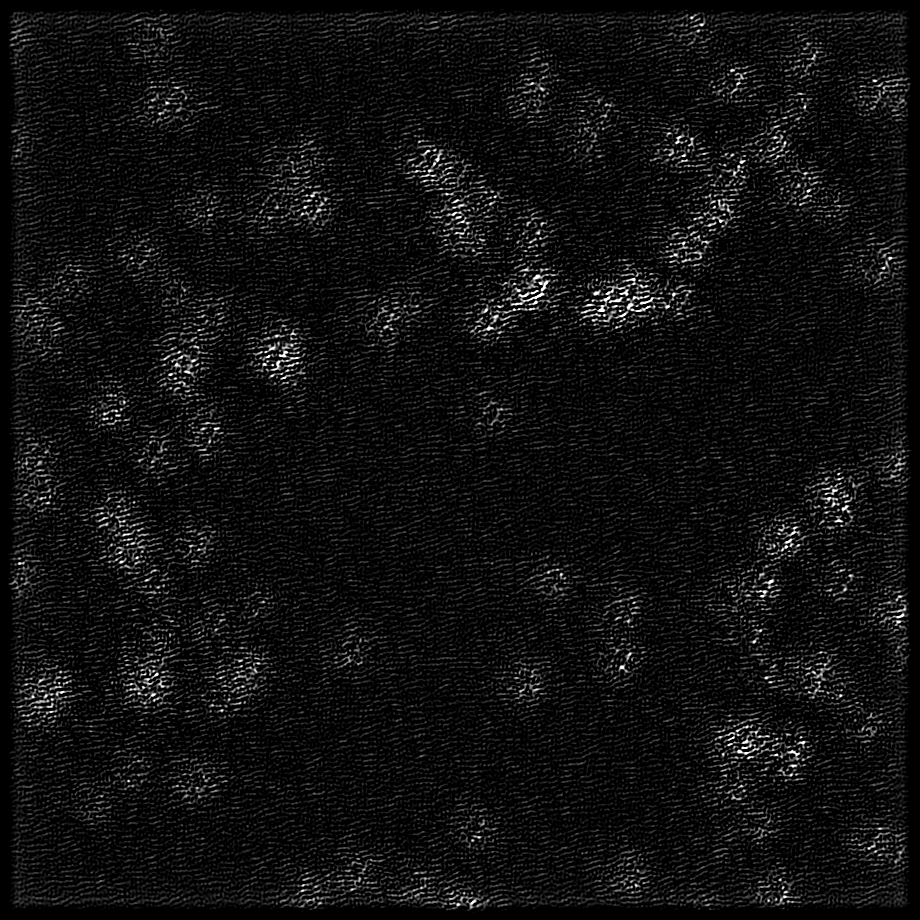

Supplement: Supplementary file 2 [file boe-15-4-2281-d001.zip › fig1/bead/mf_sim/recon_subpixelwise_reg.tif]

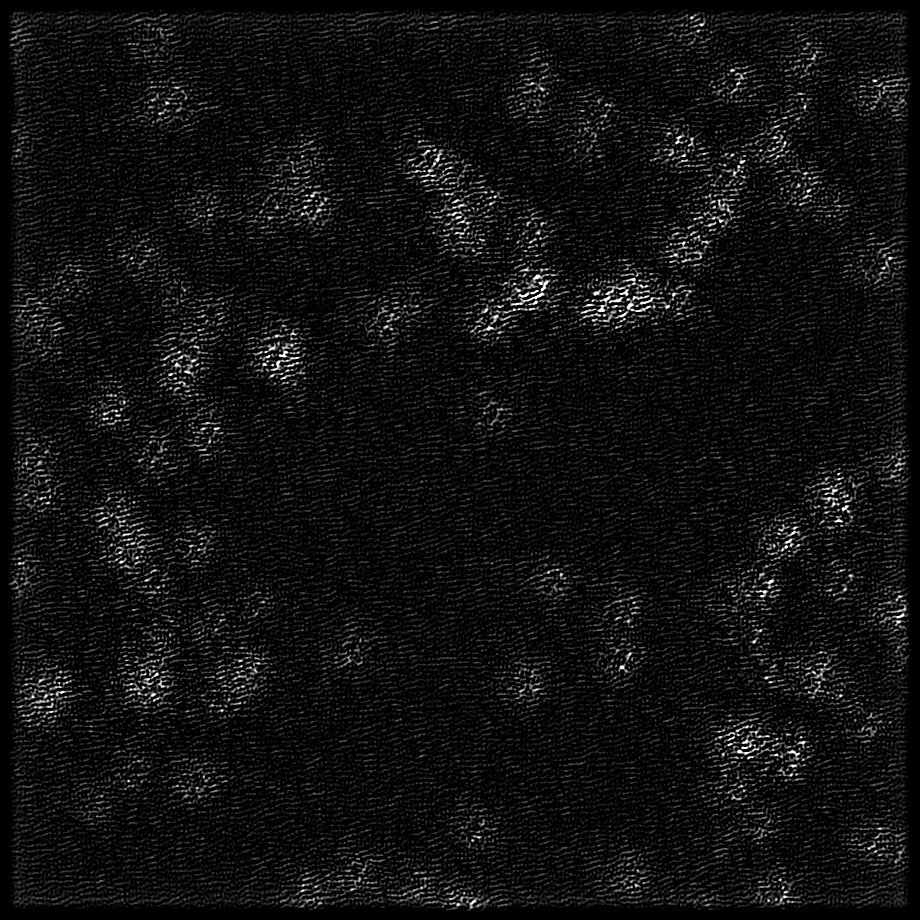

Supplement: Supplementary file 2 [file boe-15-4-2281-d001.zip › fig1/bead/mf_sim/recon_att10_Apo5_bet10_w16.tif]

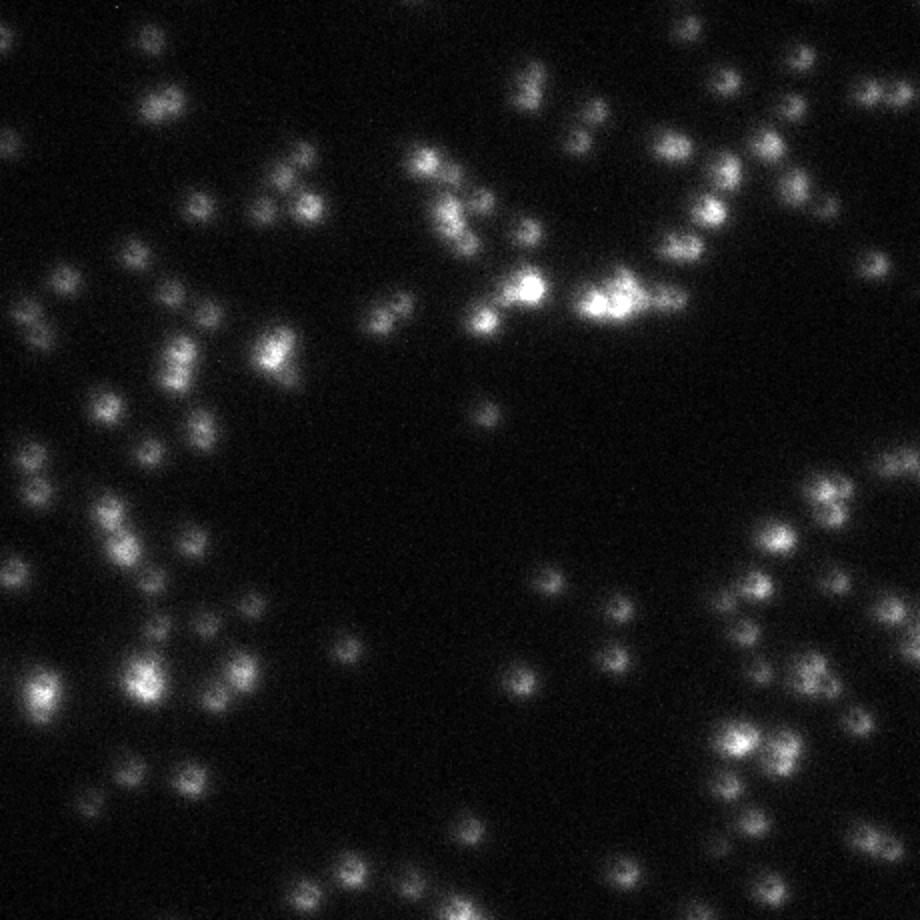

Supplement: Supplementary file 2 [file boe-15-4-2281-d001.zip › fig1/bead/wf/raw_wf_pixelwise_reg.tif]

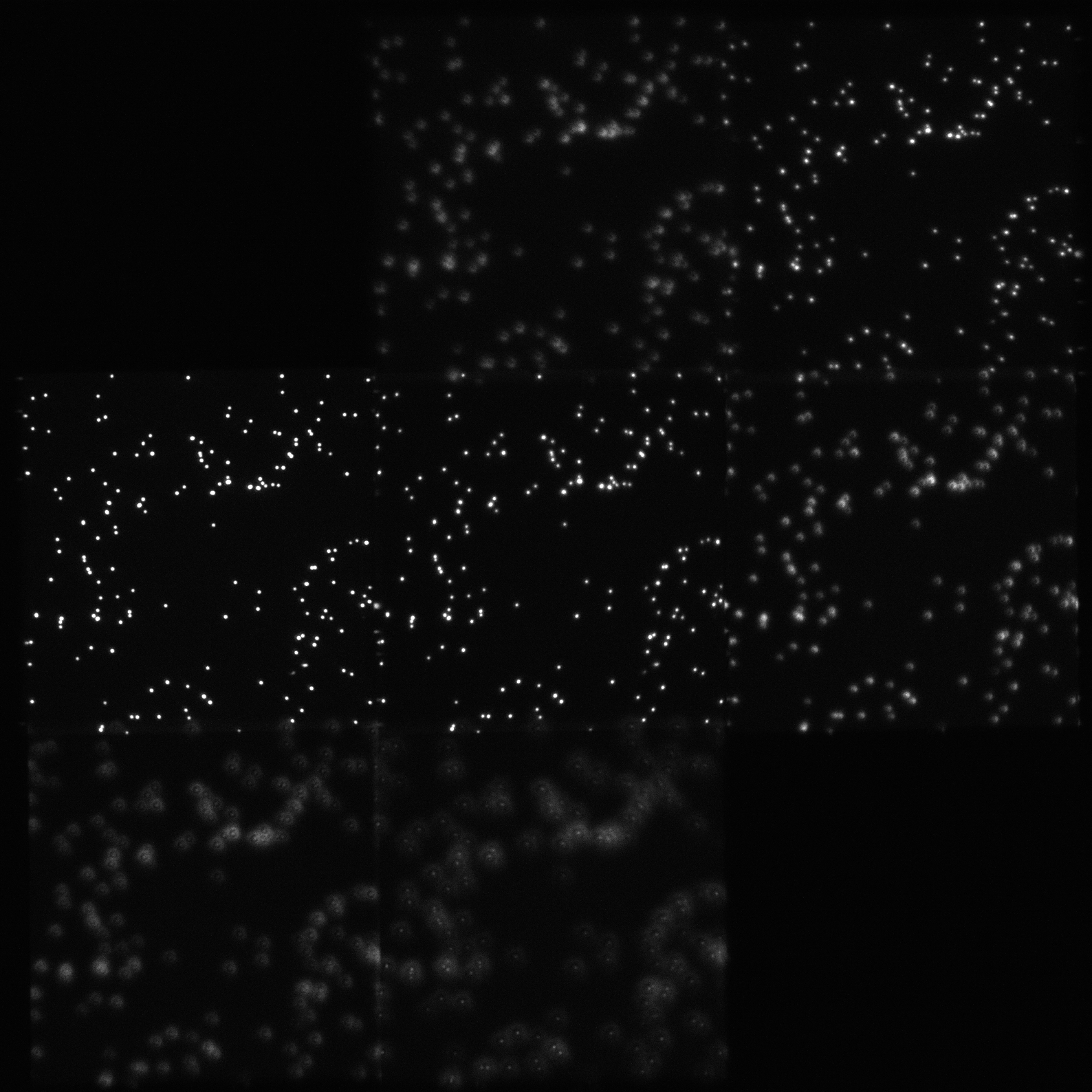

Supplement: Supplementary file 2 [file boe-15-4-2281-d001.zip › fig1/bead/wf/raw_wf.tif]

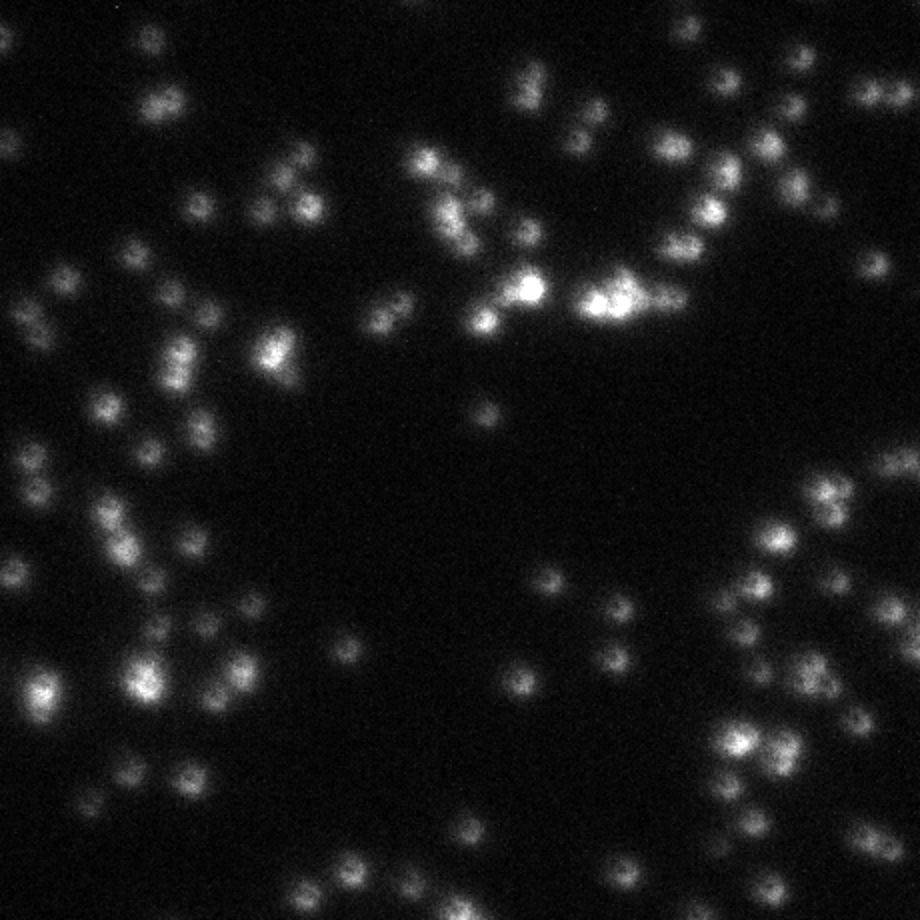

Supplement: Supplementary file 2 [file boe-15-4-2281-d001.zip › fig1/bead/wf/wf_subpixelwise_reg.tif]

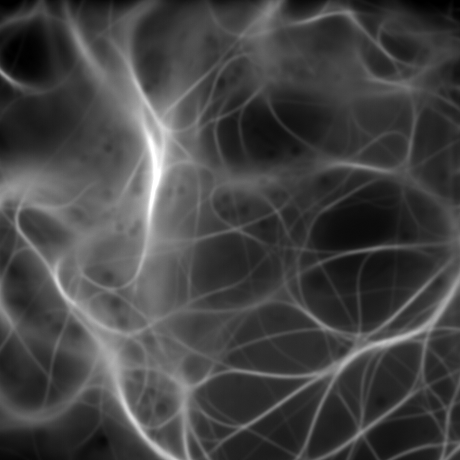

Supplement: Supplementary file 3 [file boe-15-4-2281-d002.zip › fig2/tubulin/wf_pixelwise_reg.tif]

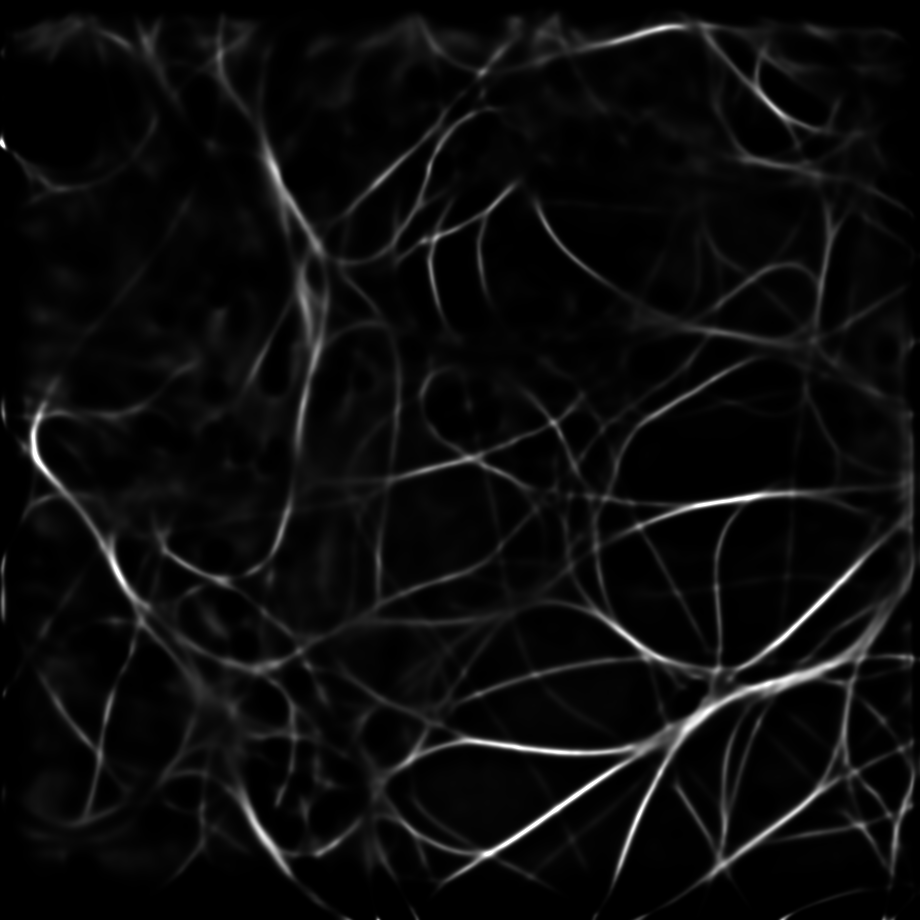

Supplement: Supplementary file 3 [file boe-15-4-2281-d002.zip › fig2/tubulin/wf_decon.tif]

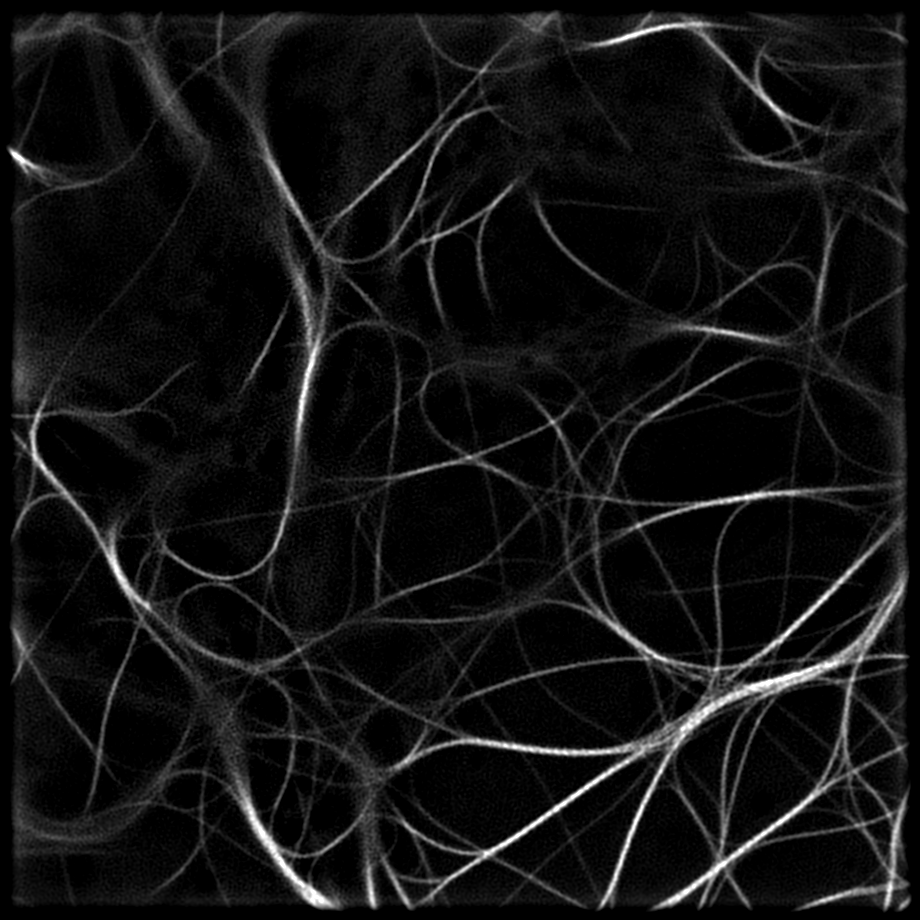

Supplement: Supplementary file 3 [file boe-15-4-2281-d002.zip › fig2/tubulin/mf_sim_recon_att10_Apo5_bet10_w120_subpixel_reg.tif]

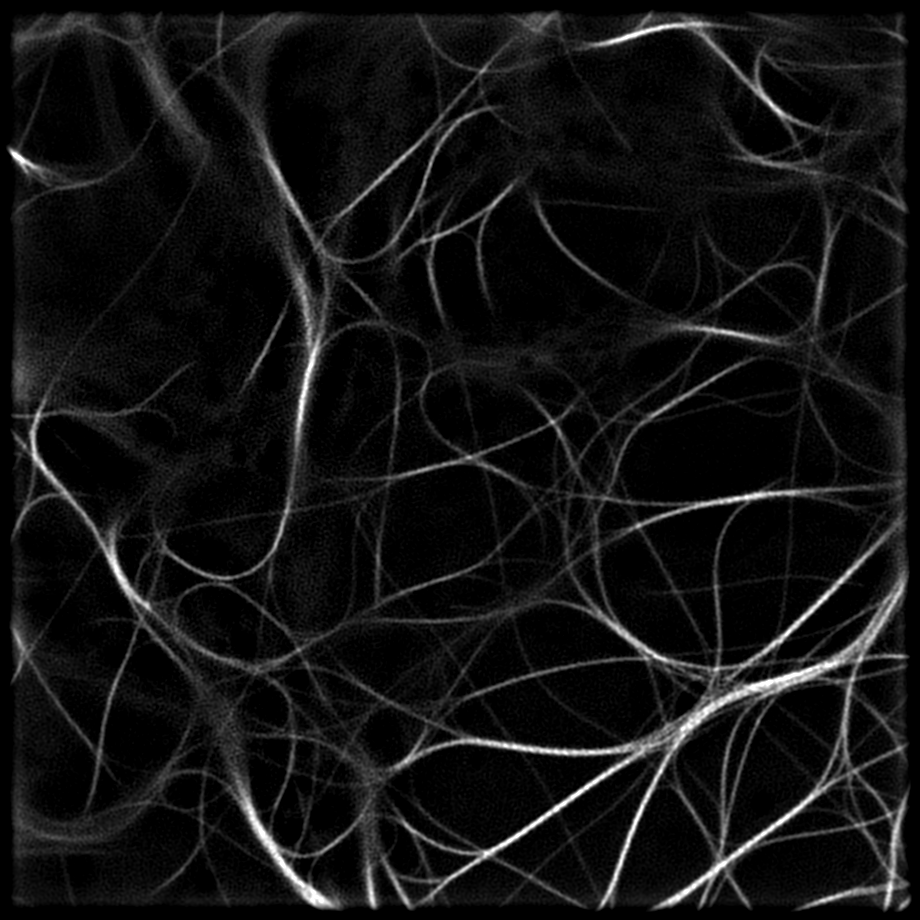

Supplement: Supplementary file 3 [file boe-15-4-2281-d002.zip › fig2/tubulin/mf_sim_recon_att10_Apo5_bet10_w120.tif]

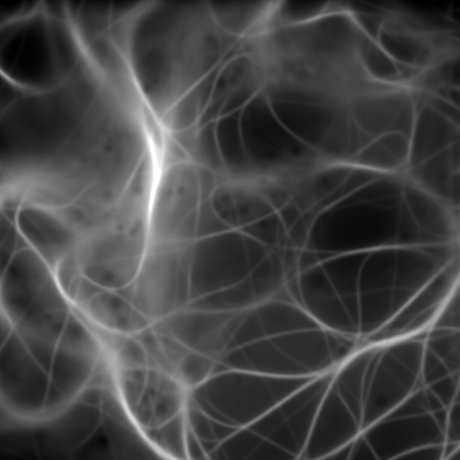

Supplement: Supplementary file 3 [file boe-15-4-2281-d002.zip › fig2/tubulin/wf_subpixelwise_reg.tif]

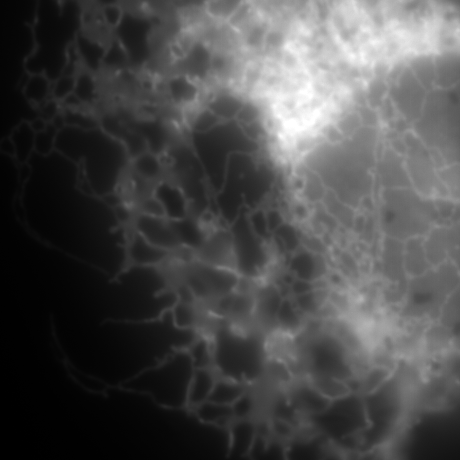

Supplement: Supplementary file 3 [file boe-15-4-2281-d002.zip › fig2/ER/wf_pixelwise_reg.tif]

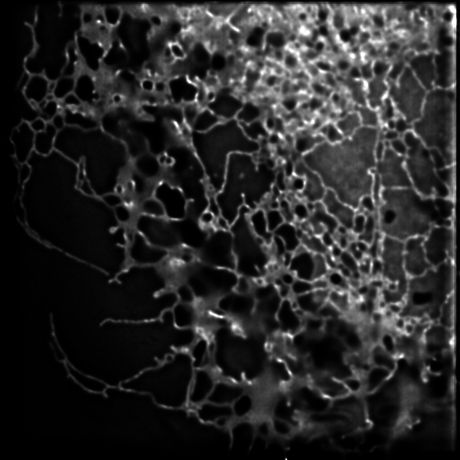

Supplement: Supplementary file 3 [file boe-15-4-2281-d002.zip › fig2/ER/wf_decon.tif]

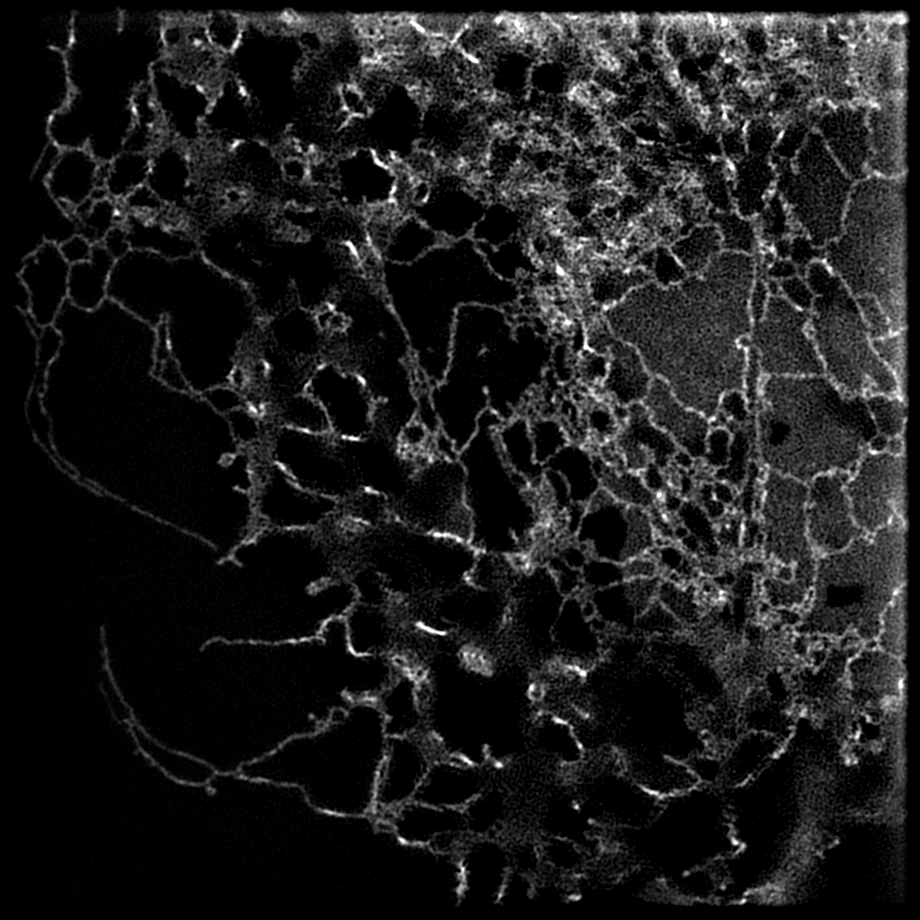

Supplement: Supplementary file 3 [file boe-15-4-2281-d002.zip › fig2/ER/mf_sim_recon_att10_Apo5_bet10_w120_subpixel_reg.tif]

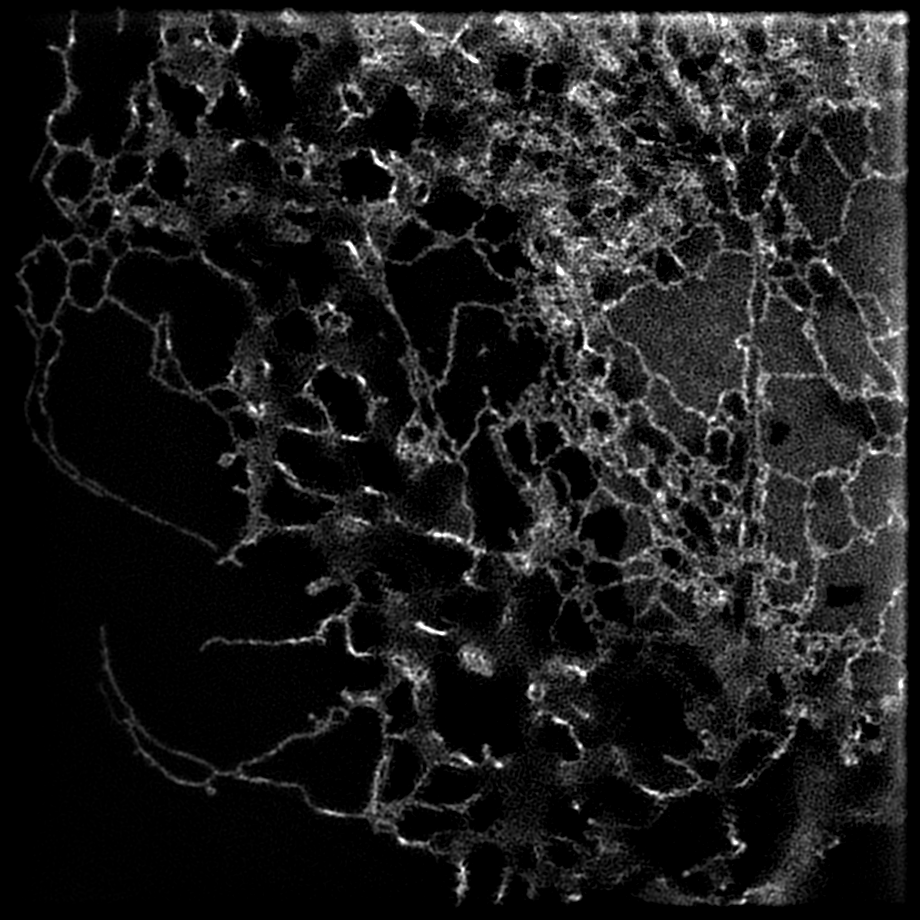

Supplement: Supplementary file 3 [file boe-15-4-2281-d002.zip › fig2/ER/mf_sim_recon_att10_Apo5_bet10_w120.tif]

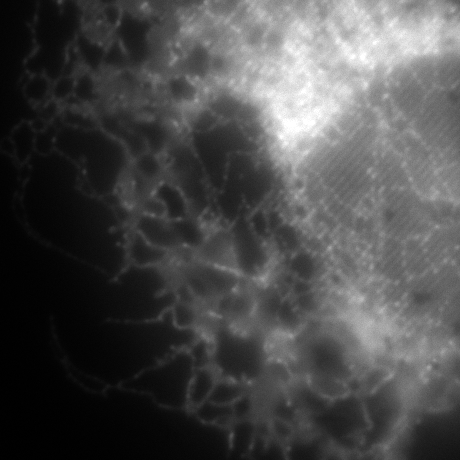

Supplement: Supplementary file 3 [file boe-15-4-2281-d002.zip › fig2/ER/mf_sim_pixelwise_reg.tif]

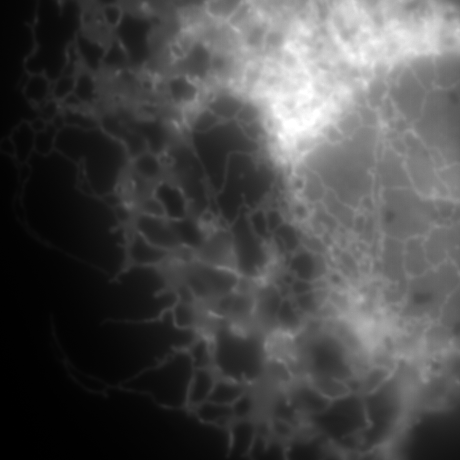

Supplement: Supplementary file 3 [file boe-15-4-2281-d002.zip › fig2/ER/wf_subpixelwise_reg.tif]

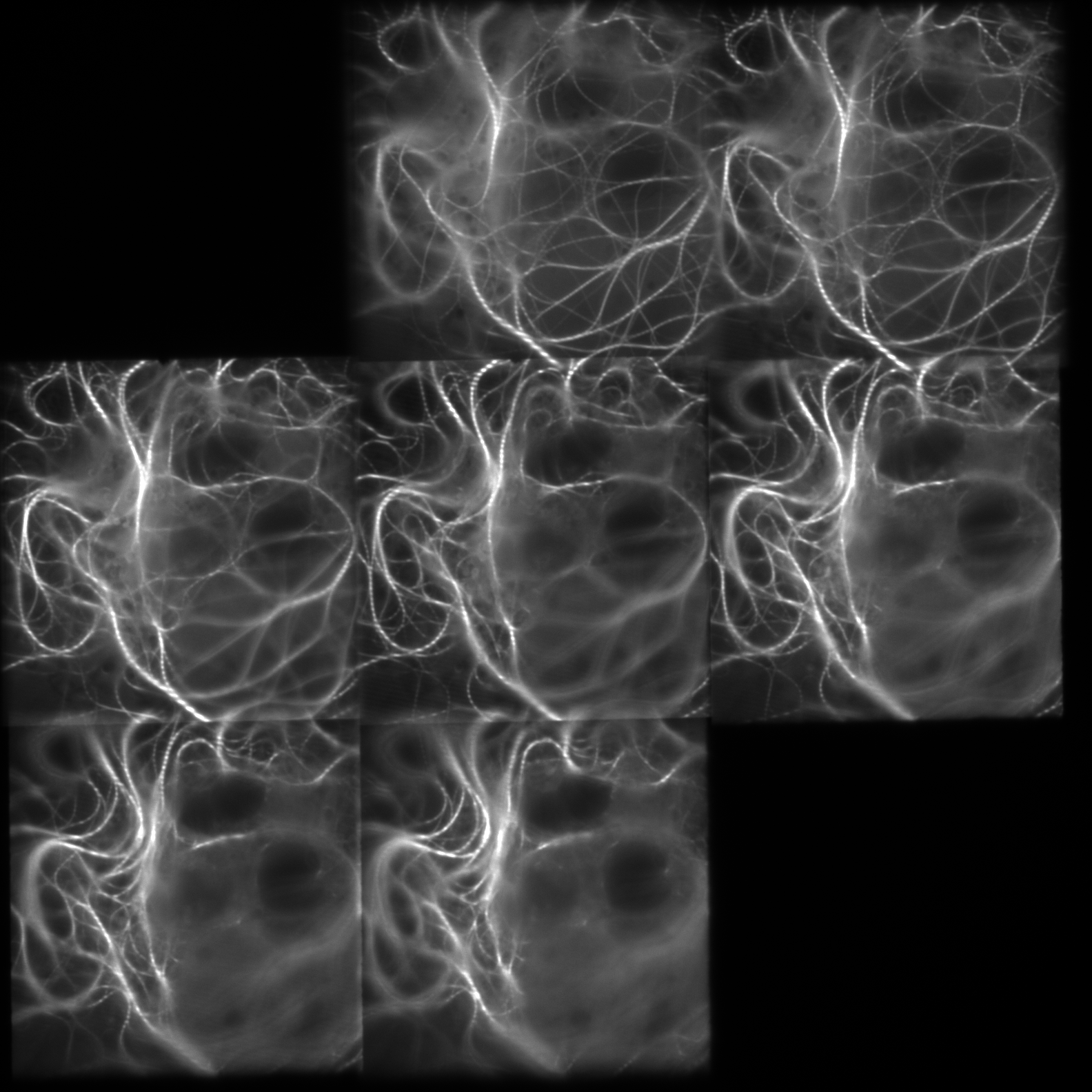

Supplement: Supplementary file 3 [file boe-15-4-2281-d002.zip › fig2/tubulin/raw/img_channel000_position000_time000000005_z000.tif]

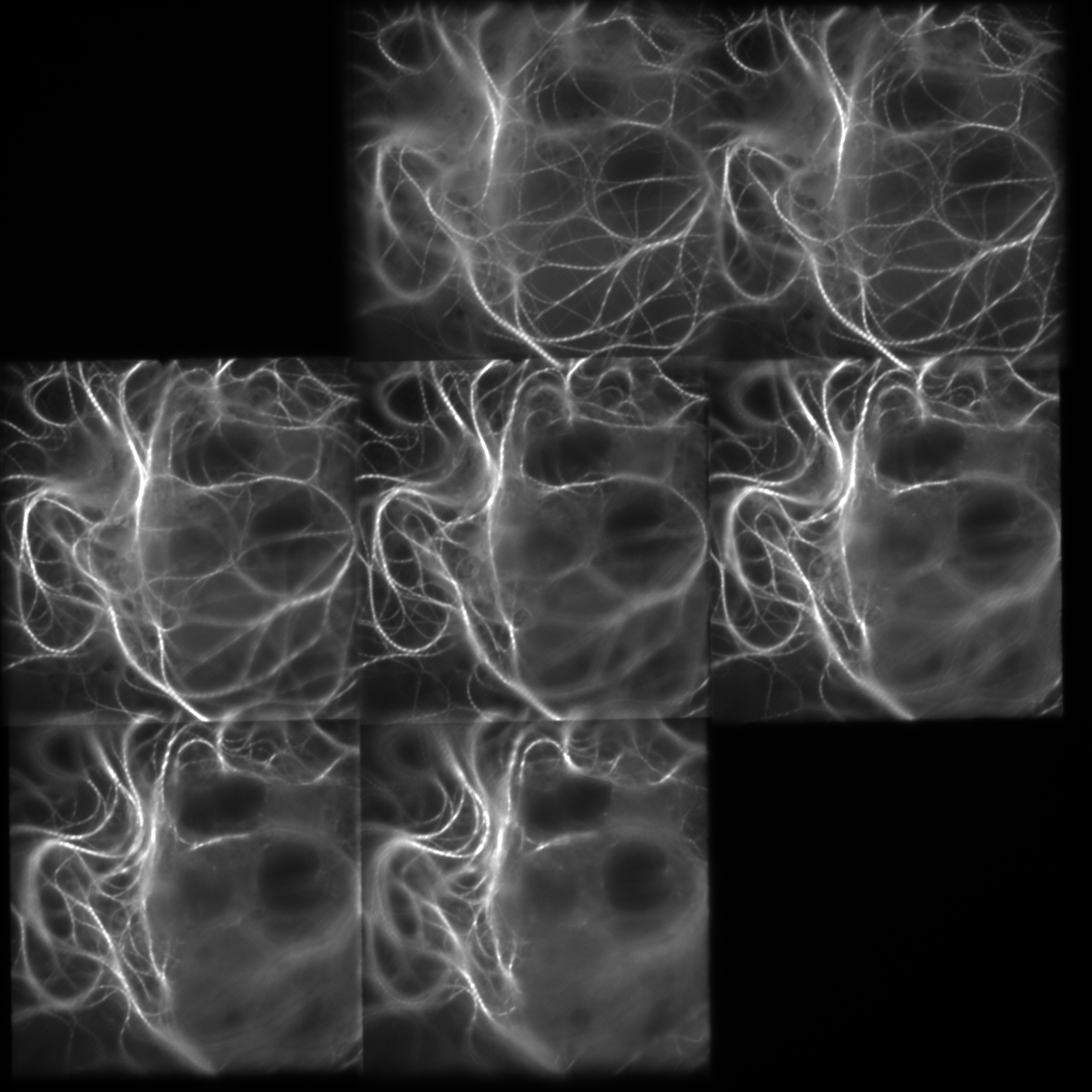

Supplement: Supplementary file 3 [file boe-15-4-2281-d002.zip › fig2/tubulin/raw/img_channel000_position000_time000000004_z000.tif]

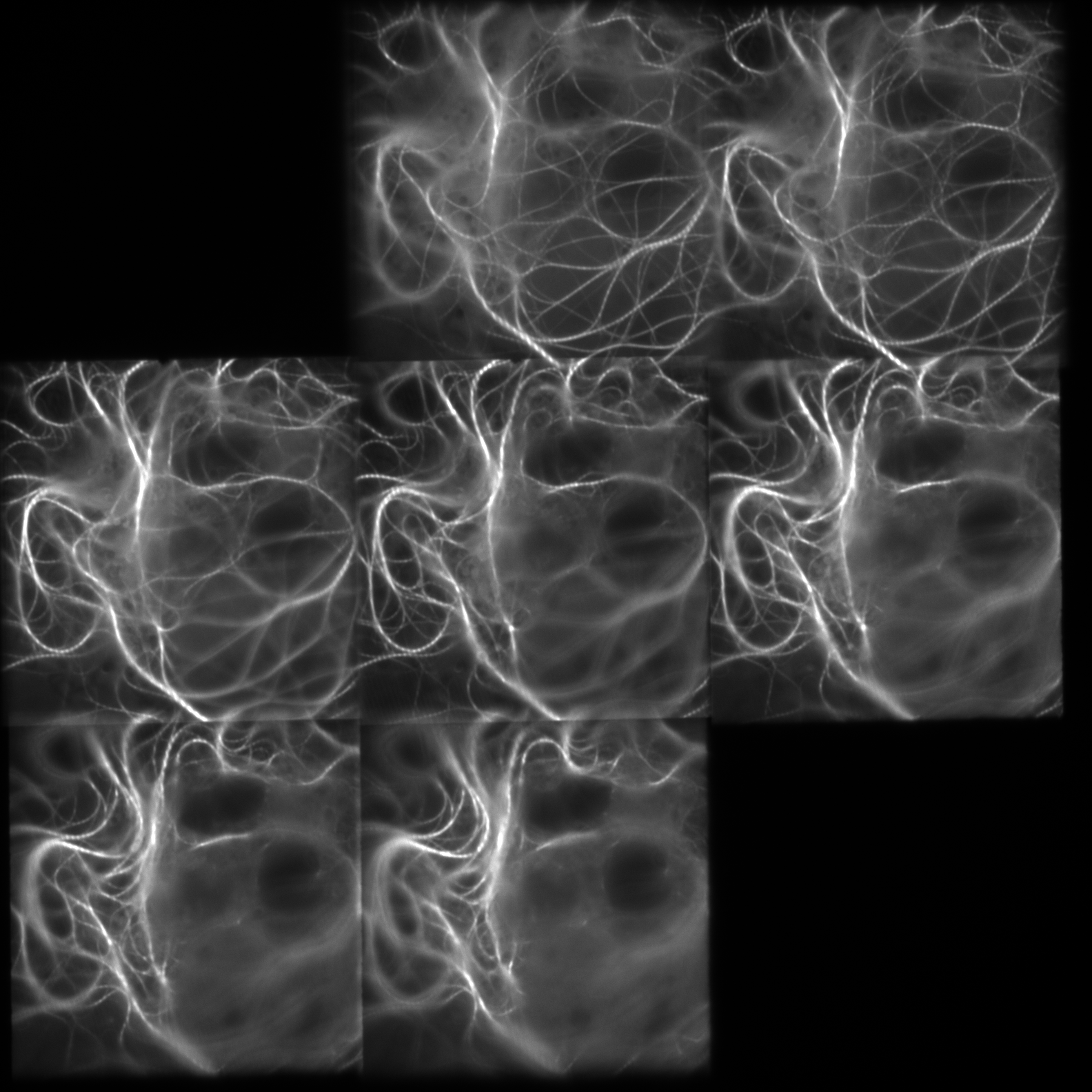

Supplement: Supplementary file 3 [file boe-15-4-2281-d002.zip › fig2/tubulin/raw/img_channel000_position000_time000000012_z000.tif]

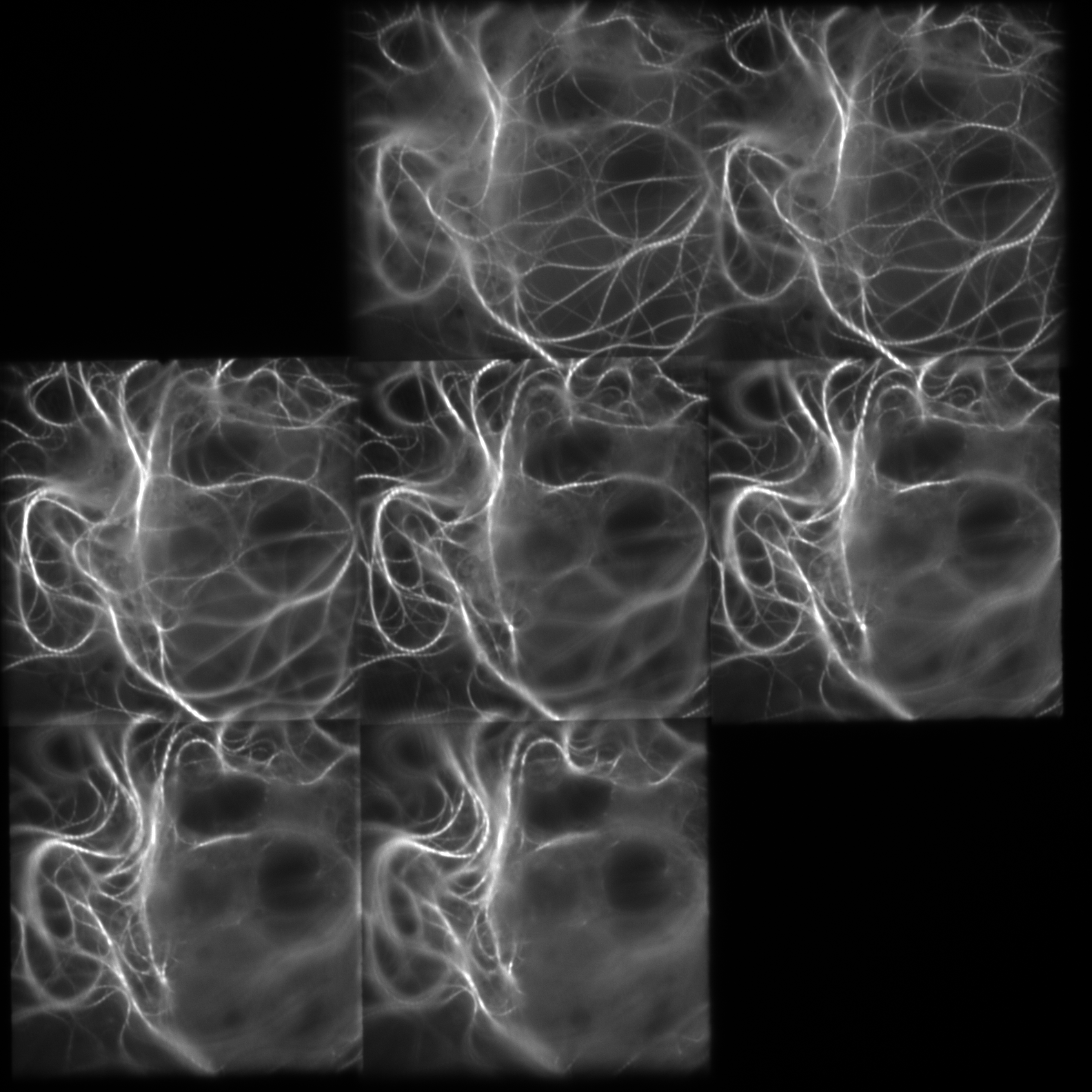

Supplement: Supplementary file 3 [file boe-15-4-2281-d002.zip › fig2/tubulin/raw/img_channel000_position000_time000000013_z000.tif]

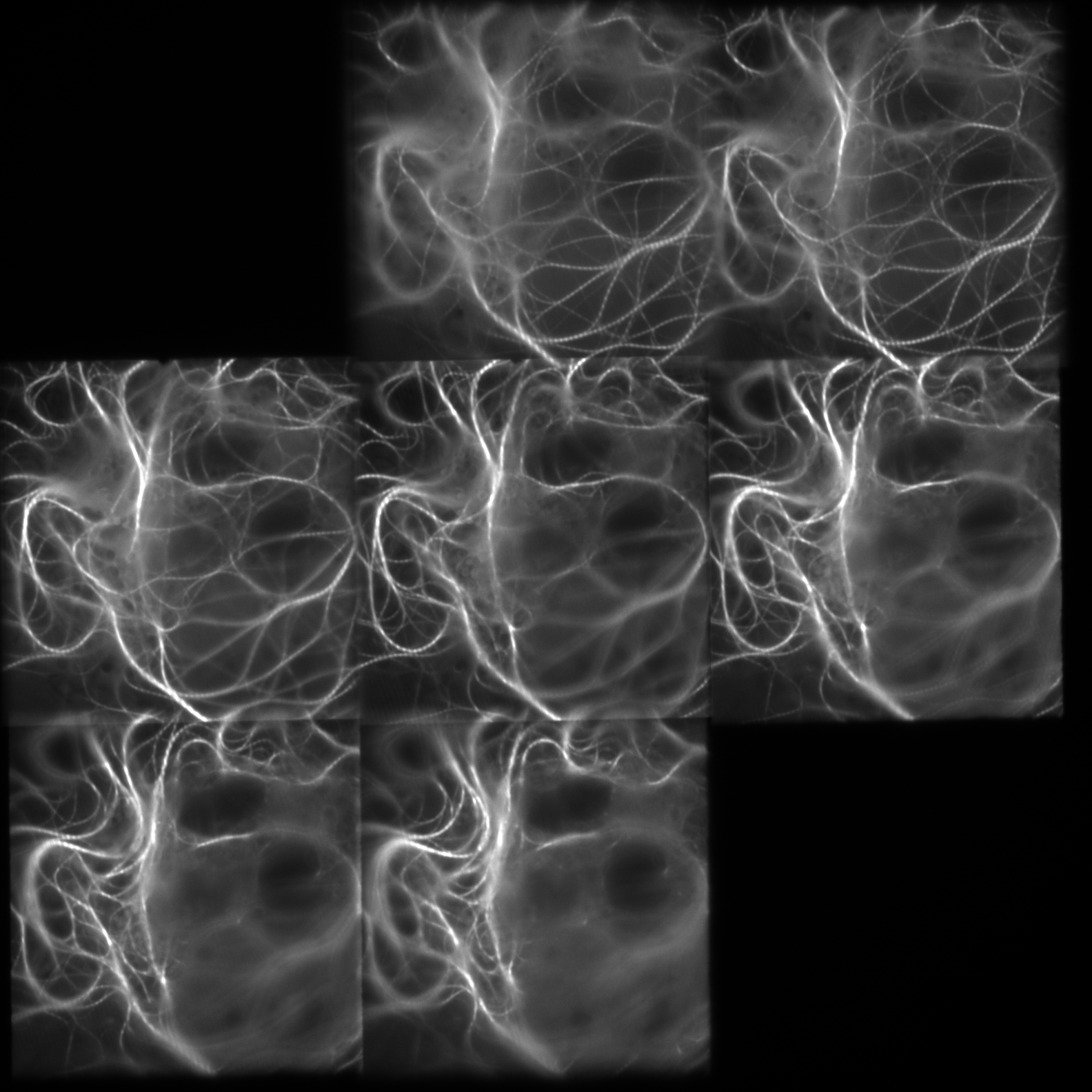

Supplement: Supplementary file 3 [file boe-15-4-2281-d002.zip › fig2/tubulin/raw/img_channel000_position000_time000000026_z000.tif]

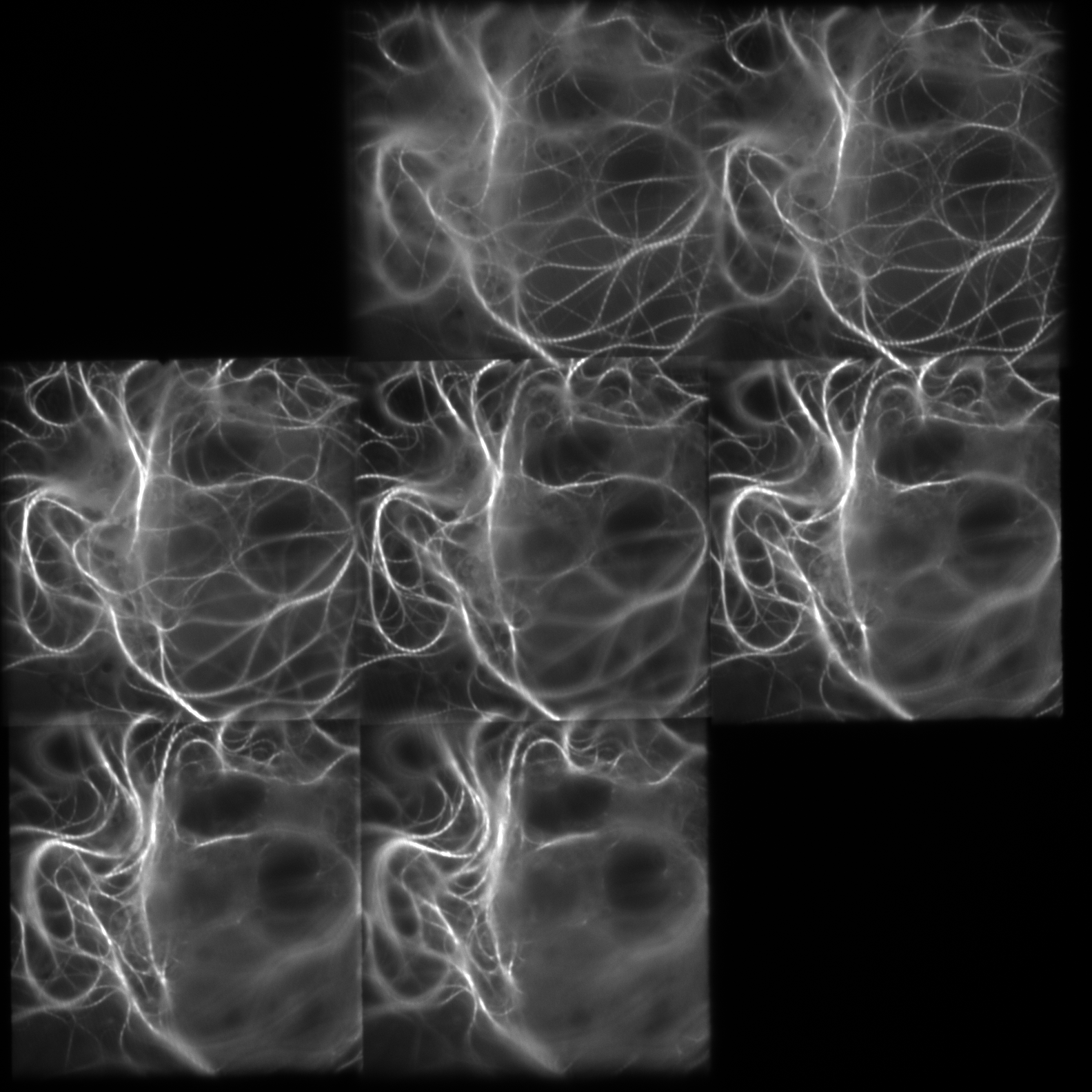

Supplement: Supplementary file 3 [file boe-15-4-2281-d002.zip › fig2/tubulin/raw/img_channel000_position000_time000000027_z000.tif]

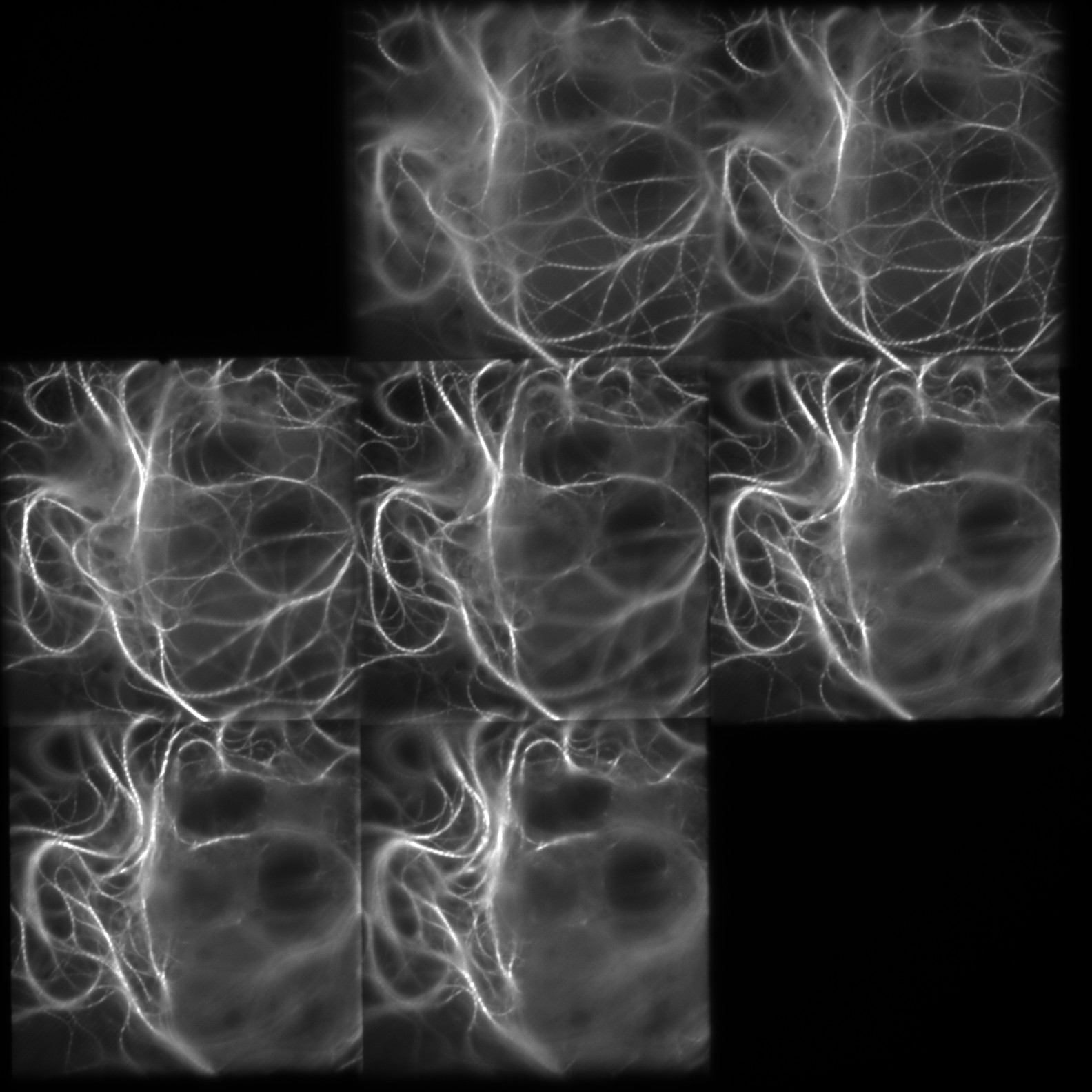

Supplement: Supplementary file 3 [file boe-15-4-2281-d002.zip › fig2/tubulin/raw/img_channel000_position000_time000000018_z000.tif]

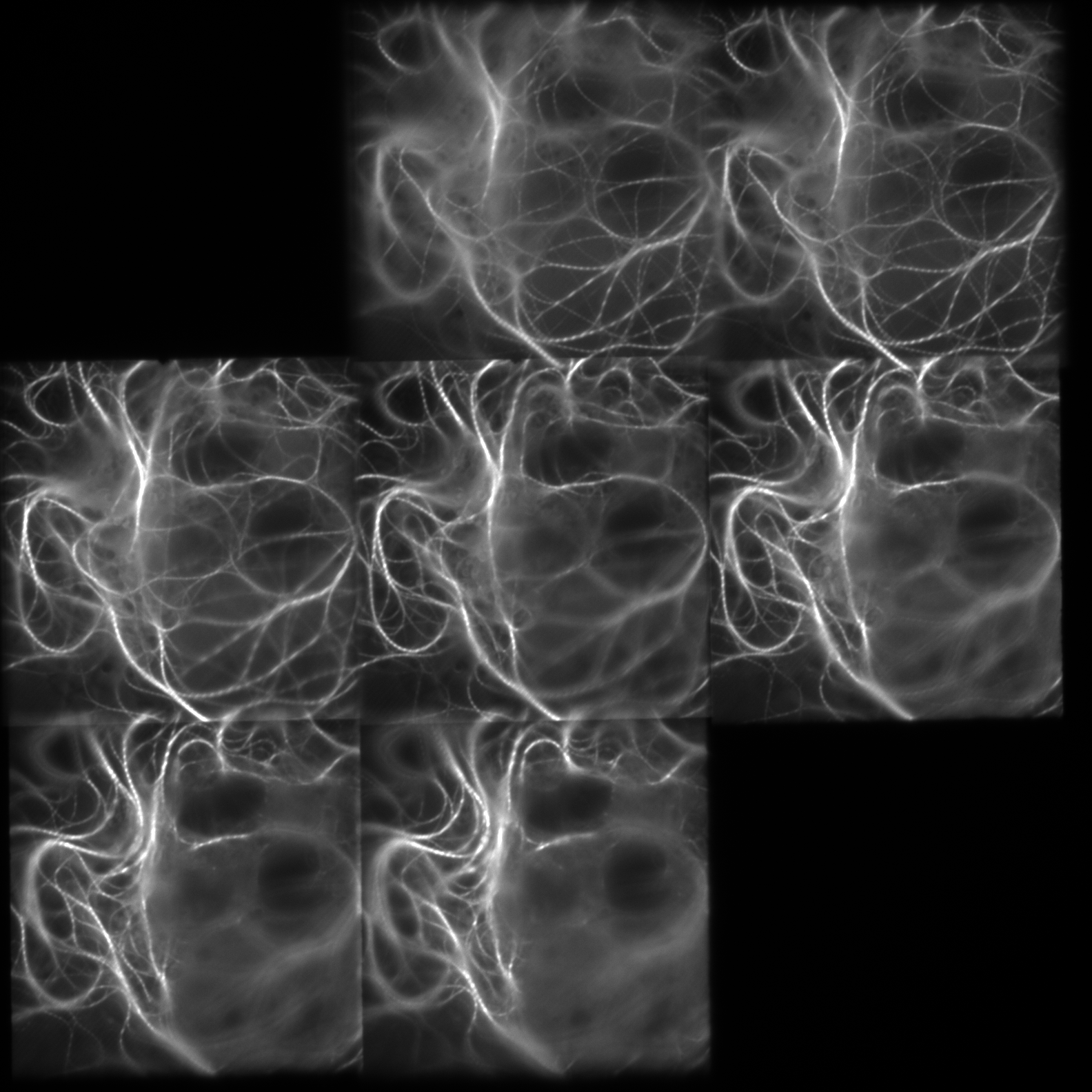

Supplement: Supplementary file 3 [file boe-15-4-2281-d002.zip › fig2/tubulin/raw/img_channel000_position000_time000000019_z000.tif]

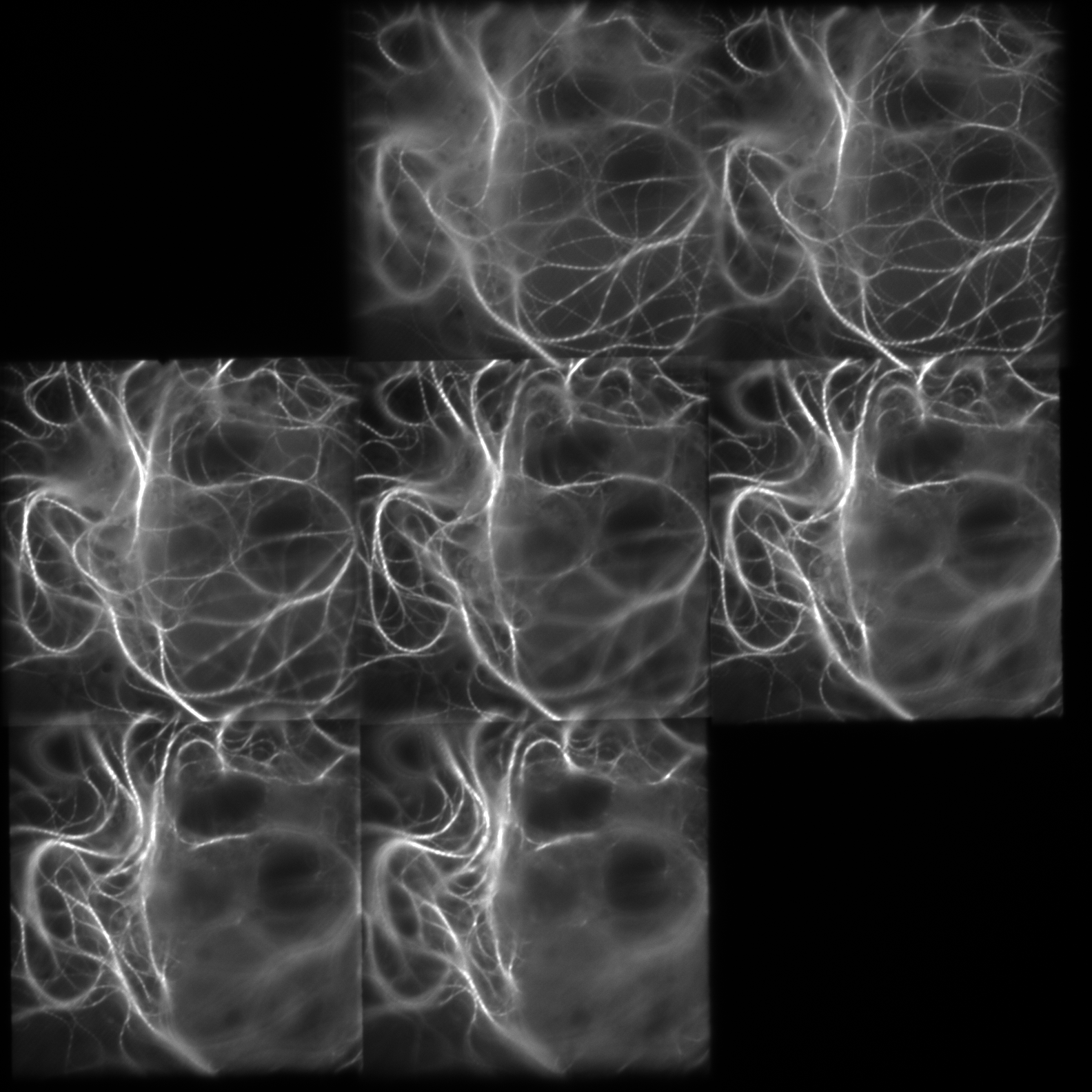

Supplement: Supplementary file 3 [file boe-15-4-2281-d002.zip › fig2/tubulin/raw/img_channel000_position000_time000000015_z000.tif]

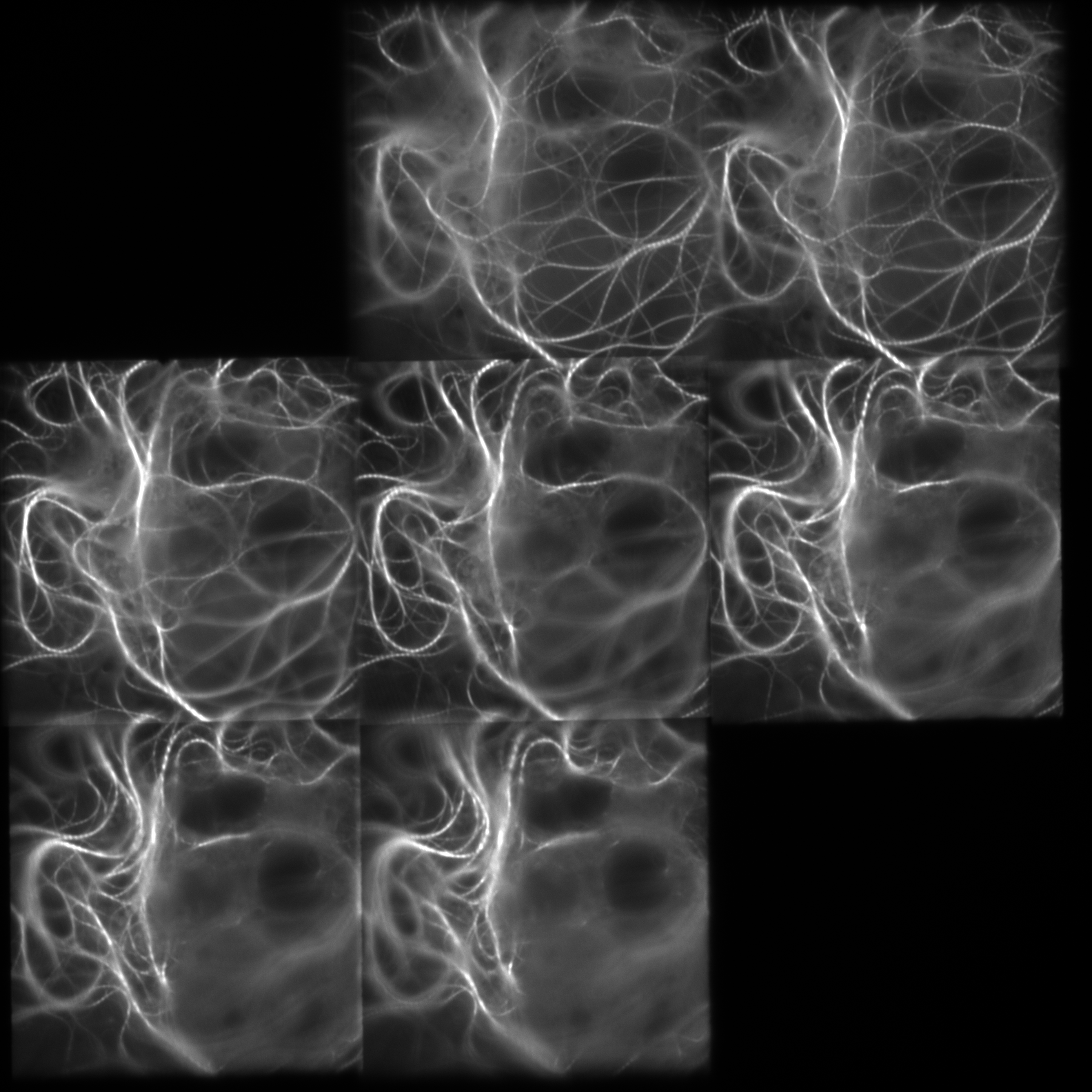

Supplement: Supplementary file 3 [file boe-15-4-2281-d002.zip › fig2/tubulin/raw/img_channel000_position000_time000000014_z000.tif]

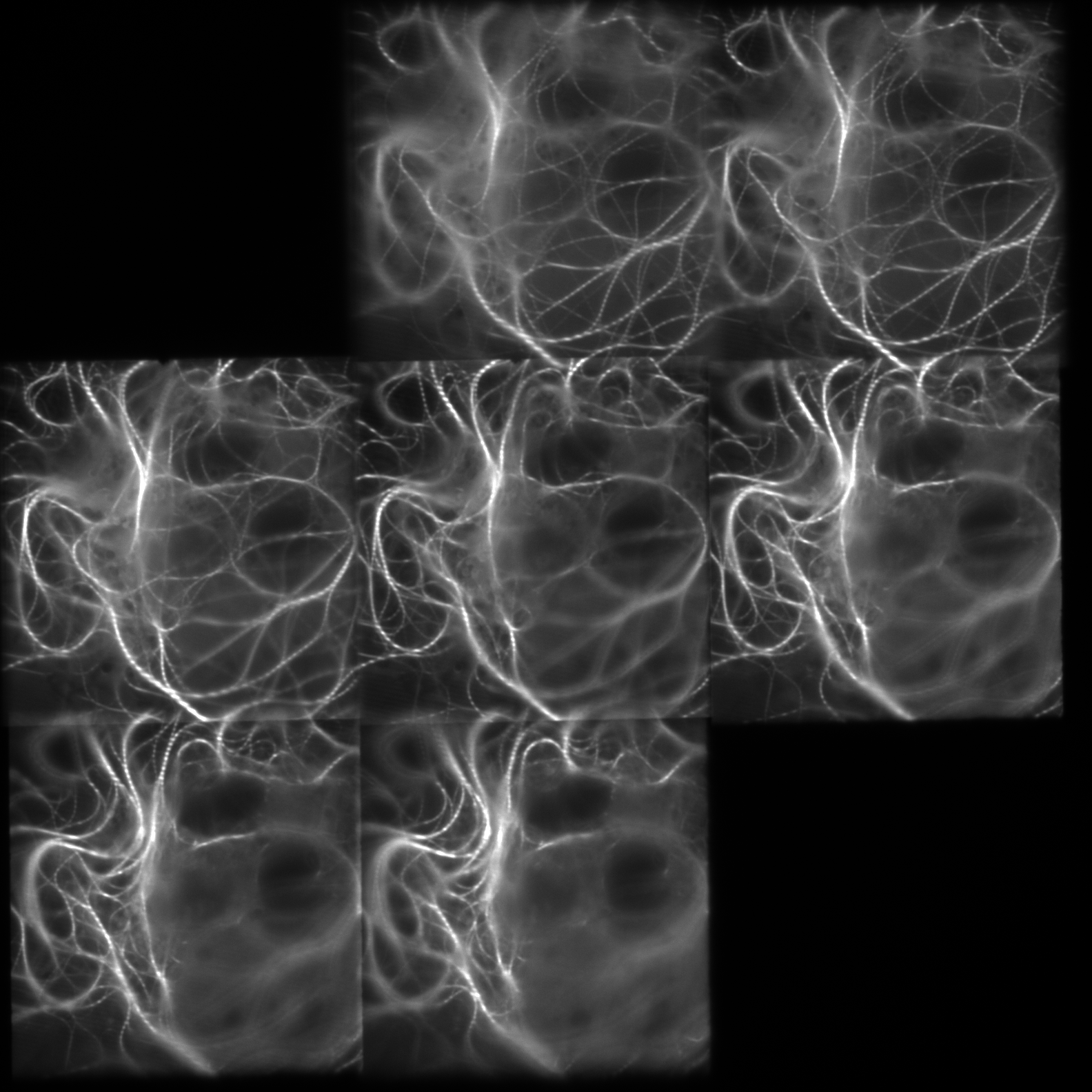

Supplement: Supplementary file 3 [file boe-15-4-2281-d002.zip › fig2/tubulin/raw/img_channel000_position000_time000000021_z000.tif]

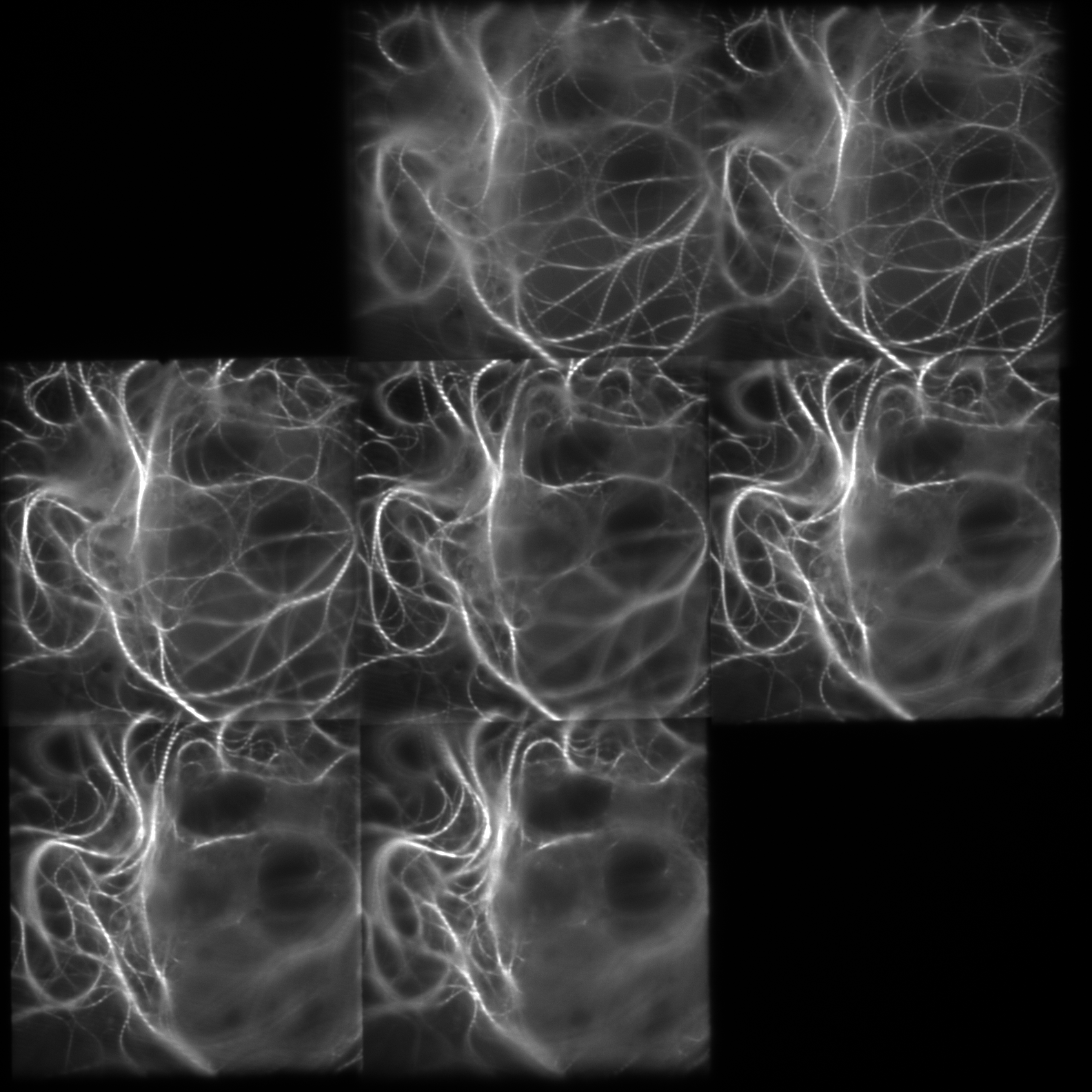

Supplement: Supplementary file 3 [file boe-15-4-2281-d002.zip › fig2/tubulin/raw/img_channel000_position000_time000000020_z000.tif]

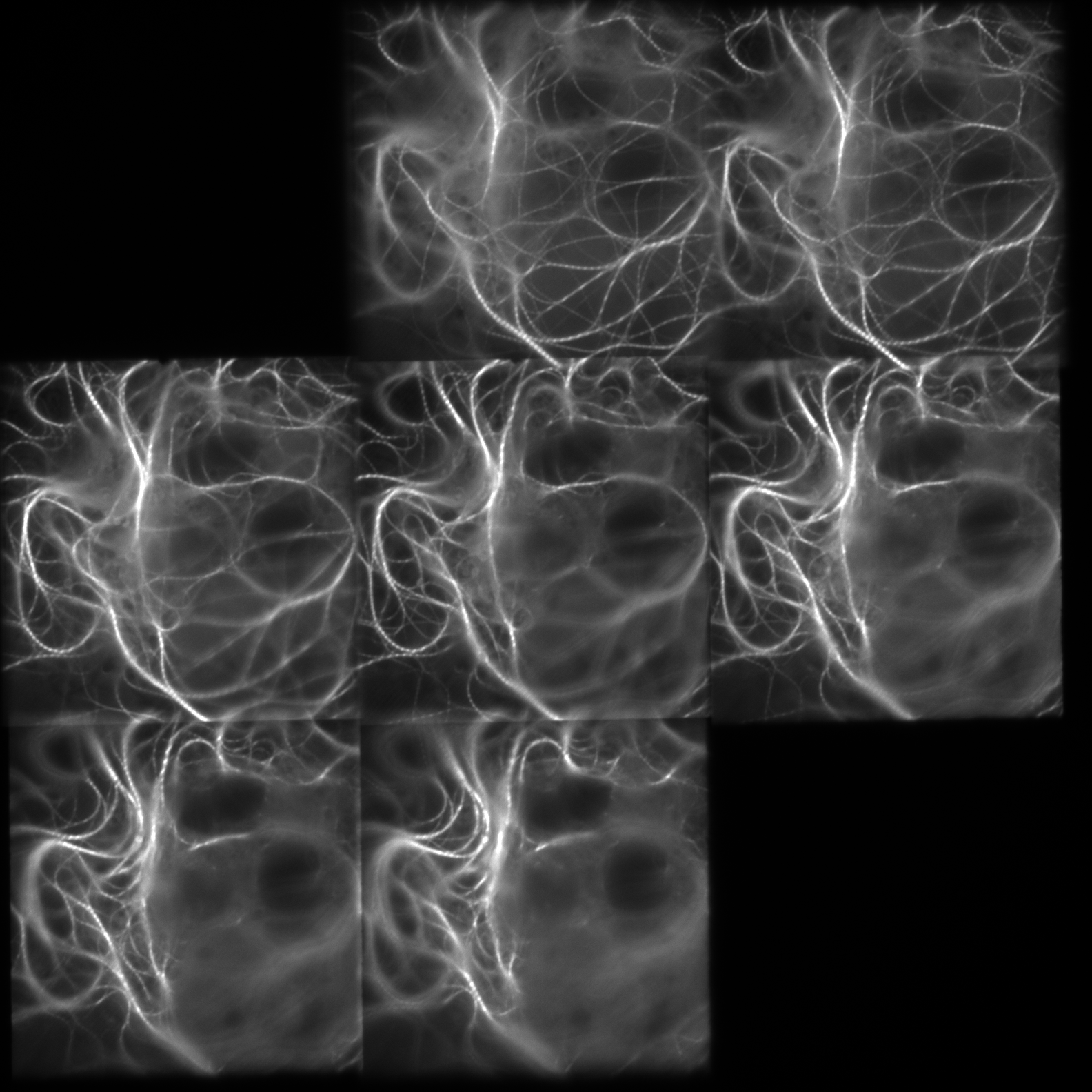

Supplement: Supplementary file 3 [file boe-15-4-2281-d002.zip › fig2/tubulin/raw/img_channel000_position000_time000000002_z000.tif]

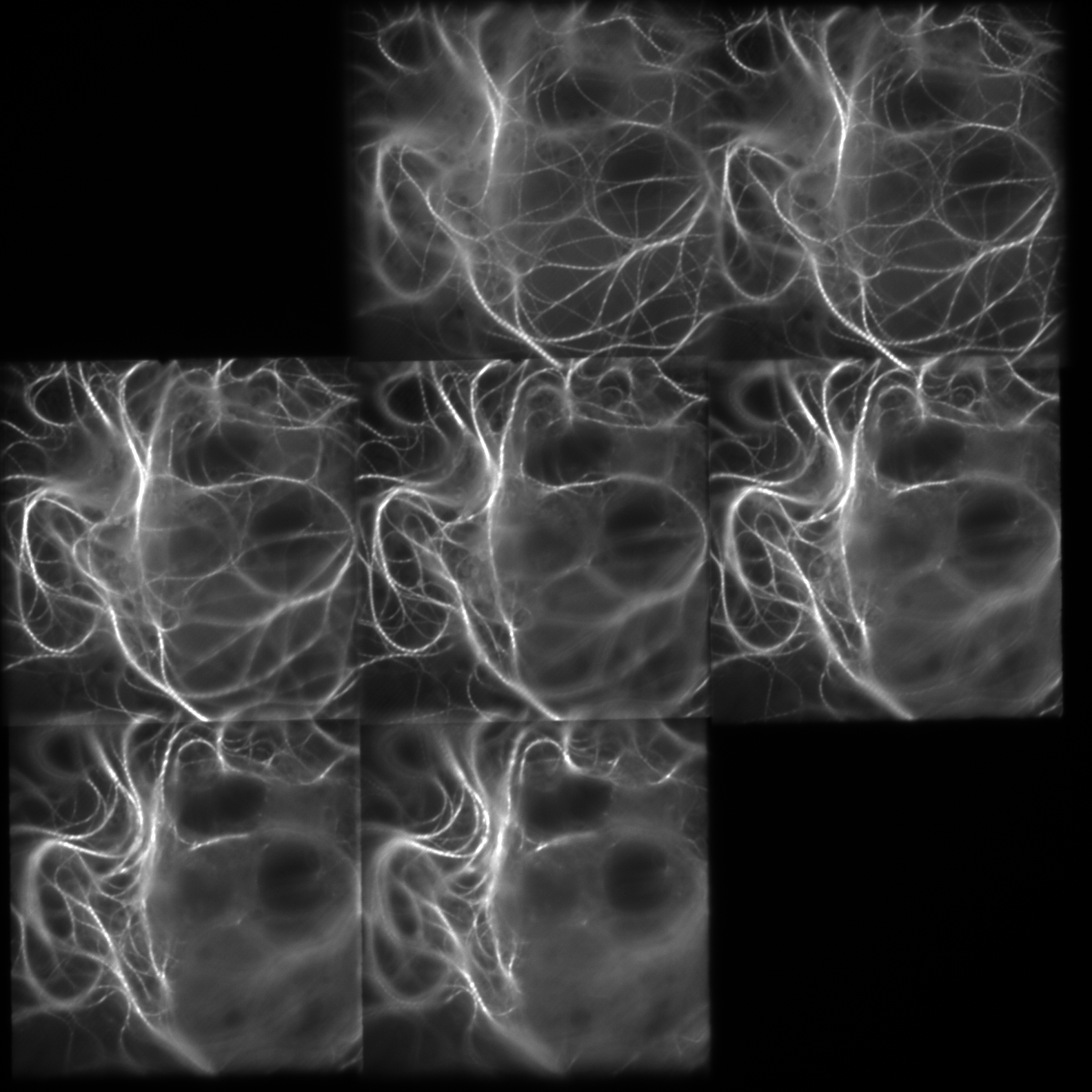

Supplement: Supplementary file 3 [file boe-15-4-2281-d002.zip › fig2/tubulin/raw/img_channel000_position000_time000000003_z000.tif]

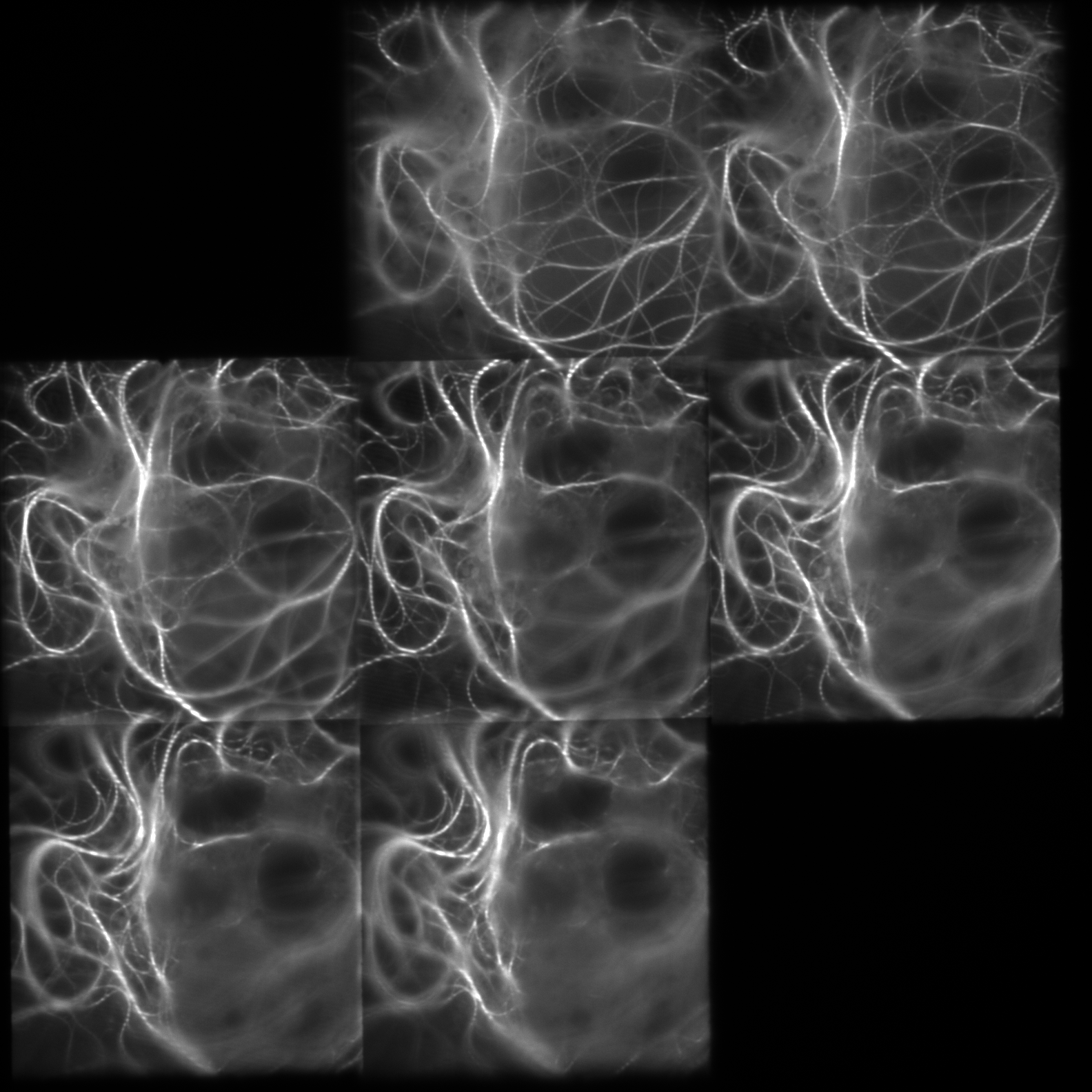

Supplement: Supplementary file 3 [file boe-15-4-2281-d002.zip › fig2/tubulin/raw/img_channel000_position000_time000000008_z000.tif]

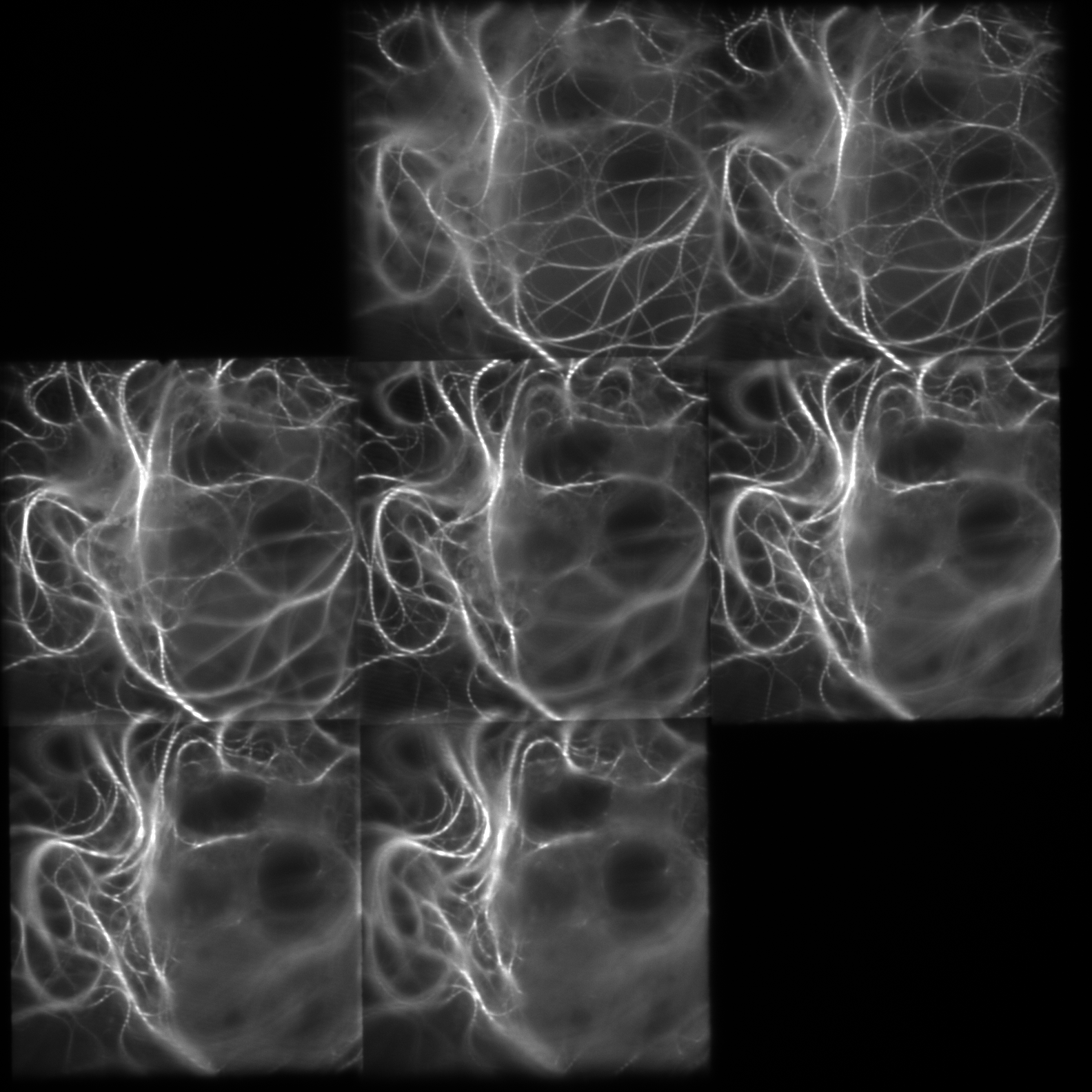

Supplement: Supplementary file 3 [file boe-15-4-2281-d002.zip › fig2/tubulin/raw/img_channel000_position000_time000000009_z000.tif]

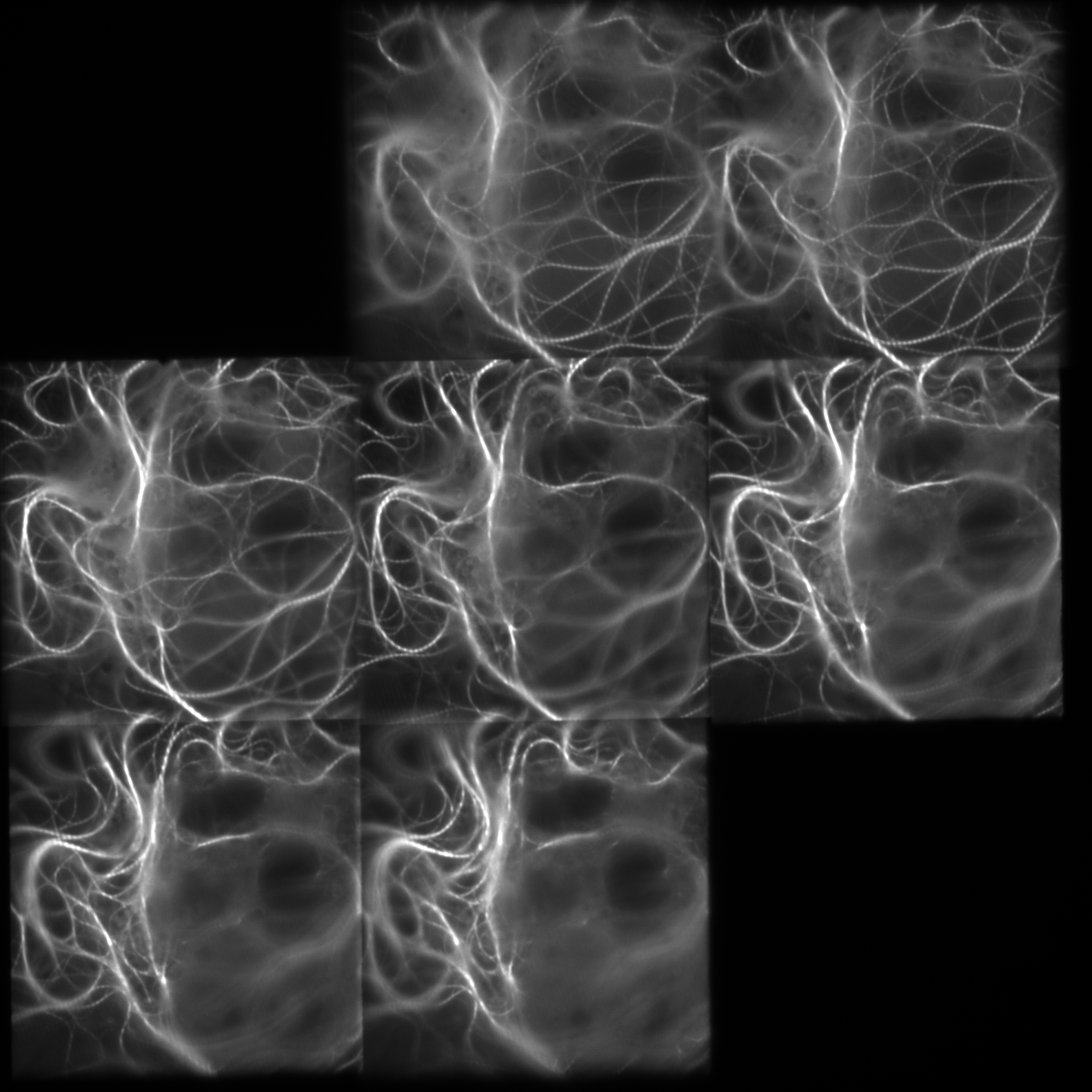

Supplement: Supplementary file 3 [file boe-15-4-2281-d002.zip › fig2/tubulin/raw/img_channel000_position000_time000000025_z000.tif]

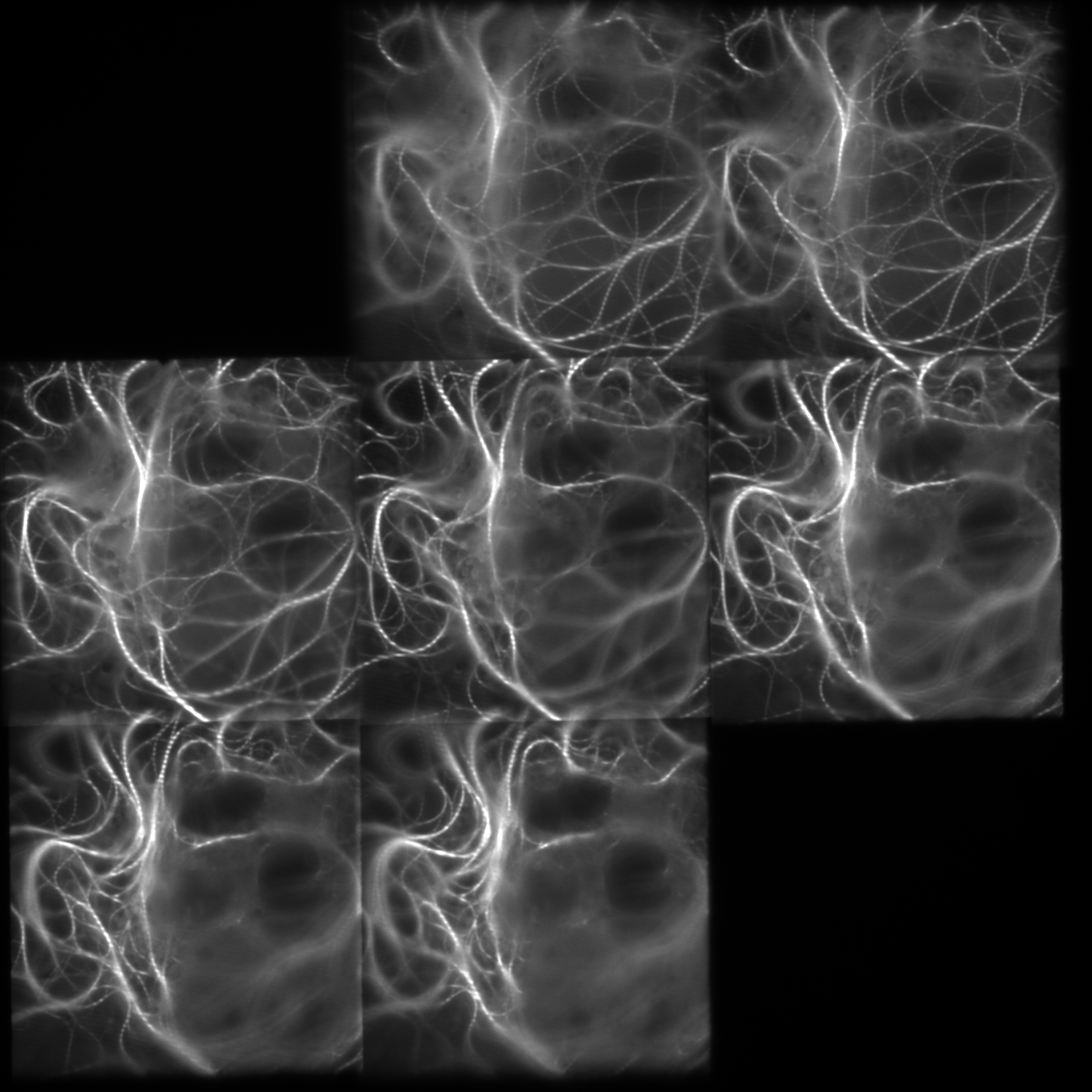

Supplement: Supplementary file 3 [file boe-15-4-2281-d002.zip › fig2/tubulin/raw/img_channel000_position000_time000000024_z000.tif]

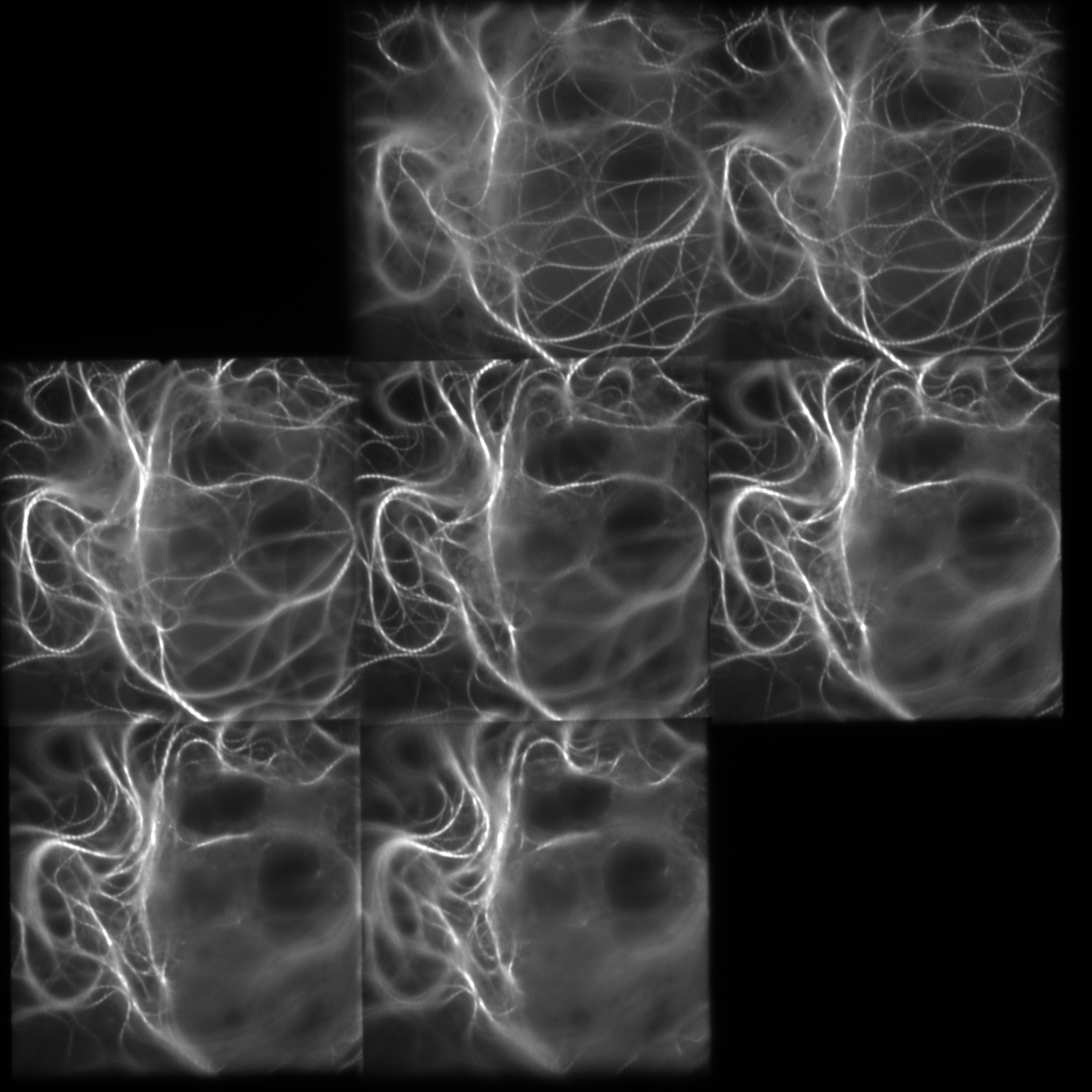

Supplement: Supplementary file 3 [file boe-15-4-2281-d002.zip › fig2/tubulin/raw/img_channel000_position000_time000000011_z000.tif]

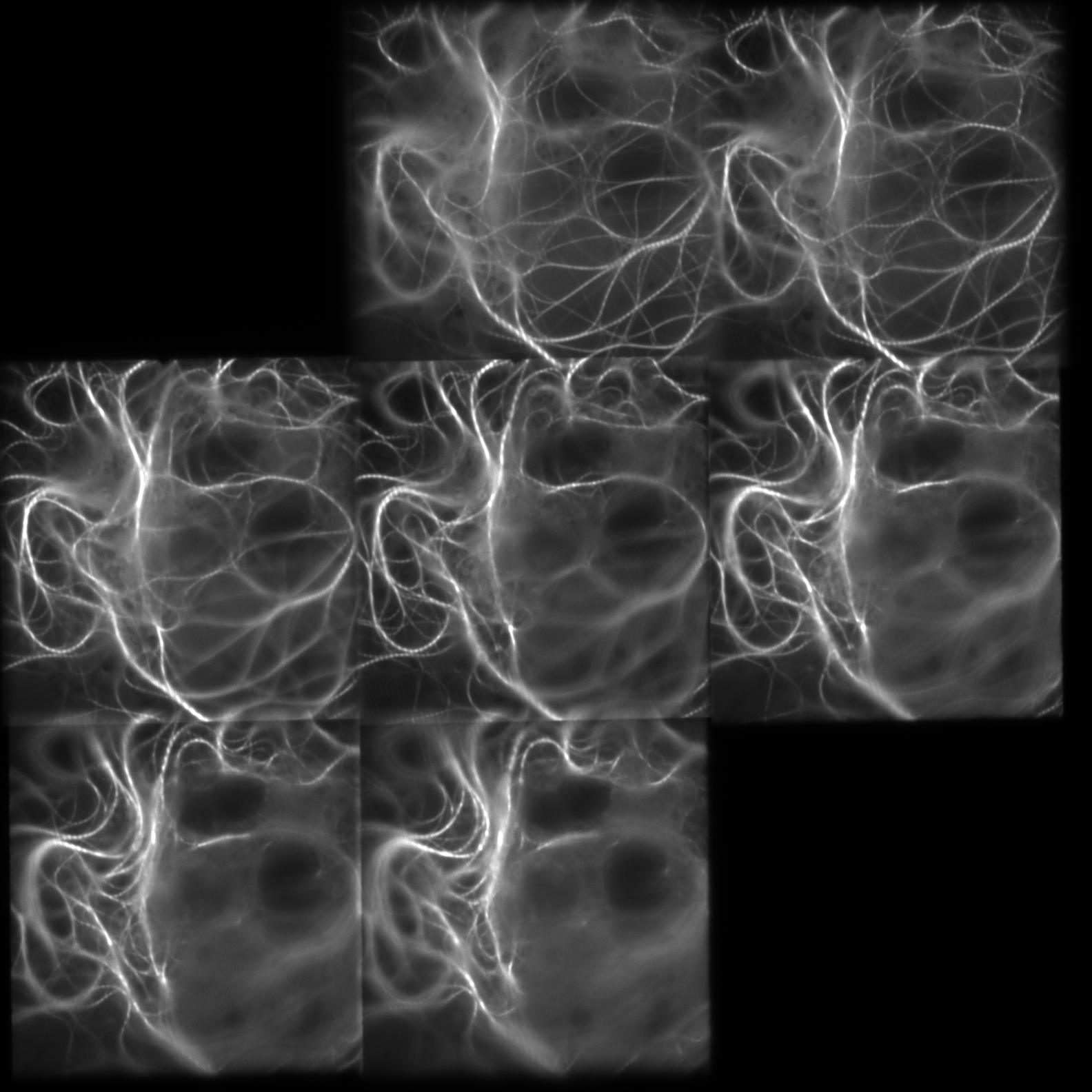

Supplement: Supplementary file 3 [file boe-15-4-2281-d002.zip › fig2/tubulin/raw/img_channel000_position000_time000000010_z000.tif]

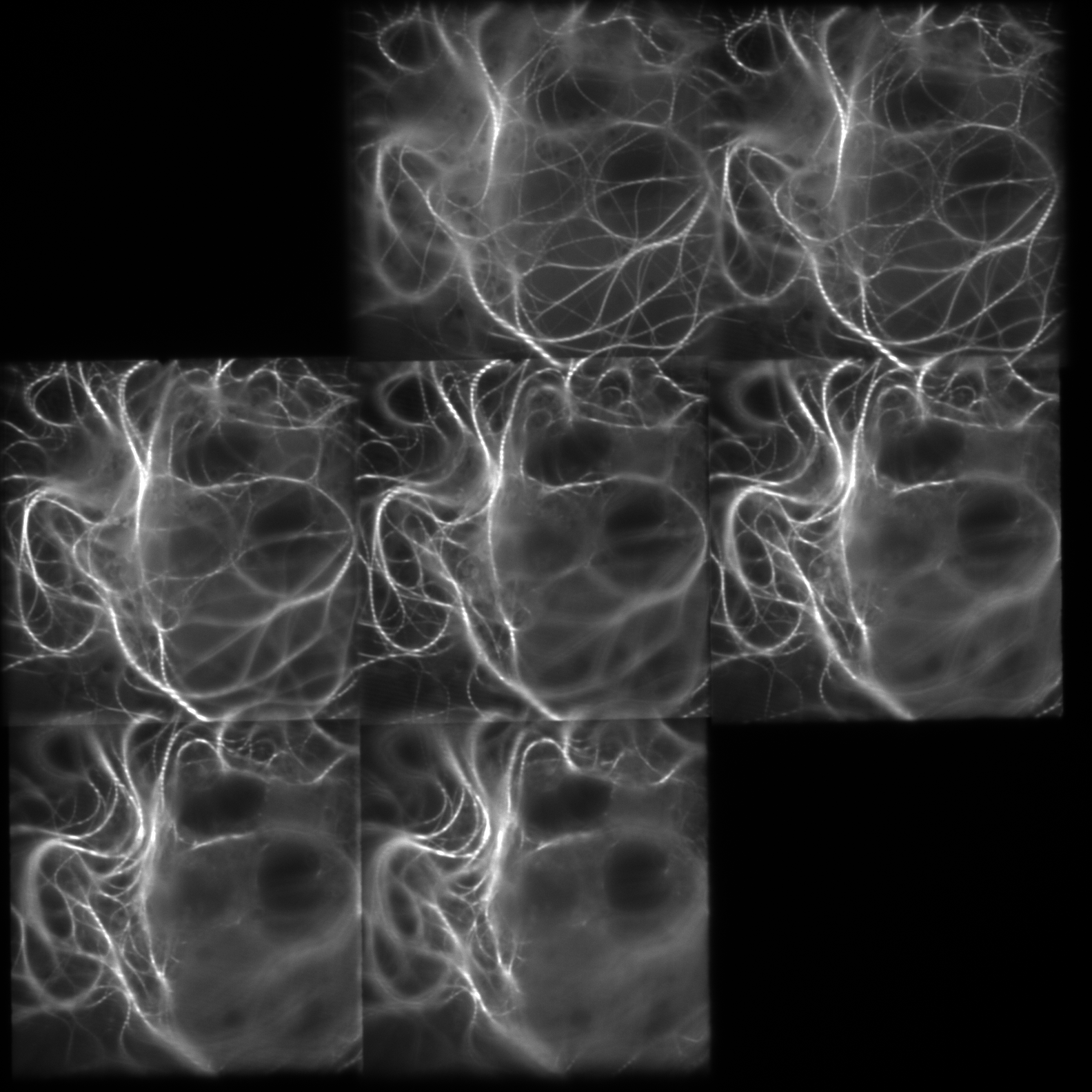

Supplement: Supplementary file 3 [file boe-15-4-2281-d002.zip › fig2/tubulin/raw/img_channel000_position000_time000000006_z000.tif]

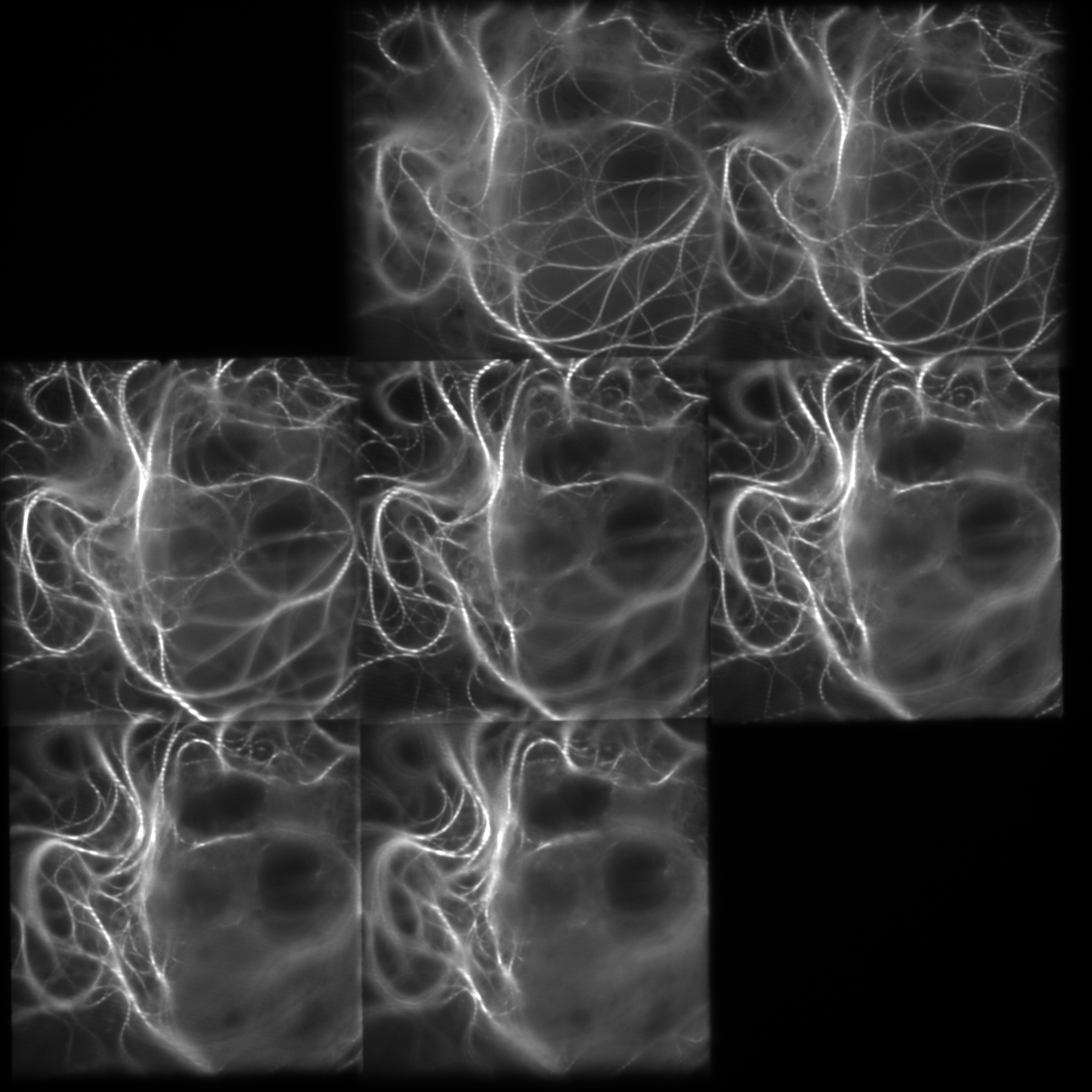

Supplement: Supplementary file 3 [file boe-15-4-2281-d002.zip › fig2/tubulin/raw/img_channel000_position000_time000000007_z000.tif]

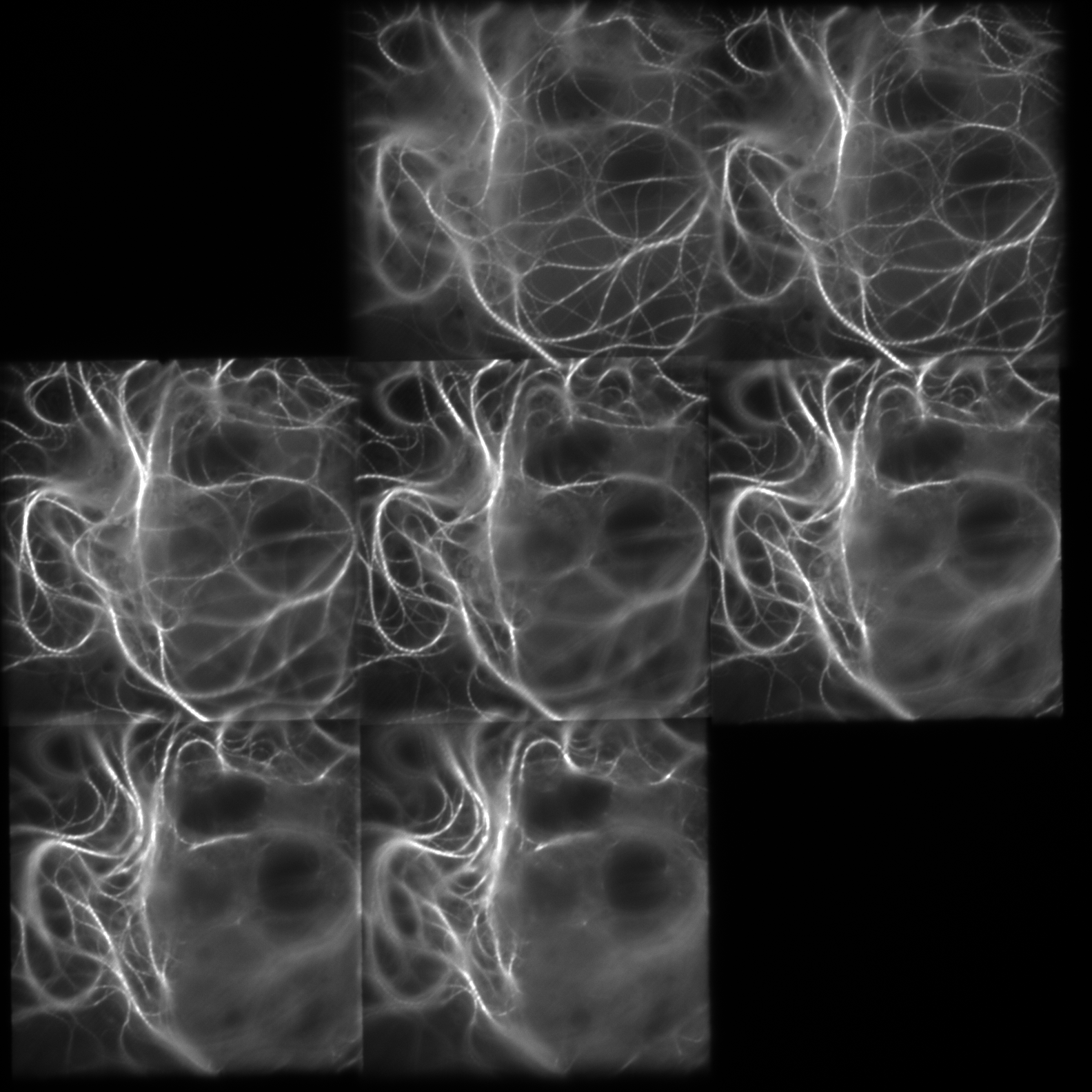

Supplement: Supplementary file 3 [file boe-15-4-2281-d002.zip › fig2/tubulin/raw/img_channel000_position000_time000000001_z000.tif]

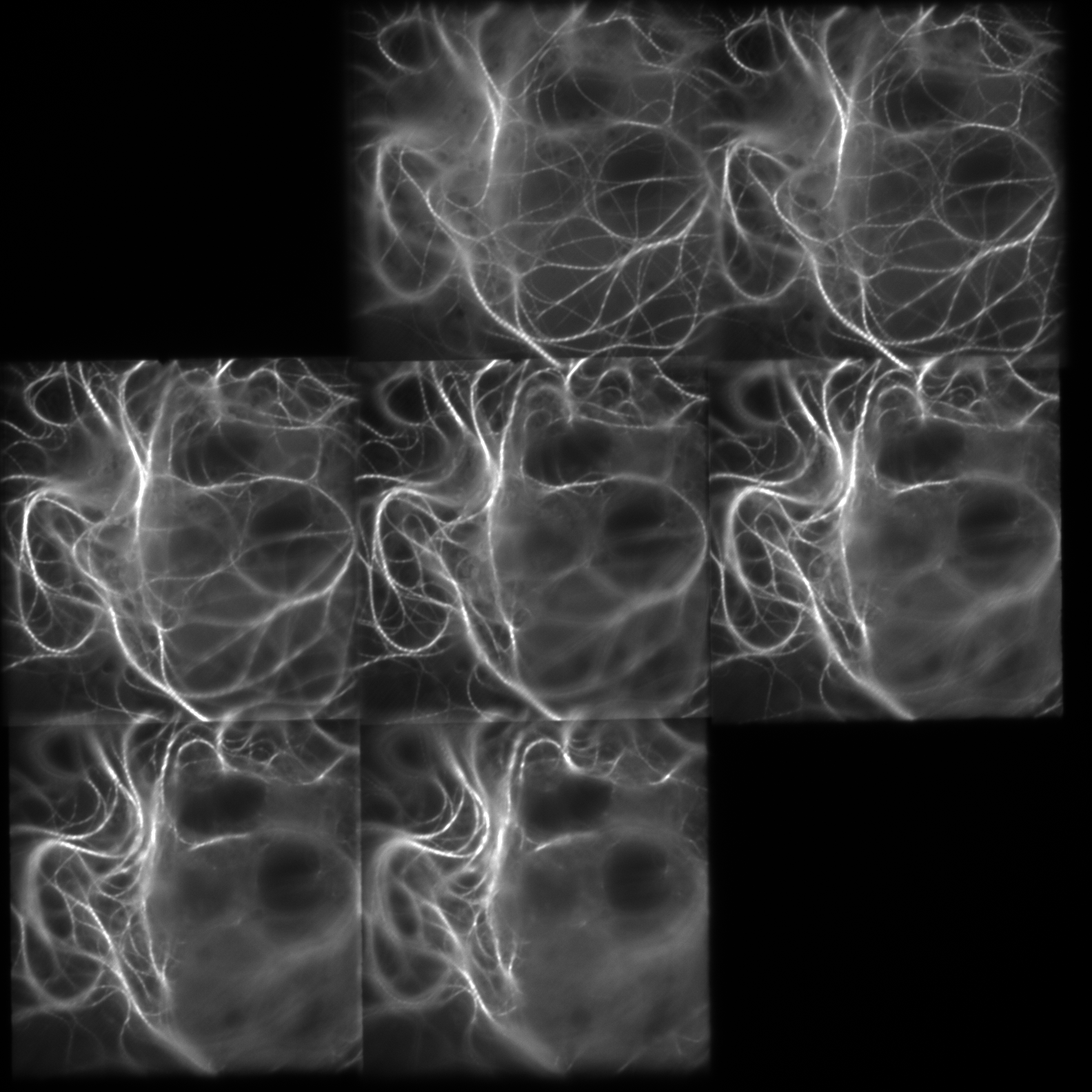

Supplement: Supplementary file 3 [file boe-15-4-2281-d002.zip › fig2/tubulin/raw/img_channel000_position000_time000000000_z000.tif]

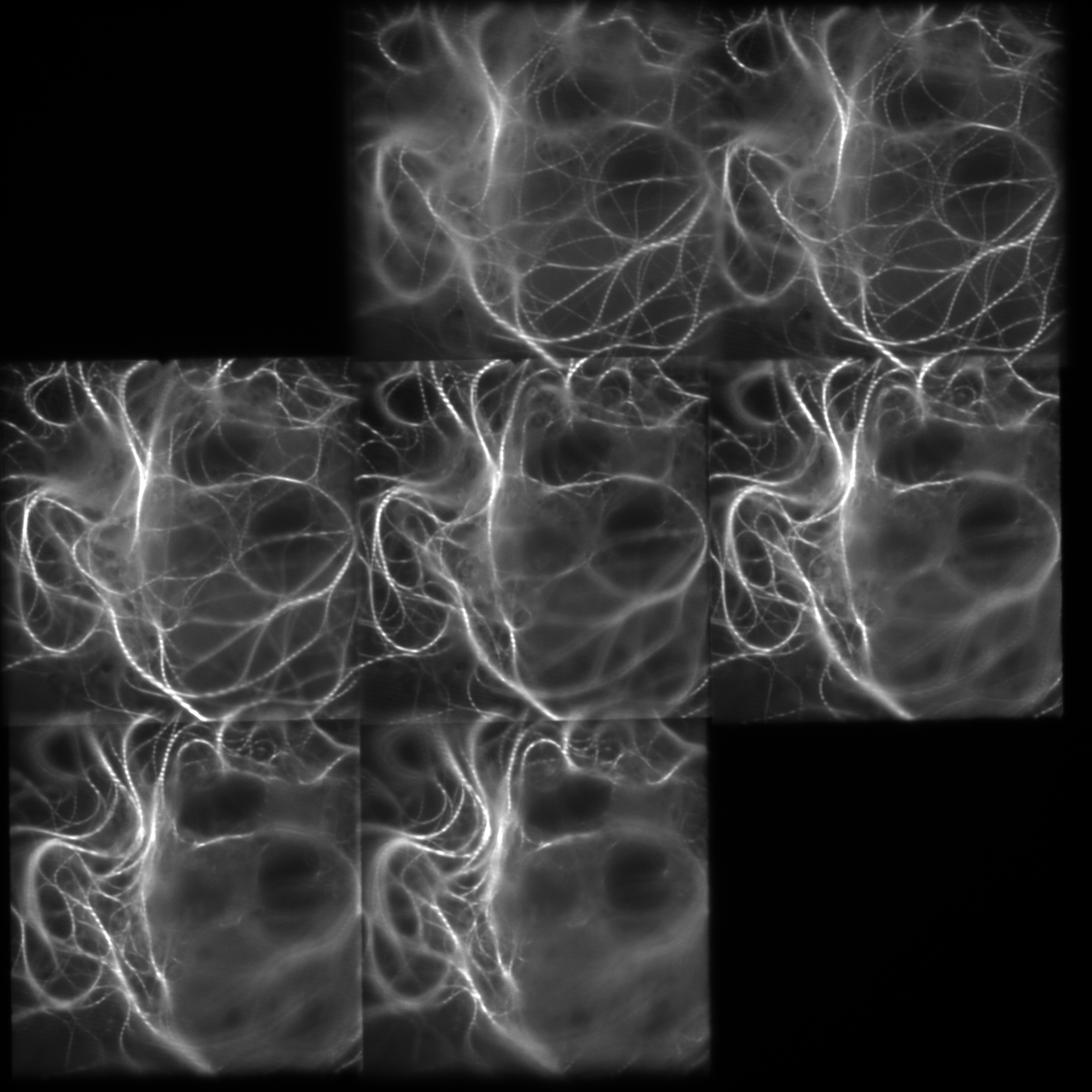

Supplement: Supplementary file 3 [file boe-15-4-2281-d002.zip › fig2/tubulin/raw/img_channel000_position000_time000000022_z000.tif]

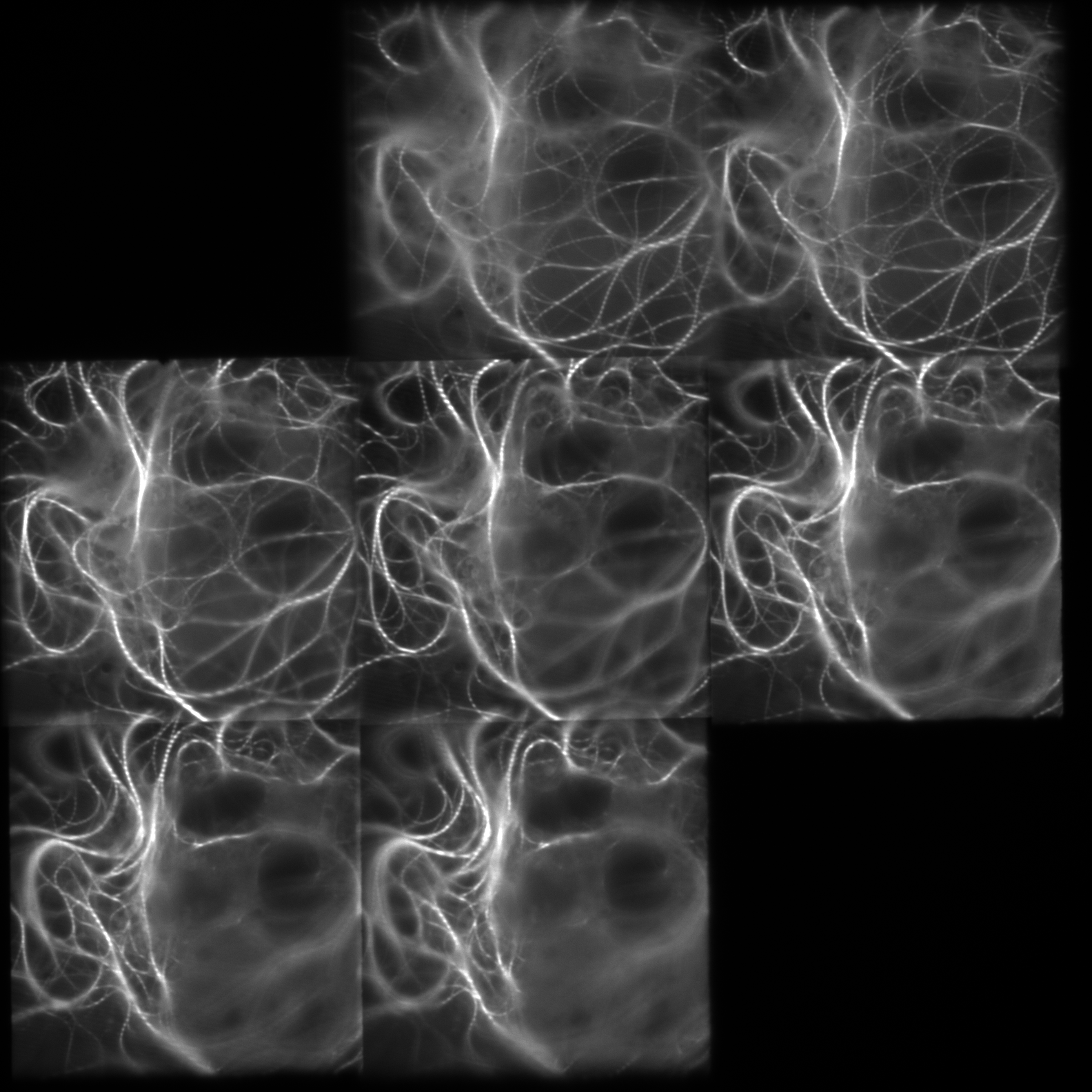

Supplement: Supplementary file 3 [file boe-15-4-2281-d002.zip › fig2/tubulin/raw/img_channel000_position000_time000000023_z000.tif]

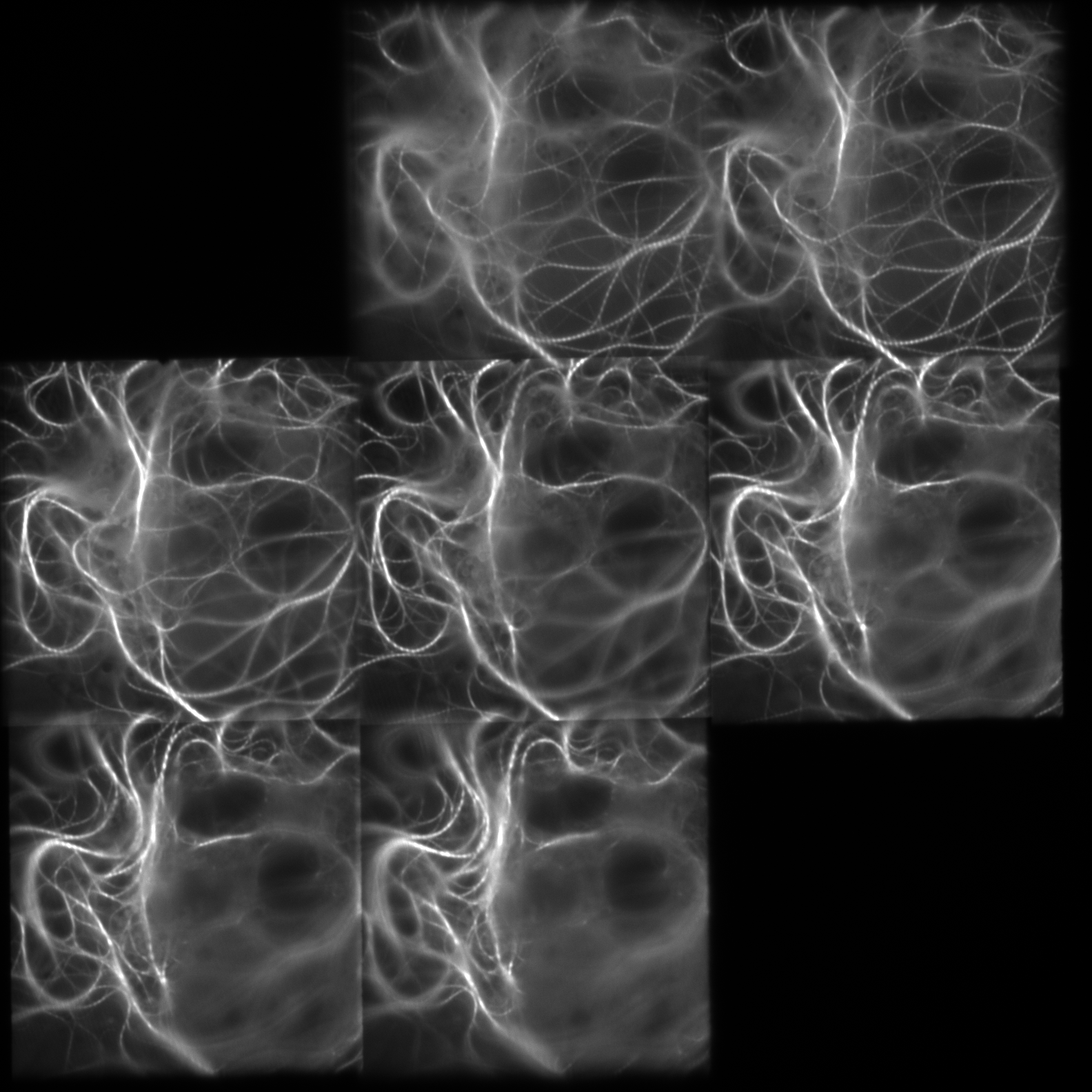

Supplement: Supplementary file 3 [file boe-15-4-2281-d002.zip › fig2/tubulin/raw/img_channel000_position000_time000000028_z000.tif]

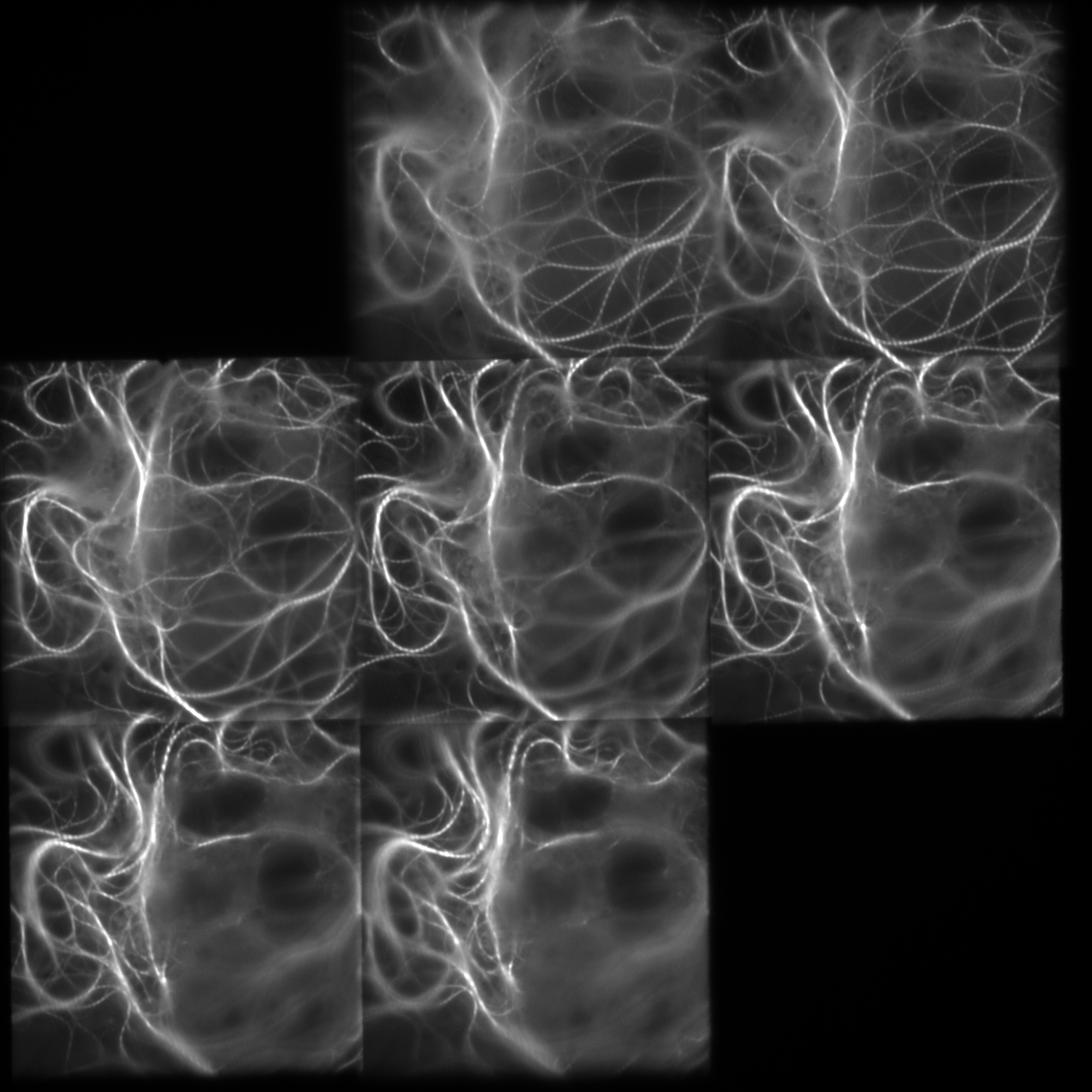

Supplement: Supplementary file 3 [file boe-15-4-2281-d002.zip › fig2/tubulin/raw/img_channel000_position000_time000000029_z000.tif]

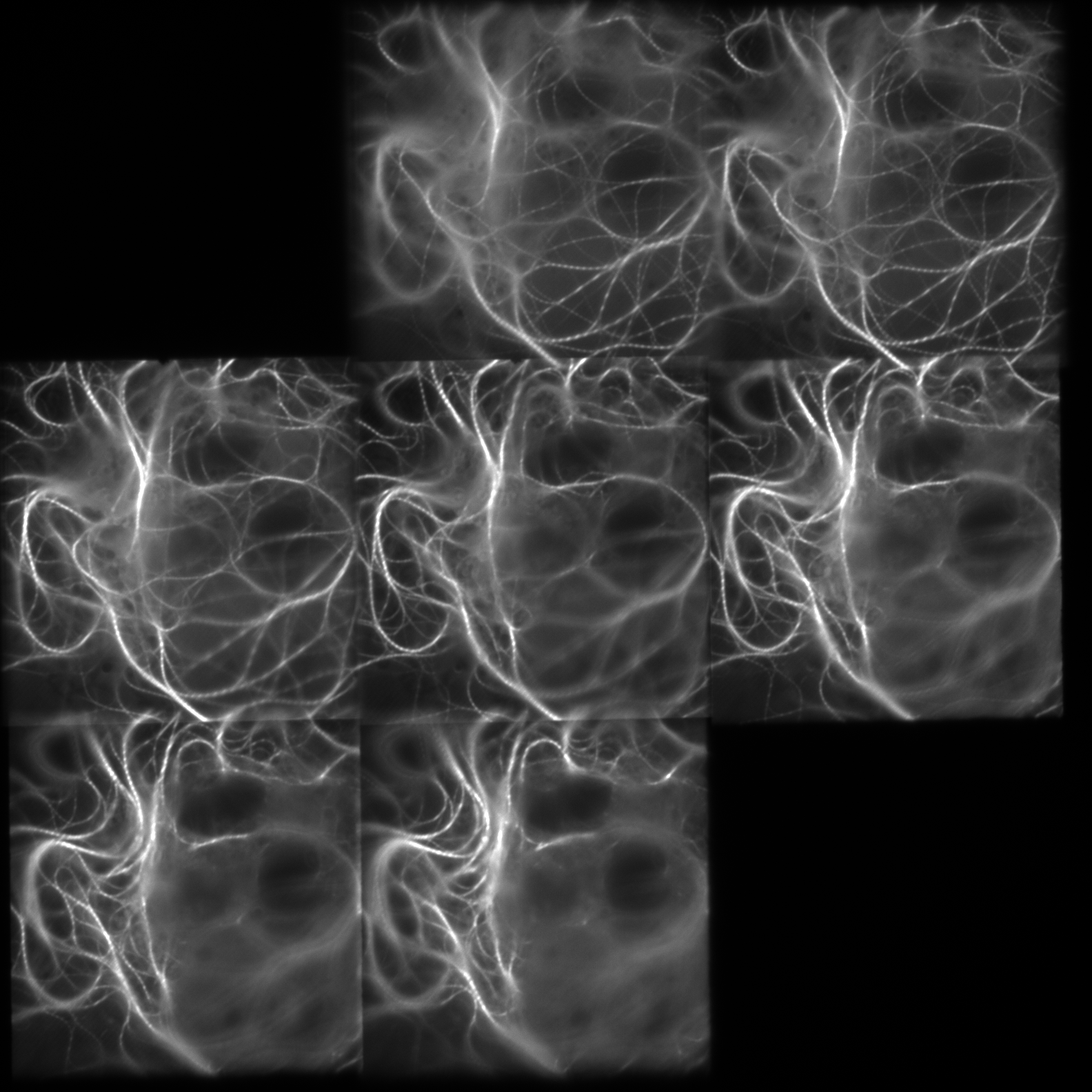

Supplement: Supplementary file 3 [file boe-15-4-2281-d002.zip › fig2/tubulin/raw/img_channel000_position000_time000000016_z000.tif]

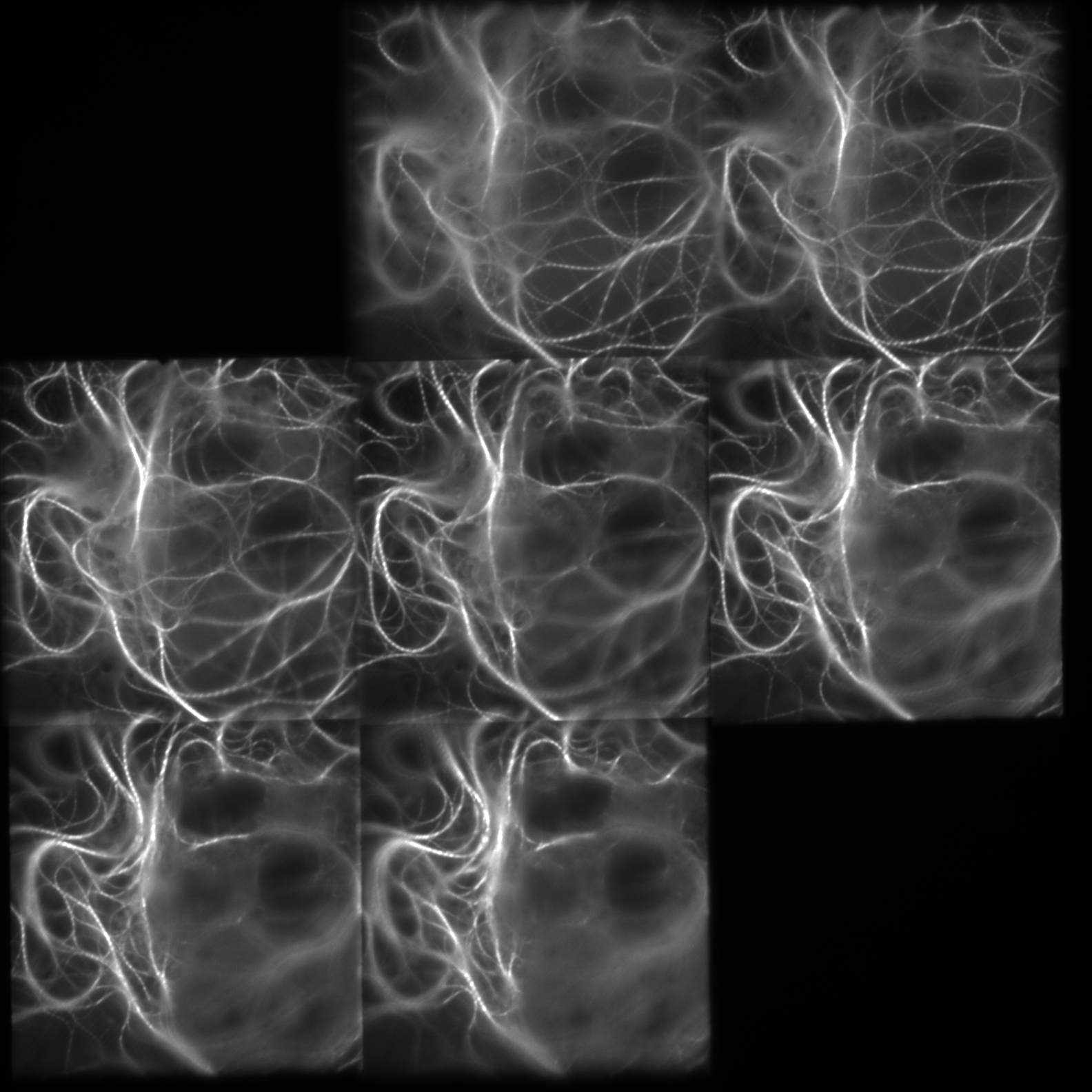

Supplement: Supplementary file 3 [file boe-15-4-2281-d002.zip › fig2/tubulin/raw/img_channel000_position000_time000000017_z000.tif]

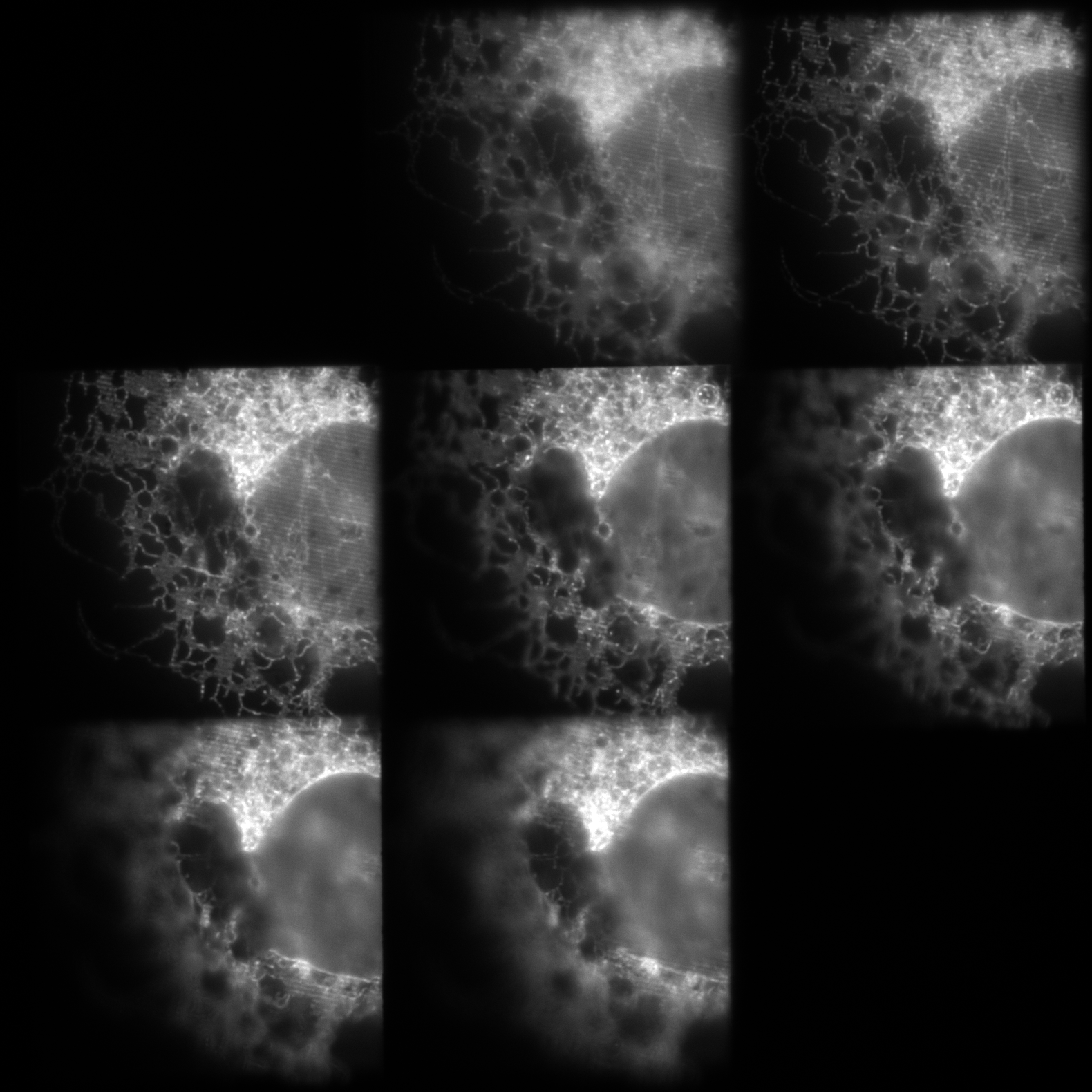

Supplement: Supplementary file 3 [file boe-15-4-2281-d002.zip › fig2/ER/raw/img_channel000_position000_time000000005_z000.tif]

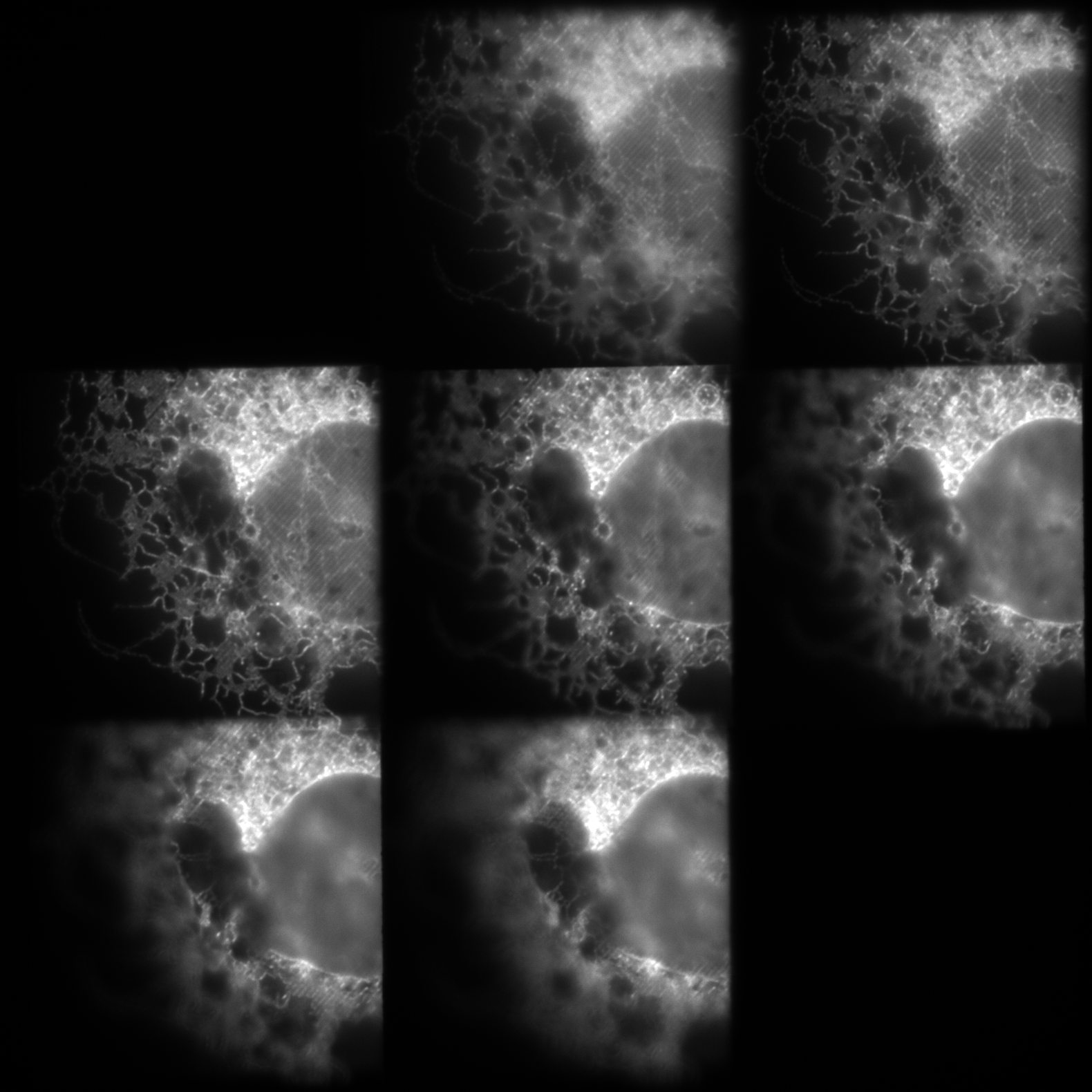

Supplement: Supplementary file 3 [file boe-15-4-2281-d002.zip › fig2/ER/raw/img_channel000_position000_time000000004_z000.tif]

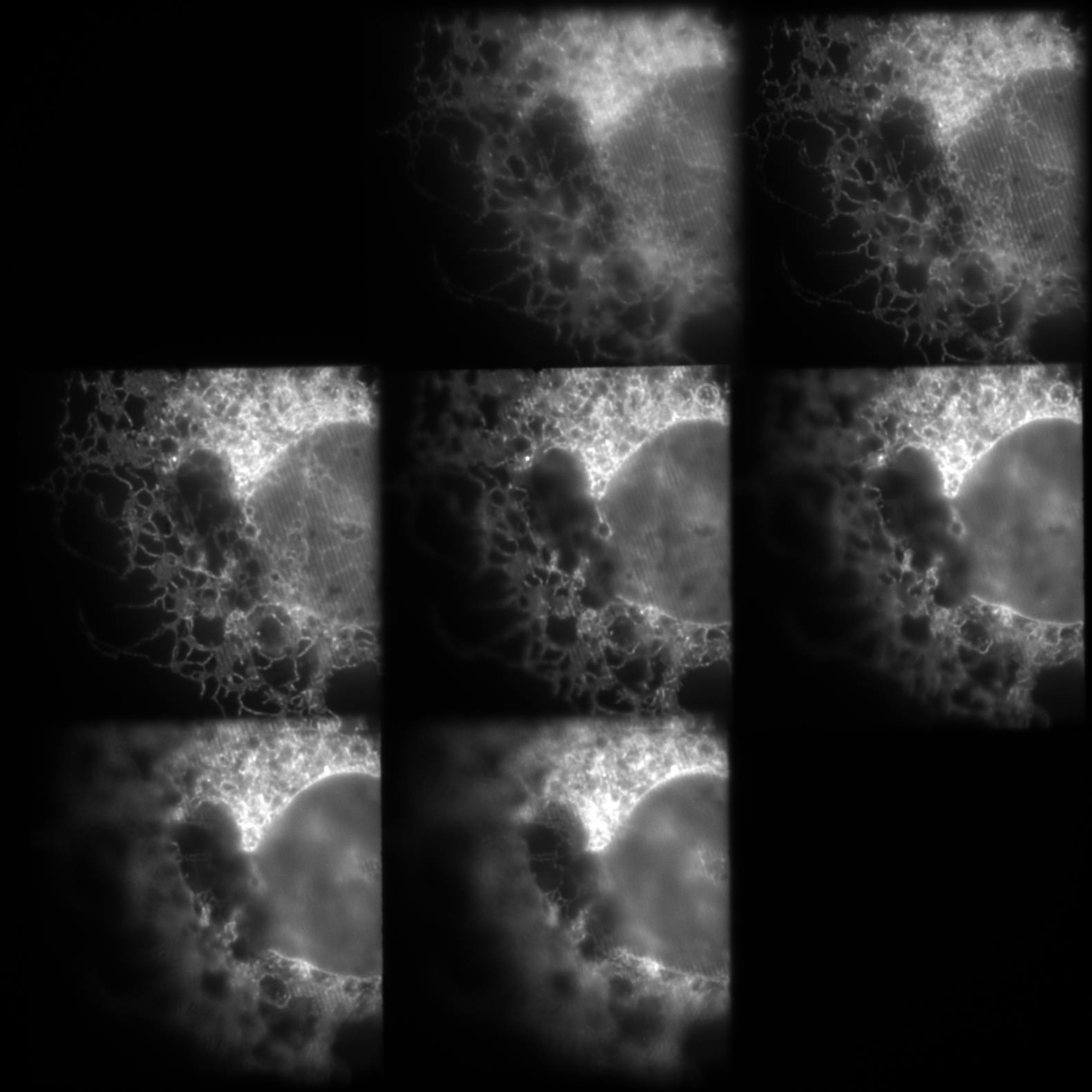

Supplement: Supplementary file 3 [file boe-15-4-2281-d002.zip › fig2/ER/raw/img_channel000_position000_time000000012_z000.tif]

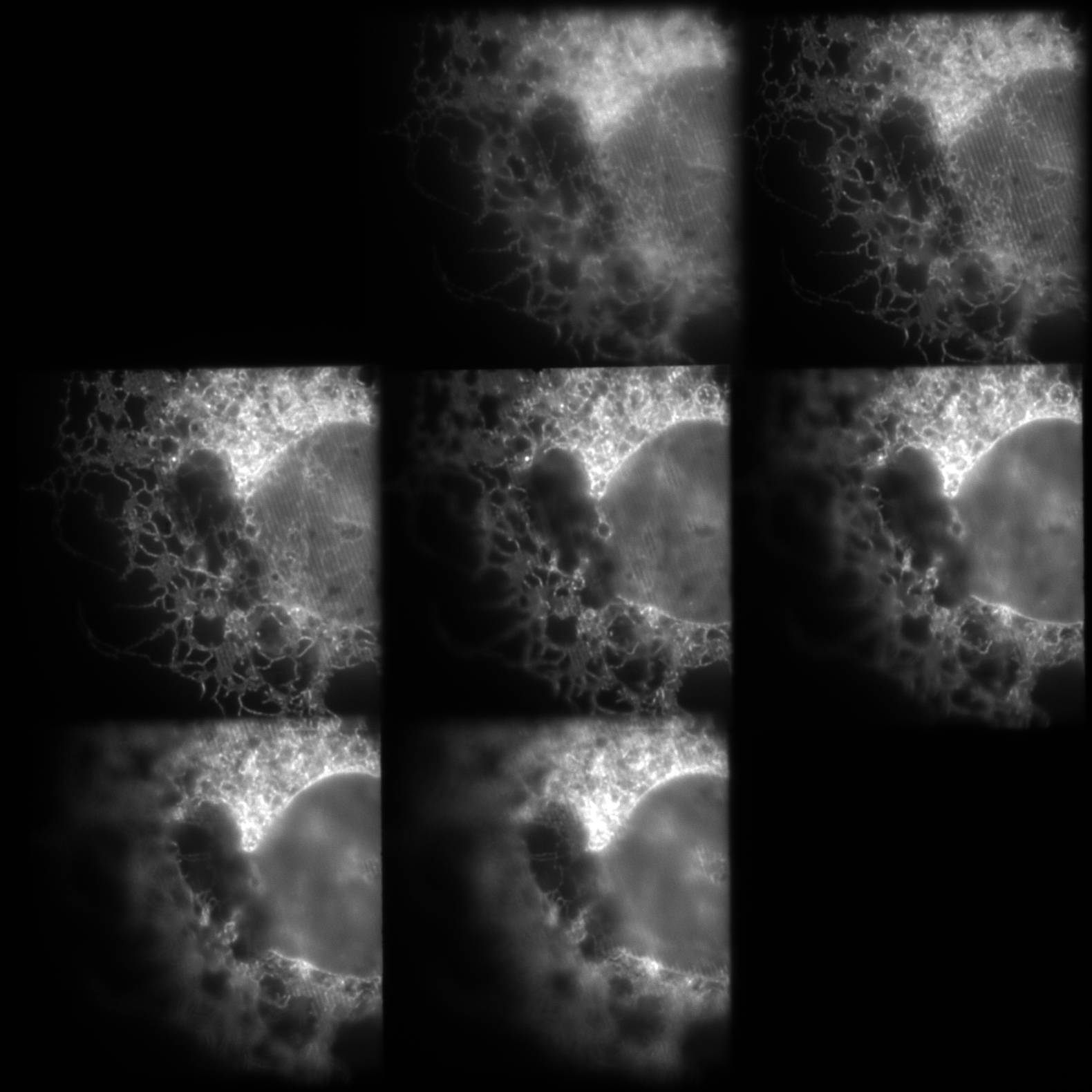

Supplement: Supplementary file 3 [file boe-15-4-2281-d002.zip › fig2/ER/raw/img_channel000_position000_time000000013_z000.tif]

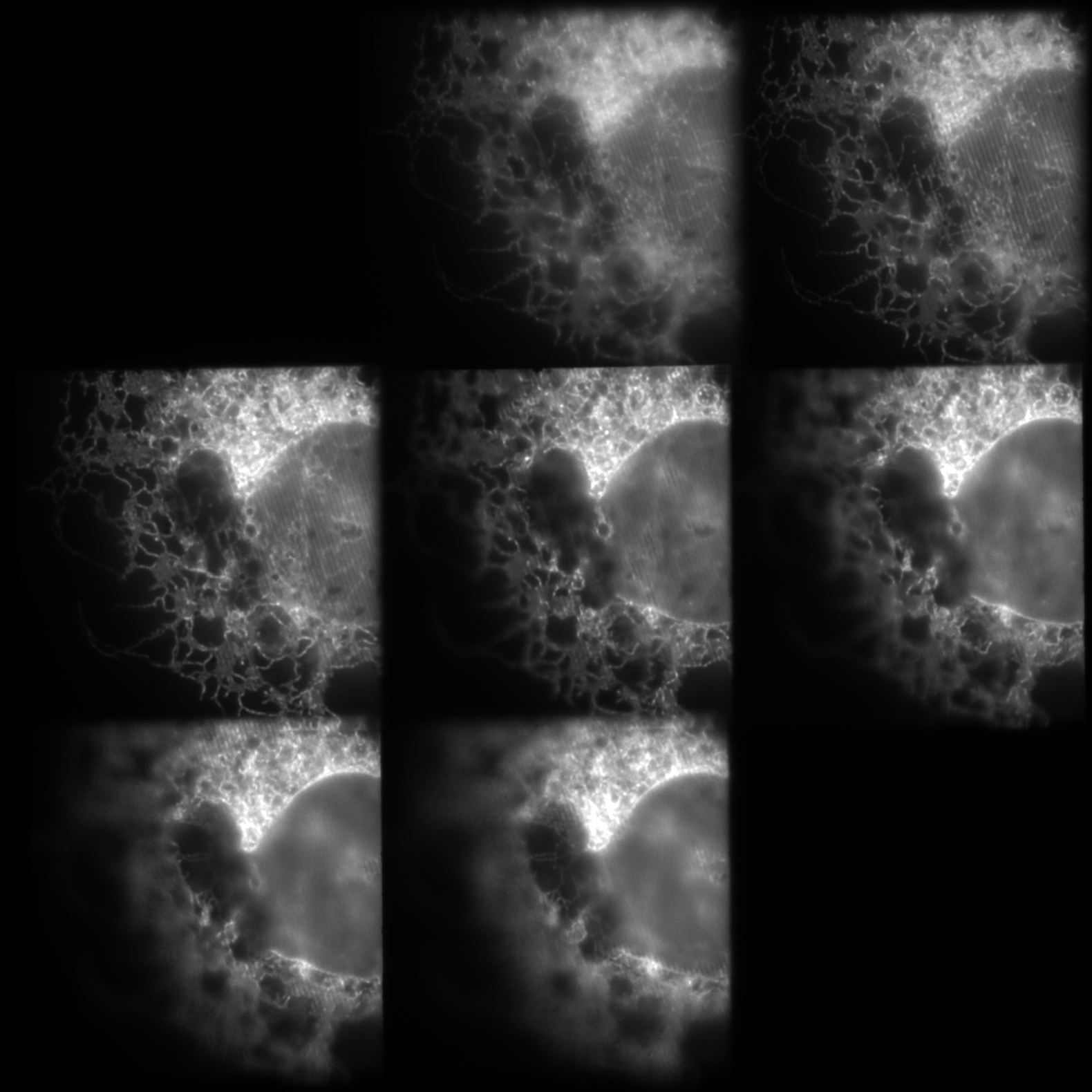

Supplement: Supplementary file 3 [file boe-15-4-2281-d002.zip › fig2/ER/raw/img_channel000_position000_time000000014_z000.tif]

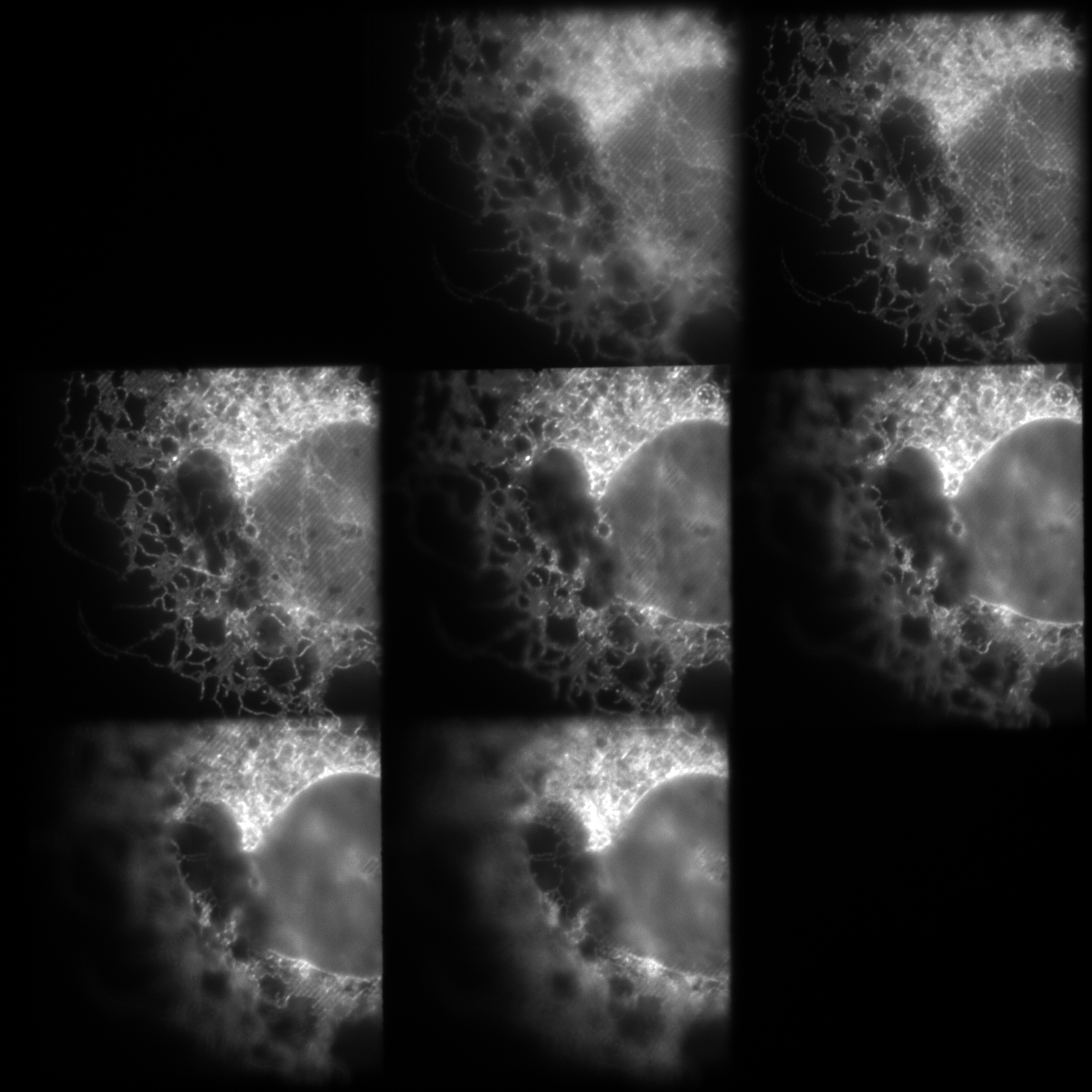

Supplement: Supplementary file 3 [file boe-15-4-2281-d002.zip › fig2/ER/raw/img_channel000_position000_time000000002_z000.tif]

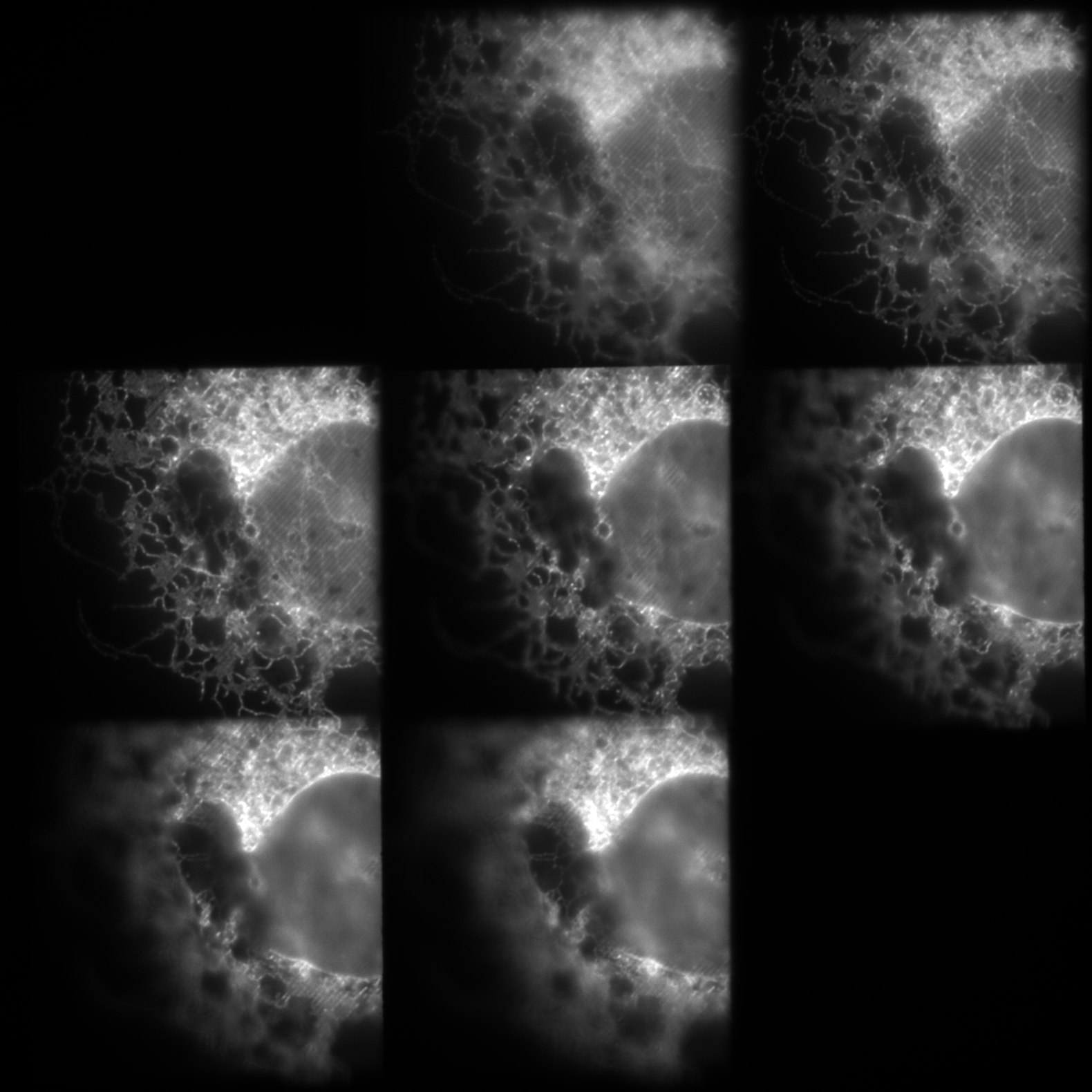

Supplement: Supplementary file 3 [file boe-15-4-2281-d002.zip › fig2/ER/raw/img_channel000_position000_time000000003_z000.tif]

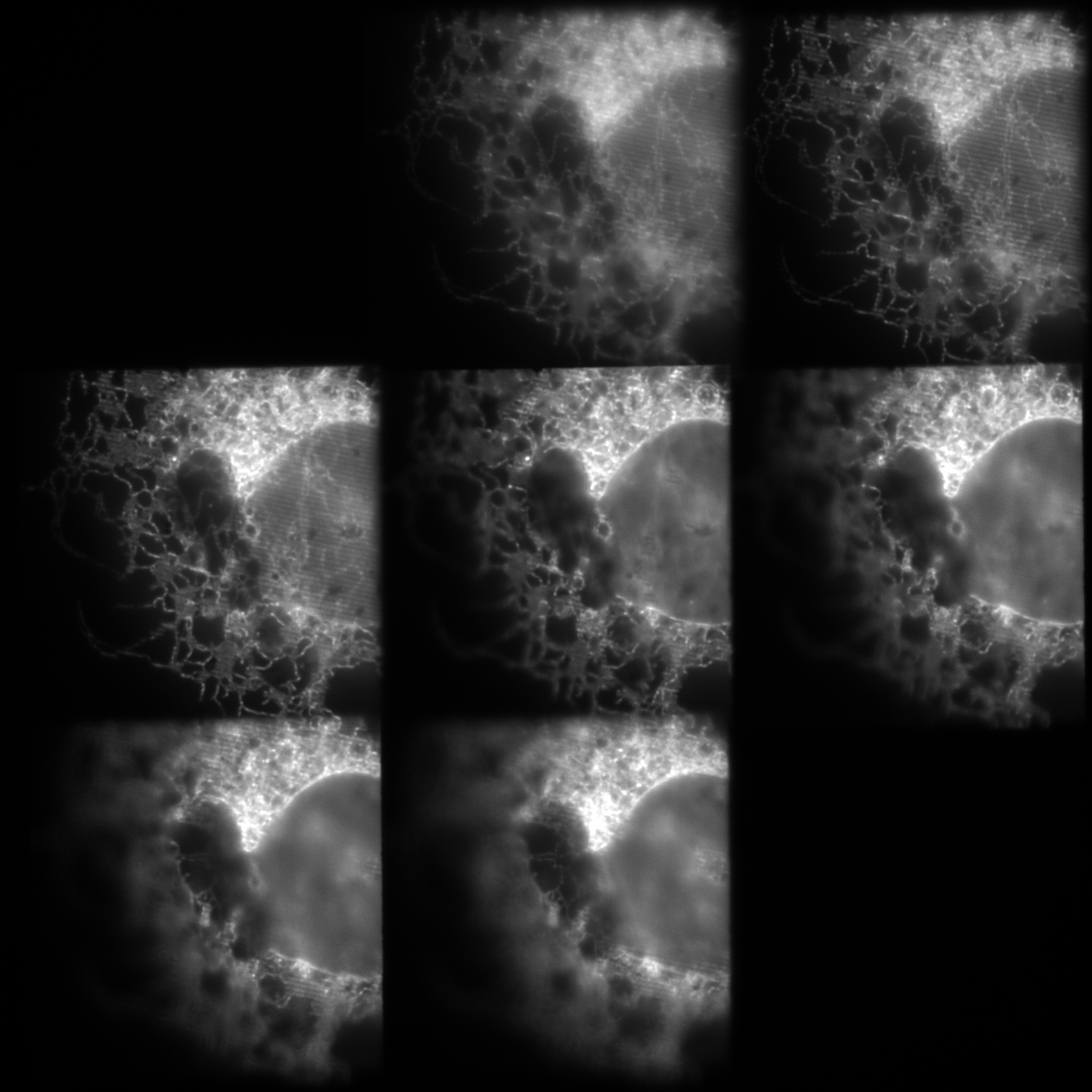

Supplement: Supplementary file 3 [file boe-15-4-2281-d002.zip › fig2/ER/raw/img_channel000_position000_time000000008_z000.tif]

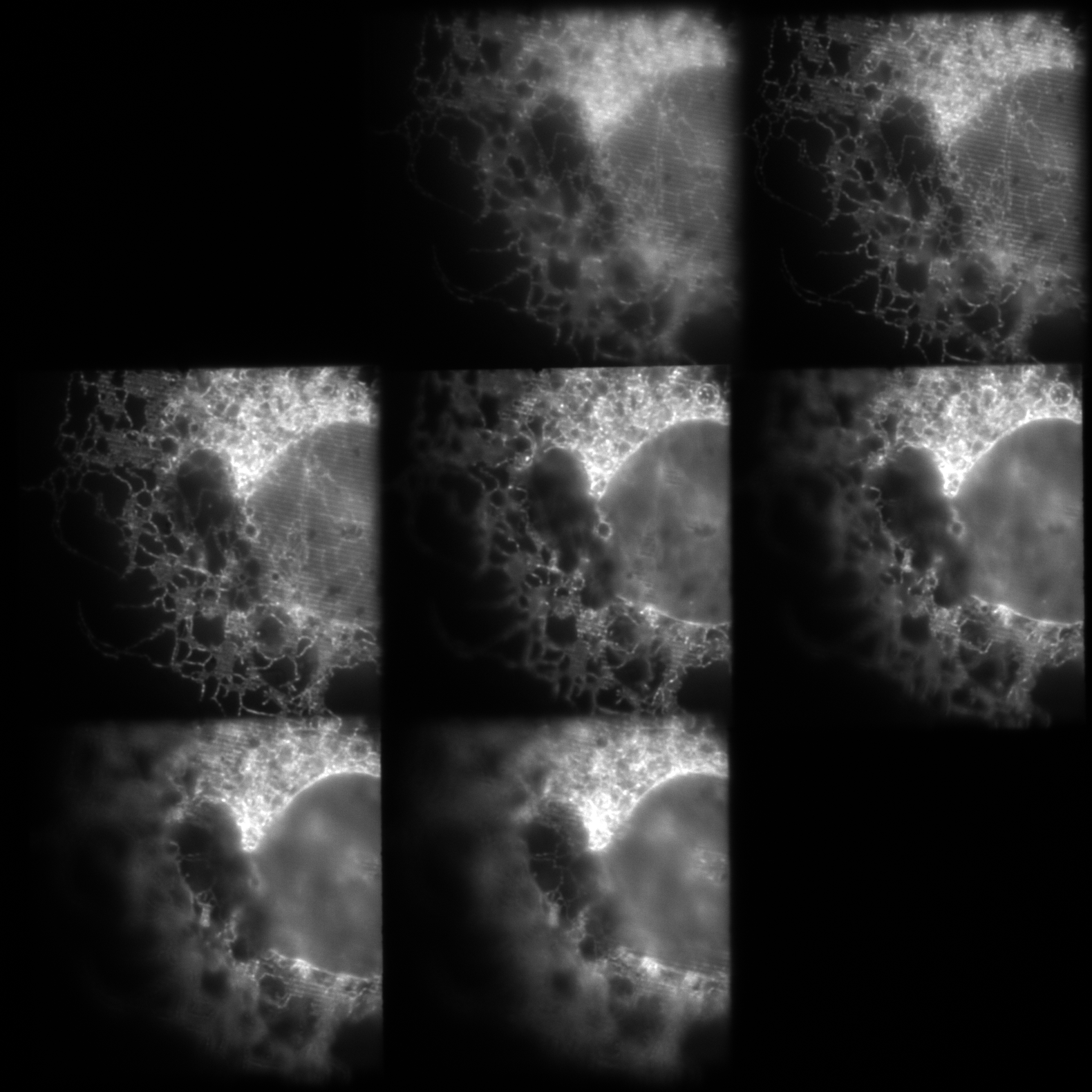

Supplement: Supplementary file 3 [file boe-15-4-2281-d002.zip › fig2/ER/raw/img_channel000_position000_time000000009_z000.tif]

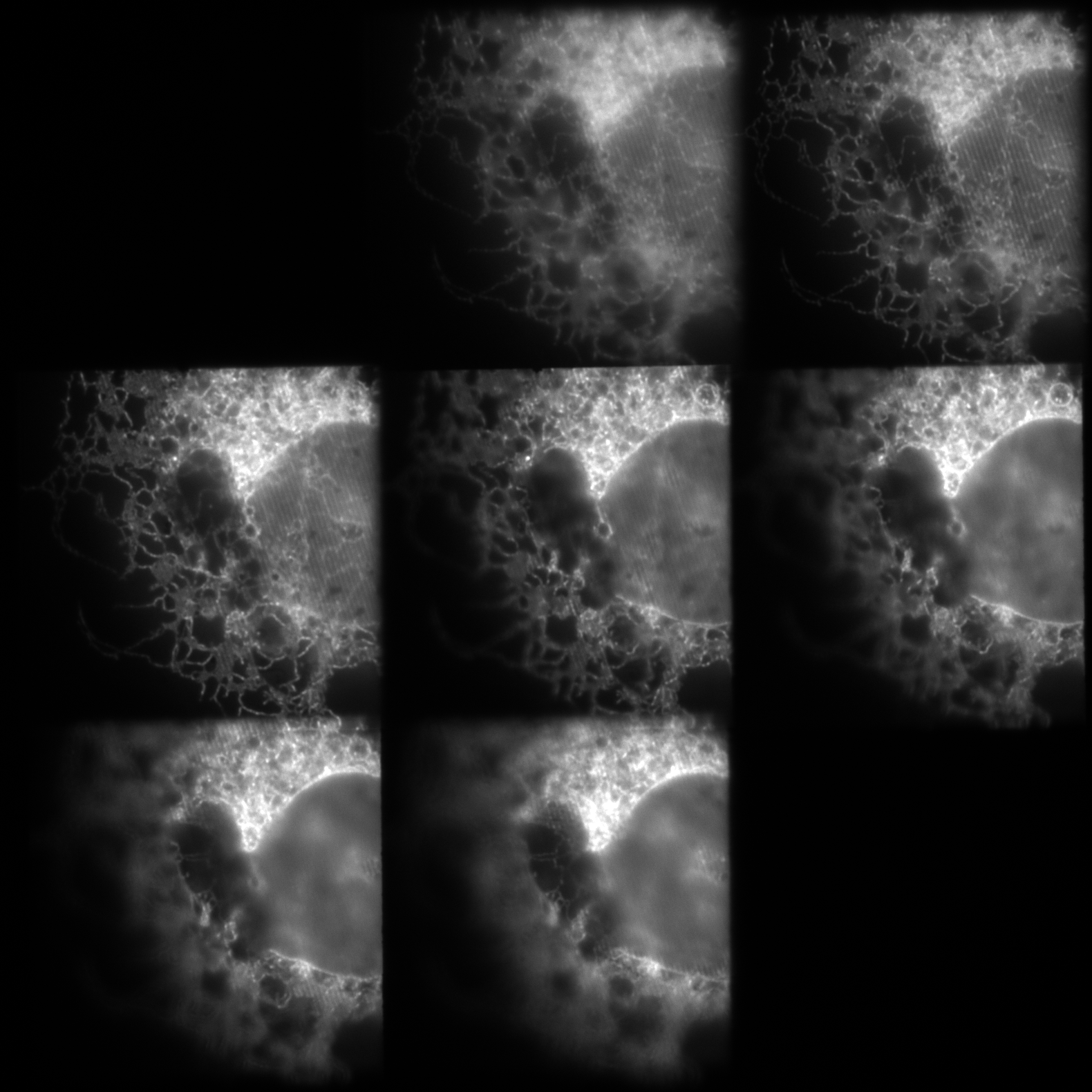

Supplement: Supplementary file 3 [file boe-15-4-2281-d002.zip › fig2/ER/raw/img_channel000_position000_time000000011_z000.tif]

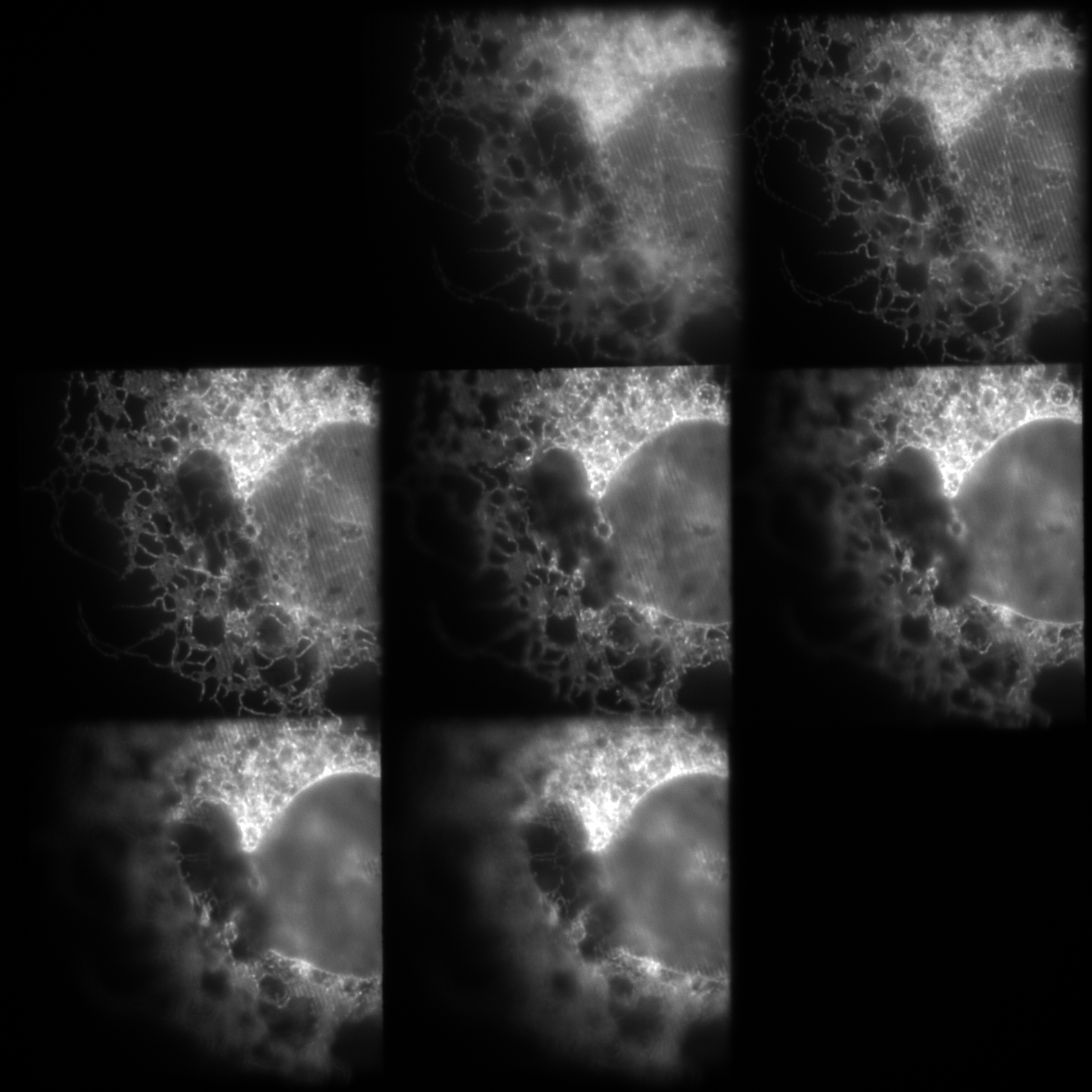

Supplement: Supplementary file 3 [file boe-15-4-2281-d002.zip › fig2/ER/raw/img_channel000_position000_time000000010_z000.tif]

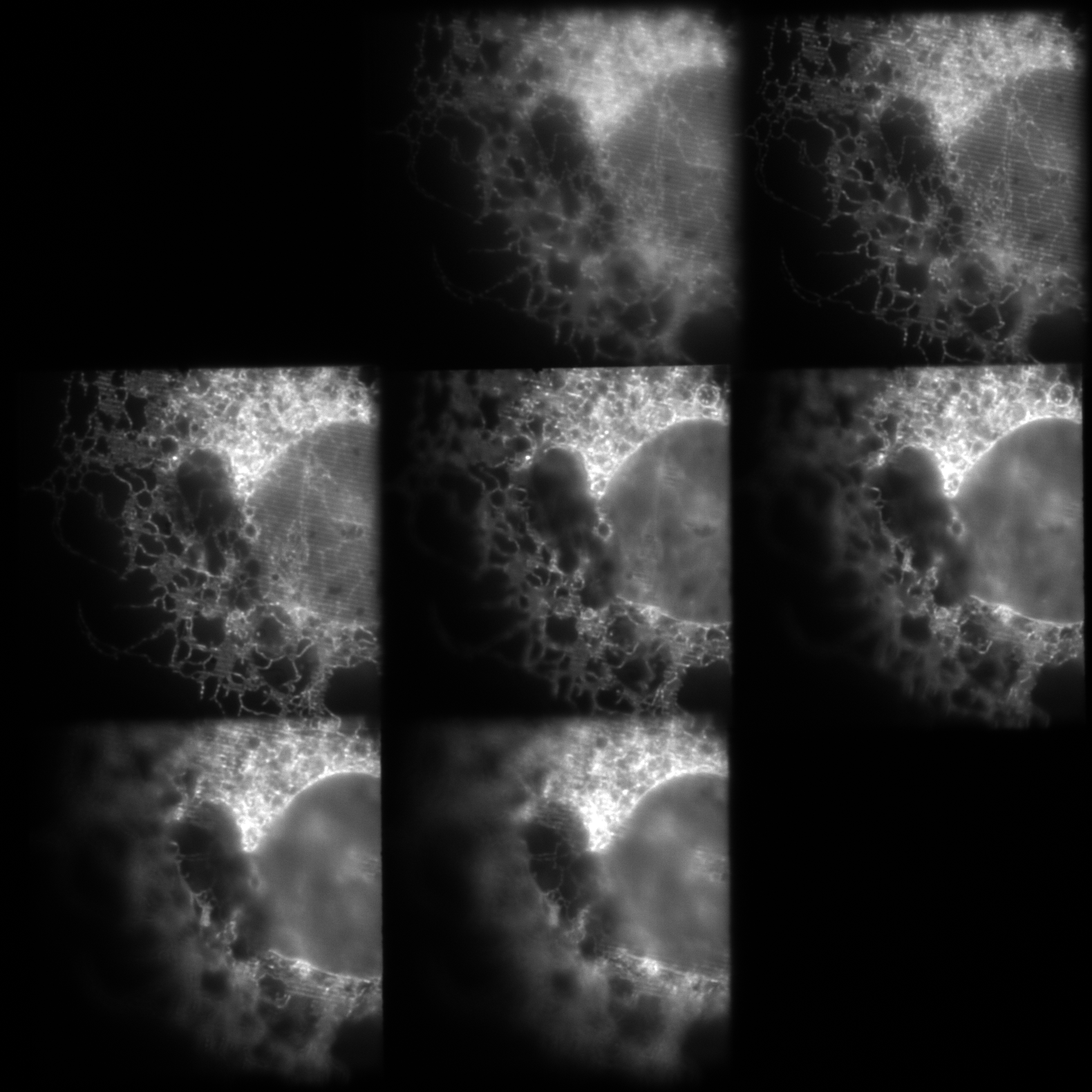

Supplement: Supplementary file 3 [file boe-15-4-2281-d002.zip › fig2/ER/raw/img_channel000_position000_time000000006_z000.tif]

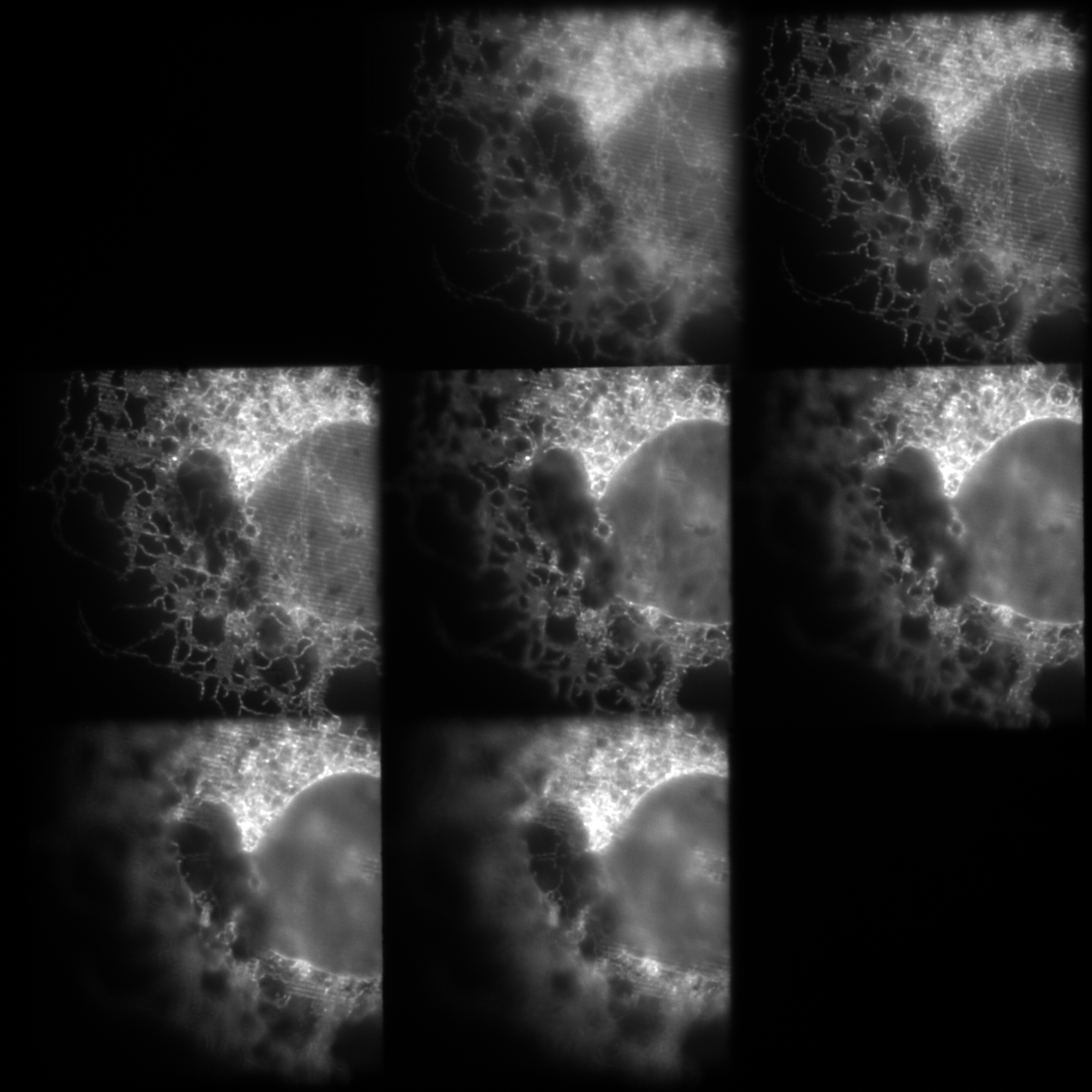

Supplement: Supplementary file 3 [file boe-15-4-2281-d002.zip › fig2/ER/raw/img_channel000_position000_time000000007_z000.tif]

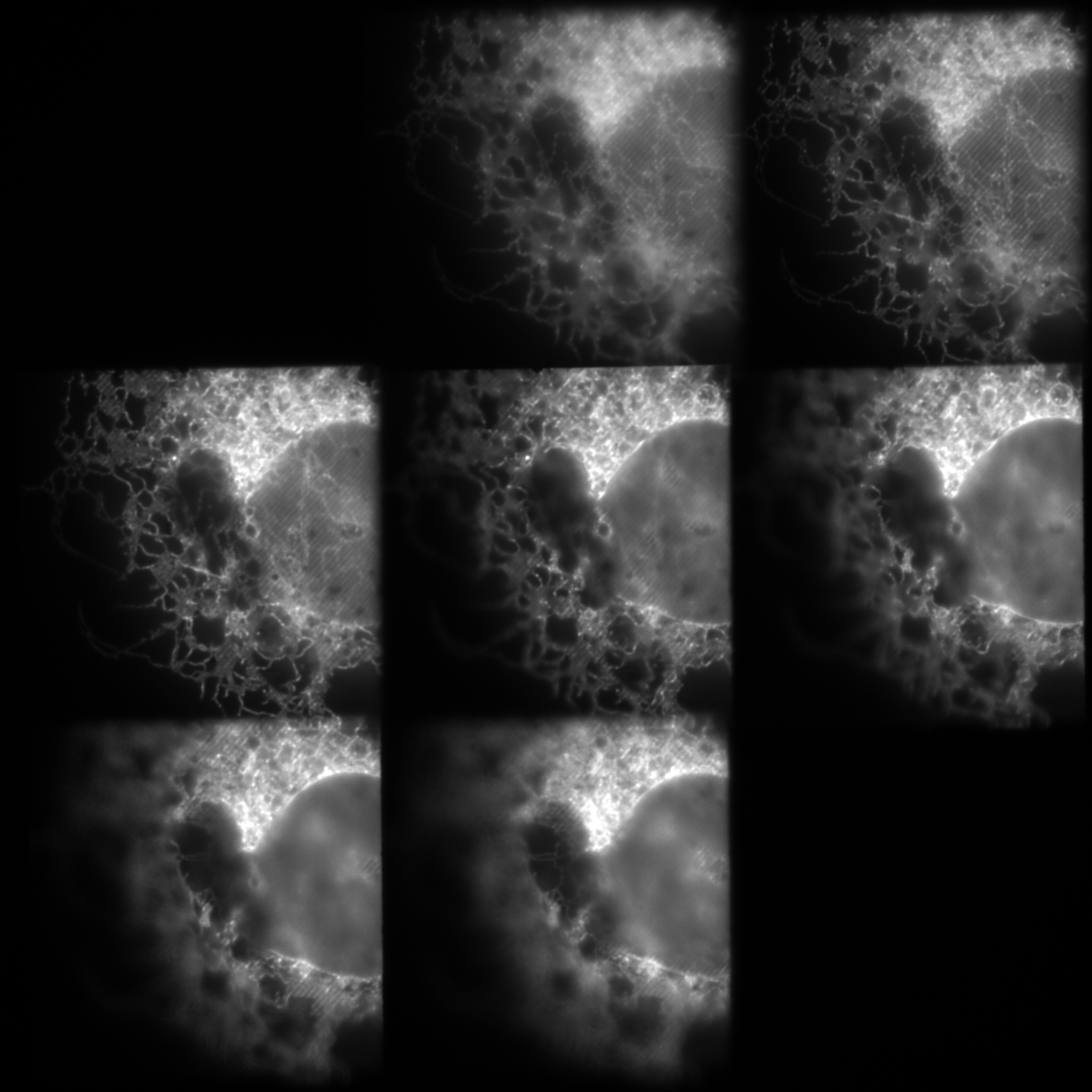

Supplement: Supplementary file 3 [file boe-15-4-2281-d002.zip › fig2/ER/raw/img_channel000_position000_time000000001_z000.tif]

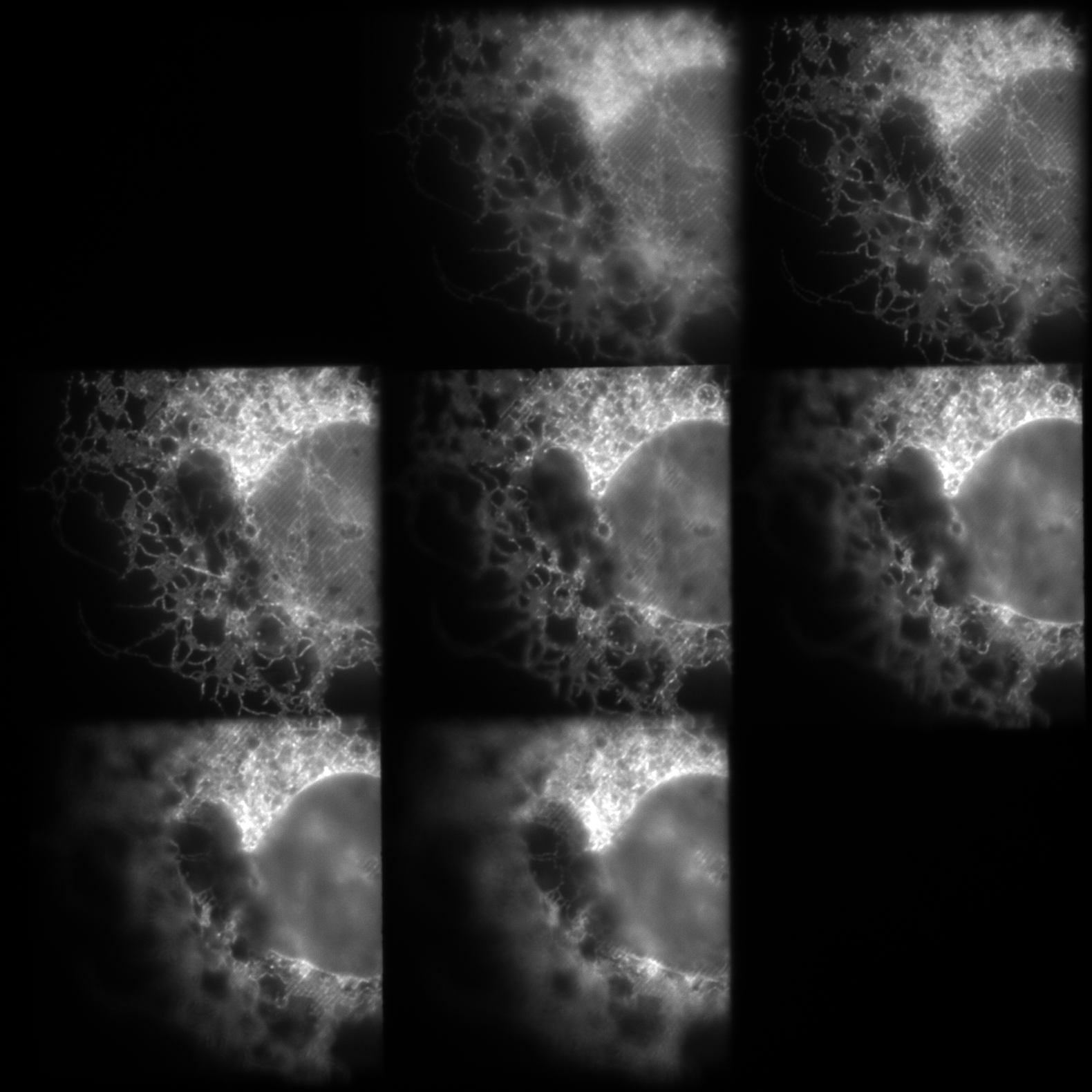

Supplement: Supplementary file 3 [file boe-15-4-2281-d002.zip › fig2/ER/raw/img_channel000_position000_time000000000_z000.tif]
